# Circulating Metabolites Associated with Alcohol Intake in the European Prospective Investigation into Cancer and Nutrition Cohort

**DOI:** 10.3390/nu10050654

**Published:** 2018-05-22

**Authors:** Eline H. van Roekel, Laura Trijsburg, Nada Assi, Marion Carayol, David Achaintre, Neil Murphy, Sabina Rinaldi, Julie A. Schmidt, Magdalena Stepien, Rudolf Kaaks, Tilman Kühn, Heiner Boeing, Khalid Iqbal, Domenico Palli, Vittorio Krogh, Rosario Tumino, Fulvio Ricceri, Salvatore Panico, Petra H. Peeters, Bas Bueno-de-Mesquita, Eva Ardanaz, Leila Lujan-Barroso, J. Ramón Quirós, José M. Huerta, Elena Molina-Portillo, Miren Dorronsoro, Konstantinos K. Tsilidis, Elio Riboli, Agnetha Linn Rostgaard-Hansen, Anne Tjønneland, Kim Overvad, Elisabete Weiderpass, Marie-Christine Boutron-Ruault, Gianluca Severi, Antonia Trichopoulou, Anna Karakatsani, Anastasia Kotanidou, Anders Håkansson, Johan Malm, Matty P. Weijenberg, Marc J. Gunter, Mazda Jenab, Mattias Johansson, Ruth C. Travis, Augustin Scalbert, Pietro Ferrari

**Affiliations:** 1 Department of Epidemiology, GROW School for Oncology and Developmental Biology, Maastricht University, 6229 HA Maastricht, The Netherlands; eline.vanroekel@maastrichtuniversity.nl (E.H.v.R.); mp.weijenberg@maastrichtuniversity.nl (M.P.W.); 2 Nutritional Methodology and Biostatistics Group, Nutrition and Metabolism Section, International Agency for Research on Cancer (IARC-WHO), 69372 Lyon, France; laura.trijsburg@wur.nl (L.T.); assin@students.iarc.fr (N.A.); 3 Epidaure, Prevention Department of the Institut régional du Cancer de Montpellier (ICM), 34298 Montpellier, France; Marion.Carayol@icm.unicancer.fr; 4 Laboratoire Epsylon, Paul Valery University of Montpellier, 34090 Montpellier, France; 5 Biomarkers Group, Nutrition and Metabolism Section, International Agency for Research on Cancer (IARC-WHO), 69372 Lyon, France; achaintred@iarc.fr (D.A.); rinaldis@iarc.fr (S.R.); ScalbertA@iarc.fr (A.S.); 6 Nutritional Epidemiology Group, Nutrition and Metabolism Section, International Agency for Research on Cancer (IARC-WHO), 69372 Lyon, France; MurphyN@iarc.fr (N.M.); StepienM@iarc.fr (M.S.); GunterM@iarc.fr (M.J.G.); jenabm@iarc.fr (M.J.); 7 Cancer Epidemiology Unit, Nuffield Department of Population Health, University of Oxford, Oxford OX3 7LF, UK; julie.schmidt@ndph.ox.ac.uk (J.A.S.); ruth.travis@ndph.ox.ac.uk (R.C.T.); 8 Division of Cancer Epidemiology, German Cancer Research Center (DKFZ), 69120 Heidelberg, Germany; r.kaaks@dkfz-heidelberg.de (R.K.); t.kuehn@Dkfz-Heidelberg.de (T.K.); 9 Department of Epidemiology, German Institute of Human Nutrition Potsdam-Rehbruecke, 14558 Nuthetal, Germany; boeing@dife.de (H.B.); khalid.iqbal@dife.de (K.I.); 10 Cancer Risk Factors and Life-Style Epidemiology Unit, Cancer Research and Prevention Institute—ISPO, 50141 Florence, Italy; d.palli@ispo.toscana.it; 11 Epidemiology and Prevention Unit, Fondazione IRCCS Istituto Nazionale dei Tumori, 20133 Milan, Italy; Vittorio.Krogh@istitutotumori.mi.it; 12 Cancer Registry and Histopathology Department, Civic—M.P.Arezzo Hospital, ASP, 97100 Ragusa, Italy; rtumino@tin.it; 13 Department of Clinical and Biological Sciences, University of Turin, 10124 Turin, Italy; fulvio.ricceri@unito.it; 14 Unit of Epidemiology, Regional Health Service ASL TO3, 10095 Turin, Italy; 15 Dipartimento di Medicina Clinica e Chirurgia, Federico II University, 80138 Naples, Italy; spanico@unina.it; 16 Department of Epidemiology, Julius Center for Health Sciences and Primary Care, University Medical Center Utrecht, Utrecht University, 3508 GA Utrecht, The Netherlands; P.H.M.Peeters@umcutrecht.nl; 17 Former Senior Scientist, Dept. for Determinants of Chronic Diseases (DCD), National Institute for Public Health and the Environment (RIVM), 3721 MA Bilthoven, The Netherlands; basbuenodemesquita@gmail.com; 18 Former Associate Professor, Department of Gastroenterology and Hepatology, University Medical Center Utrecht, 3584 CX Utrecht, The Netherlands; 19 Visiting Professor, Dept. of Epidemiology and Biostatistics, The School of Public Health, Imperial College, London SW7 2AZ, UK; 20 Department of Social & Preventive Medicine, Faculty of Medicine, University of Malaya, Kuala Lumpur 50603, Malaysia; 21 Navarra Public Health Institute, 31003 Pamplona, Spain; me.ardanaz.aicua@cfnavarra.es; 22 IdiSNA, Navarra Institute for Health Research, 31008 Pamplona, Spain; 23 CIBER de Epidemiología y Salud Pública (CIBERESP), 28029 Madrid, Spain; jmhuerta.carm@gmail.com (J.M.H.); elena.molina.easp@juntadeandalucia.es (E.M.-P.); 24 Unit of Nutrition and Cancer, Cancer Epidemiology Research Program, Catalan Institute of Oncology-IDIBELL, L’Hospitalet de Llobregat, 08908 Barcelona, Spain; llujan@iconcologia.net; 25 Public Health Directorate, 33005 Oviedo, Spain; epic_asturias@asturias.org; 26 Department of Epidemiology, Murcia Regional Health Council, IMIB-Arrixaca, 30008 Murcia, Spain; 27 Escuela Andaluza de Salud Pública. Instituto de Investigación Biosanitaria ibs, GRANADA, Hospitales Universitarios de Granada/Universidad de Granada, 18010 Granada, Spain; 28 Basque Regional Health Department, Public Health Direction and Biodonostia Research Institute CIBERESP, 20014 Donostia, Spain; m-dorronsoro@euskadi.eus; 29 Department of Epidemiology and Biostatistics, School of Public Health, Imperial College London, London SW7 2AZ, UK; ktsilidis@gmail.com (K.K.T.); e.riboli@imperial.ac.uk (E.R.); 30 Department of Hygiene and Epidemiology, University of Ioannina School of Medicine, 45110 Ioannina, Greece; 31 Danish Cancer Society Research Center, 2100 Copenhagen, Denmark; agnrha@cancer.dk (A.L.R.-H.); annet@CANCER.DK (A.T.); 32 Department of Public Health, Section for Epidemiology, Aarhus University, 8000 Aarhus, Denmark; ko@ph.au.dk; 33 Department of Cardiology, Aalborg University Hospital, 9100 Aalborg, Denmark; 34 Department of Community Medicine, Faculty of Health Sciences, University of Tromsø, The Arctic University of Norway, 9019 Tromsø, Norway; Elisabete.Weiderpass.Vainio@ki.se; 35 Department of Research, Cancer Registry of Norway, Institute of Population-Based Cancer Research, NO-0304 Oslo, Norway; 36 Department of Medical Epidemiology and Biostatistics, Karolinska Institutet, 171 77 Stockholm, Sweden; 37 Genetic Epidemiology Group, Folkhälsan Research Center, 00290 Helsinki, Finland; 38 CESP “Health across Generations”, INSERM, Univ Paris-Sud, UVSQ, Univ Paris-Saclay, 94800 Villejuif, France; marie-christine.boutron@gustaveroussy.fr (M.-C.B.-R.); gianluca.severi@gustaveroussy.fr (G.S.); 39 Gustave Roussy, 94800 Villejuif, France; 40 Cancer Epidemiology Centre, Cancer Council Victoria and Centre for Epidemiology and Biostatistics, Melbourne School of Population and Global Health, The University of Melbourne, Melbourne, VIC 3010, Australia; 41 Hellenic Health Foundation, 115 27 Athens, Greece; atrichopoulou@hhf-greece.gr (A.T.); a.karakatsani@hhf-greece.gr (A.Ka.); akotanid@gmail.com (A.Ko.); 42 WHO Collaborating Center for Nutrition and Health, Unit of Nutritional Epidemiology and Nutrition in Public Health, Department of Hygiene, Epidemiology and Medical Statistics, School of Medicine, National and Kapodistrian University of Athens, 157 72 Athens, Greece; 43 2nd Pulmonary Medicine Department, School of Medicine, National and Kapodistrian University of Athens, “ATTIKON” University Hospital, 124 62 Haidari, Greece; 44 1st Department of Critical Care Medicine & Pulmonary Services, University of Athens Medical School, Evangelismos Hospital, 10675 Athens, Greece; 45 Lund University, Faculty of Medicine, Department of Clinical Sciences Lund, Psychiatry, SE-221 00 Lund, Sweden; anders_c.hakansson@med.lu.se; 46 Department of Translational Medicine, Clinical Chemistry, Lund University, Skåne University Hospital, 205 02 Malmö, Sweden; johan.malm@med.lu.se; 47 Genetic Epidemiology Group, Section of Genetics, International Agency for Research on Cancer (IARC-WHO), 69372 Lyon, France; johanssonm@iarc.fr

**Keywords:** alcohol, targeted metabolomics, lipid metabolites, amino acids, acylcarnitines

## Abstract

Identifying the metabolites associated with alcohol consumption may provide insights into the metabolic pathways through which alcohol may affect human health. We studied associations of alcohol consumption with circulating concentrations of 123 metabolites among 2974 healthy participants from the European Prospective Investigation into Cancer and Nutrition (EPIC) study. Alcohol consumption at recruitment was self-reported through dietary questionnaires. Metabolite concentrations were measured by tandem mass spectrometry (BIOCRATES AbsoluteIDQTM p180 kit). Data were randomly divided into discovery (2/3) and replication (1/3) sets. Multivariable linear regression models were used to evaluate confounder-adjusted associations of alcohol consumption with metabolite concentrations. Metabolites significantly related to alcohol intake in the discovery set (FDR *q*-value < 0.05) were further tested in the replication set (Bonferroni-corrected *p*-value < 0.05). Of the 72 metabolites significantly related to alcohol intake in the discovery set, 34 were also significant in the replication analysis, including three acylcarnitines, the amino acid citrulline, four lysophosphatidylcholines, 13 diacylphosphatidylcholines, seven acyl-alkylphosphatidylcholines, and six sphingomyelins. Our results confirmed earlier findings that alcohol consumption was associated with several lipid metabolites, and possibly also with specific acylcarnitines and amino acids. This provides further leads for future research studies aiming at elucidating the mechanisms underlying the effects of alcohol in relation to morbid conditions.

## 1. Introduction

The harmful use of alcohol is among the main modifiable risk factors for human morbidity, disability, and mortality worldwide [1]. It has been associated with over 200 health conditions including neuropsychiatric conditions, liver cirrhosis, several cancers, hypertensive heart disease, fetal alcohol syndrome, and infectious diseases [1]. Moderate alcohol intake was found to be inversely associated with the risk of type 2 diabetes [2] and heterogeneous associations were observed for cardiovascular disease subtypes, with an inverse association with coronary heart disease, and a positive association with stroke [3,4]. Each year, alcohol causes approximately 3.3 million deaths, i.e., one in every 20 deaths in the world [1]. A better understanding of metabolic pathways affected by alcohol consumption may contribute to the development of mechanism-tailored intervention strategies to prevent and treat alcohol-related conditions (e.g., through identification of pharmacotherapy targets). Furthermore, it may help to identify biomarkers of alcohol consumption facilitating early preventive strategies in individuals at risk of developing alcohol-related morbidities.

Metabolomics is the measurement of the dynamic metabolic responses of a living system to pathophysiological and other stimuli, through a comprehensive characterization of molecules of endogenous and exogenous origin in biological samples [5]. Applying high-throughput metabolomics approaches in population-based studies can be used to examine how environmental factors, such as alcohol consumption, are associated with human metabolism [6,7]. Targeted metabolomics approaches focus on measuring concentrations of predefined panels of metabolites involved in certain biochemical pathways of the human body [8]. This enables the identification of metabolites within those panels that are associated with alcohol consumption, possibly providing insight into the metabolic pathways through which alcohol may exert adverse health effects.

To our knowledge, three previous population-based studies have investigated associations of total alcohol intake [9,10] and/or consumption of specific alcoholic beverages [11] with circulating concentrations of a panel of metabolites using a targeted metabolomics approach (BIOCRATES AbsoluteIDQTM p150 kit). These studies were conducted in German populations (*n* = 2090 [9] and *n* = 1030 [10]), and in over 3500 female twins from the United Kingdom [11], and mainly observed associations of self-reported alcohol intake with phospholipid and sphingolipid metabolism. Two of these studies partly replicated their findings in similar cohorts [9,11]. However, no study has yet investigated these associations in other European populations, and potential sex-specific associations remain unclear [9,10]. Furthermore, no study to date has examined how these associations may be influenced by smoking habits. This is relevant since smoking may interact with alcohol in its effect on the risk of diseases such as cancer [12,13,14].

Our objective was to further investigate associations of alcohol consumption with circulating concentrations of metabolites using a large dataset of ~3000 participants of the European Prospective Investigation into Cancer and Nutrition (EPIC) cohort, including participants from 10 European countries. We applied a discovery and replication approach, and explored potential heterogeneity by sex and smoking status.

## 2. Materials and Methods

### 2.1. Study Design and Participants

EPIC is a large multicenter cohort study; its design and methods have been described in detail previously [15,16]. Between 1992 and 2000, approximately 520,000 healthy men and women (mostly 35–70 years of age) were recruited in 23 centers throughout 10 European countries [16]. There is no detailed information available on ethnicity of EPIC subjects. However, based on the ethnic composition of the regions involved in the study at the time of recruitment, the vast majority (>97%) of participants recruited to the EPIC cohort are of Caucasian origin. At recruitment, dietary and lifestyle data were obtained using questionnaires. In addition, blood samples were collected from most participants by each center in a standardized manner [16]. Blood samples were stored at the International Agency for Research on Cancer (IARC, Lyon, France) at −196 °C in liquid nitrogen. The EPIC study was approved by the relevant ethical review committees of each center and by the IARC ethics committee. All study participants provided informed consent. For information on how to submit an application for gaining access to EPIC data and/or biospecimens, please follow the instructions at http://epic.iarc.fr/access/index.php.

This study used data from 2974 control participants from four case-control studies on colorectal (*n* = 491) [17], hepatobiliary (*n* = 327) [18], kidney (*n* = 635) [19], and prostate cancer (*n* = 1521) [20] nested in the EPIC cohort, for which targeted metabolomics data had been acquired. None of the control participants were included in multiple case-control studies (i.e., no duplicates). Figure S1 depicts the flow chart of participant inclusion. These case-control studies will hereafter be referred to as ‘sub-studies’. In the colorectal cancer case-control study, mainly individuals with fasting blood samples (≥6 h) were included. In the hepatobiliary cancer case-control study, individuals from Denmark and Sweden were included, but not in the other sub-studies since blood storage of participants from these countries was not centralized at IARC. For all sub-studies, controls were mostly selected from the full cohort of individuals who were alive and free of cancer (except non-melanoma skin cancer) at the time of diagnosis of the cases, using incidence density sampling and with controls matched to cases by age, sex, study center, follow-up time since blood collection, time of day and fasting status at time of blood collection (<3, 3–6, >6 h). For women, additional matching criteria included menopausal status (premenopausal, postmenopausal, perimenopausal/unknown; this criterion was not applied in the kidney cancer case-control study), phase of menstrual cycle, and hormone replacement therapy use at blood collection. Only control participants were included in this study to avoid any potential bias due to metabolic changes induced by pre-clinical cancer development at the time of recruitment in participants that were subsequently diagnosed with cancer (i.e., cases).

### 2.2. Alcohol Intake and Other Lifestyle Variables

Dietary intake including alcohol consumption was assessed at baseline using validated country-specific or center-specific questionnaires designed to measure dietary consumption in the year preceding its administration. Alcohol consumption at recruitment was calculated in grams per day (g/day) as previously described [21]. Briefly, the frequency of intake of glasses of alcoholic beverages, including wine, fortified wines, beer and cider, spirits and brandy, aniseed drinks and liqueurs, as reported in the questionnaire were converted into g/day of alcohol by applying empirically derived definitions of standard drinks for each beverage and country in EPIC, and summed up to determine intakes of alcohol subtypes. These were further summed up to calculate total alcohol intake through alcoholic beverages. Participants were classified according to their alcohol intake as non-drinkers (<0.1 g/day), light drinkers (0.1–4.9 g/day), moderate drinkers (5.0–39.9 g/day), or heavy drinkers (≥40 g/day).

Other dietary and lifestyle variables were considered in the study. Smoking status and physical activity were assessed through the EPIC lifestyle questionnaire [22]. The Cambridge physical activity index was used, which combines information on occupational activities with recreational activities [23]. Body mass index (BMI; kg/m^2^) was computed from height and weight, measured by trained personnel according to standardized protocols in all centers, except for the majority of the French and Oxford cohorts, where height and weight were self-reported [24].

### 2.3. Alcohol Intake and Other Lifestyle Variables

A targeted metabolomics approach was applied to measure the concentrations of a panel of metabolites in blood (serum for hepatobiliary cancer study, and citrate plasma for all other sub-studies; after two or three freeze–thaw cycles in all cases) at IARC’s laboratory, Lyon, France, using the Absolute *IDQ*^TM^ p180 kit (BIOCRATES Life Sciences AG, Innsbruck, Austria). The assay quantifies up to 186 metabolites, namely acylcarnitines, amino acids, biogenic amines, a sum of hexoses, phosphatidylcholines (PCs) including lysoPCs, diacyl PCs, and acyl–alkyl PCs, and sphingomyelins (SMs). The procedures and metabolite nomenclature have been described in detail previously [25,26]. Briefly, samples were analyzed by ultra-performance liquid chromatography (LC; 1290 Series HPLC; Agilent, Les Ulis, France) coupled to a tandem mass spectrometer (MS/MS; QTrap 5500; AB Sciex, Les Ulis, France, for the hepatobiliary and kidney cancer sub-studies; and Triple Quad 4500; AB Sciex, Framingham, MA, USA, for the colorectal and prostate cancer sub-studies). Amino acids and biogenic amines were quantified by an LC-MS/MS method using appropriately labeled internal standards, whereas flow injection analysis was used for acylcarnitines, hexoses, PCs, and SMs. The samples were analyzed at different time points in each sub-study. In total, there were 89 analytical batches with about 30–39 samples from control participants per batch.

Metabolites with inter-batch or intra-batch coefficients of variation (CVs) > 20% for analytical replicates were excluded from the analysis in all sub-studies, leading to a total of 158 metabolites being detected in the controls of at least one of the sub-studies. Of these, metabolites with >20% of missing values and/or measurements outside the measurable range (i.e., below the limit of detection/quantification or above highest calibration standards) were excluded, resulting in a total of 123 metabolites included in the current analysis, including 10 acylcarnitines, 21 amino acids, four biogenic amines, 75 PCs (eight lysoPCs, 33 diacylPCs, and 34 acyl-alkylPCs), 12 SMs, and a sum of hexoses (Table S1 lists measurement information on all measured metabolites). For the included metabolites, measurements below the limit of detection or quantification (where applicable) were set to half the batch-specific limit of detection or quantification, respectively. For the first assay round of the prostate cancer sub-study (batches 1–31), no limits of detection/quantification were available so these were set to half the lowest measured concentration in that batch. Finally, all measurements above the highest calibration standards were set to the highest standard. Metabolite concentrations (µM) were log-transformed (natural logarithm) as this better approximated a normal distribution for most metabolites, and Z-standardized for better comparison of metabolites with different averages and standard deviations (SDs) of blood concentrations.

A good to excellent reliability for the majority of compounds measured through this method was observed when comparing samples collected in the same individuals over a period of four months [27] and two years [25] (intra-class correlation coefficients > 0.50 for most metabolites).

### 2.4. Statistical Analysis

Descriptive analyses were performed for sociodemographic, lifestyle, and blood sampling-related variables. Summary statistics and Spearman correlation coefficients were computed for metabolites and visualized in heat maps.

The Principal Component Partial *R*-square (PC-PR2) method [28] was applied to estimate the contribution to total variability in metabolite concentrations attributed to self-reported alcohol intake (natural logarithm of continuous alcohol intake + 1) and other factors, including sub-study (categorical), batch (categorical), sex (categorical), age at blood collection (continuous), country (categorical), fasting status at blood collection (categorical: <3 h/3–6 h/>6 h/unknown), smoking status at recruitment (categorical: current/former/never/unknown), BMI (continuous), Cambridge physical activity index (categorical: inactive/moderately inactive/moderately active/active/unknown), and daily intake of energy, meat and meat products, fish and shellfish (all continuous). Firstly, principal component analysis was conducted on metabolite concentrations, and the components explaining > 80% of the total variability were retained (*c* = 18). Then, in multiple linear models the component scores were, in turn, regressed on the list of aforementioned independent variables, and *R*_partial_^2^ for each covariate was estimated separately for each component. Lastly, an overall *R*_partial_^2^ for each covariate was calculated as a weighted average, using the eigenvalues of each principal component c as weights. As a result, the *R*_partial_^2^ provides a measure of the variability in the ensemble of metabolite concentrations that each covariate contributes to explain [28].

To be able to adjust for sex, sub-study, and analytical batch in the main analysis, residuals of each of the Z-standardized ln-transformed metabolite concentrations were computed from linear mixed models with sex as an independent variable and random intercepts for analytical batches nested within studies. The residuals were used as dependent variables in linear regression models testing confounder-adjusted associations with alcohol intake.

The dataset was randomly split into discovery (2/3 of all participants) and replication sets (1/3). Associations of alcohol intake with metabolites were analyzed in the discovery set using multiple linear regression, with false discovery rate (FDR) adjustment of *p*-values, using the Benjamini–Hochberg method (*q*-values < 0.05 were considered significant) [29,30]. Alcohol intake (g/day) was log-transformed (natural logarithm of continuous alcohol intake + 1), to make regression residuals more homoscedastic. Models were adjusted for sex, age at blood collection (continuous), country, fasting status at blood collection (<3 h/3–6 h/>6 h/unknown), smoking status at recruitment (current/former/never/unknown), BMI (continuous), Cambridge physical activity index (inactive/moderately inactive/moderately active/active/unknown), and daily intake of energy, meat and meat products, fish and shellfish (all continuous). Statistically significant metabolites were further evaluated in the replication set, using the same list of confounders as in the discovery analysis. In this phase, the more conservative Bonferroni correction was used (Bonferroni-adjusted *p*-values < 0.05 were considered significant) [31].

#### 2.4.1. Heterogeneity by Sex and Smoking Status

To explore potential heterogeneity by sex and smoking status, interaction terms were tested with FDR-adjustment. In addition, sex-stratified analyses were performed to evaluate potential sex-specific alcohol-related metabolites. In addition, analyses were conducted in non-smokers to rule out the possibility of residual confounding by smoking.

#### 2.4.2. Sensitivity Analyses

Linearity of associations was investigated using penalized spline regression models [32]. Smoothened scatterplots with five knots were generated and goodness-of-fit tests were performed [33]. In addition, analyses in the discovery and replication sets were conducted comparing heavy vs. light drinking (≥40 and 0.1–4.9 g/day, respectively) to possibly identify metabolites associated with extreme alcohol intake. Furthermore, an analysis stratified by sub-study was conducted to investigate the consistency of findings across sub-studies for the metabolites detected as significant in the replication dataset. Similar results were obtained in sensitivity analyses in the discovery set as in the main analysis, including (1) addition of education level as a potential confounder; (2) excluding hepatobiliary controls (serum samples vs. citrate plasma in other studies); (3) excluding non-drinkers, and therefore, these results were not reported.

Linear regression analyses and penalized splines were conducted using Stata [34]. The PC-PR2 analysis, heat maps, and Manhattan plots were computed in R [35].

## 3. Results

### 3.1. Participant Characteristics

General characteristics of the study population (*n* = 2974, 75% men), within the discovery (*n* = 1983) and replication sets (*n* = 991) are shown in Table 1. As expected, the two sets were similar in terms of socio-demographic, lifestyle, and blood-sampling related characteristics, thus confirming the random allocation of study participants. Mean age at recruitment was 58.3 years (SD = 7.7). The majority of participants were overweight (BMI: 25.0–29.9 kg/m^2^; 49.7%) or obese (BMI > 30 kg/m^2^; 17.9%) at recruitment. Most participants were inactive (28.1%) or moderately inactive (33.4%), whilst 21.1% were moderately active and 17.4% active. At baseline over one third of the population reported being never (38.9%) and former smokers (38.5%), while 22.6% indicated that they were current smokers. The median reported alcohol intake at recruitment was 13.7 g/day in men (5th, 95th percentile: 0.0, 64.0) and 2.8 g/day in women (0.0, 25.7). A total of 345 participants were classified as non-drinkers (11.6%; 48.7% men), 726 as light drinkers (24.4%; 62.7% men), 1521 as moderate drinkers (51.1%; 81.8% men), and 382 as heavy drinkers (12.8%; 95.3% men). Participant characteristics by sub-study were compared in Table S2, indicating similar study populations overall.

### 3.2. Metabolome Characteristics and PC-PR2

The summary statistics of metabolite concentrations are reported in Table S3, and median Spearman correlation coefficients among the 123 metabolites are shown in Figure S2. Within classes of metabolites, median correlations among SMs and lysoPCs were 0.83 (5th, 95th percentile: 0.50, 0.92) and 0.62 (0.44, 0.76), respectively, while lower values were found for acyl-alkyl PCs (0.43; 0.12, 0.74), diacyl PCs (0.34; −0.10, 0.69), amino acids (0.31; 0.09, 0.62), acylcarnitines (0.28; 0.03, 0.61), and biogenic amines (0.22; 0.11, 0.36). 

**Figure 1:**
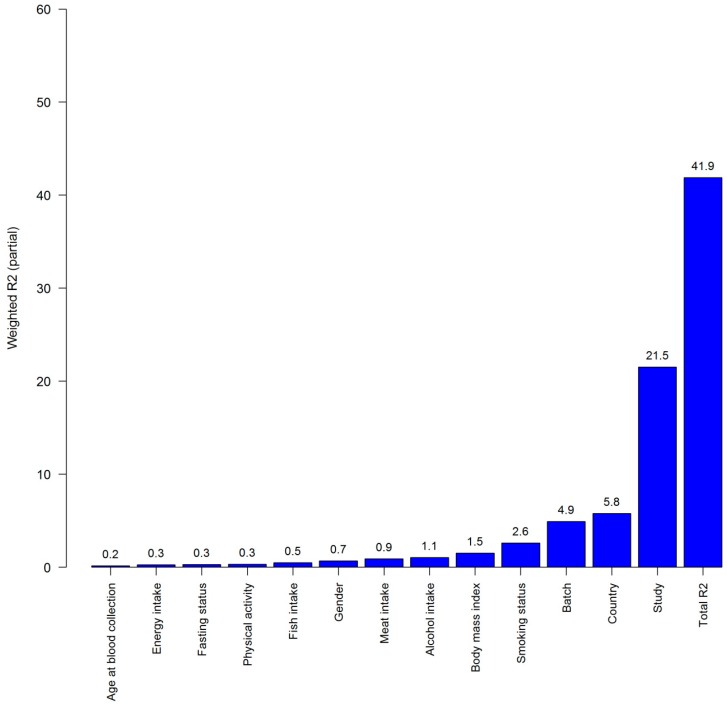
Overall *R*_partial_^2^ and weighted *R*_partial_^2^ for each covariate (sub-study, lifestyle and laboratory variables), indicating the percentage explained variability in metabolite concentrations. Alcohol was included as a log-transformed variable (natural logarithm of continuous alcohol intake + 1) as in the main multivariable analysis.

Results of PC-PR2 analysis indicated that sub-study, lifestyle, and laboratory variables combined explained 41.9% of the total variability in metabolite concentrations (Figure 1). The main contributors to variability were sub-study (21.5%), country (5.8%), and batch (4.9%), while alcohol intake explained 1.1%. Sex and smoking status explained 0.7% and 2.6% of the total variability, respectively. Additional covariates such as education level, macronutrient intake (fat, carbohydrates and protein), and time between blood collection and metabolomics assay showed marginal percentages of explained variability in the PC-PR2 analysis and were not included in the final analysis.

### 3.3. Associations of Alcohol with Metabolites

**Figure 2:**
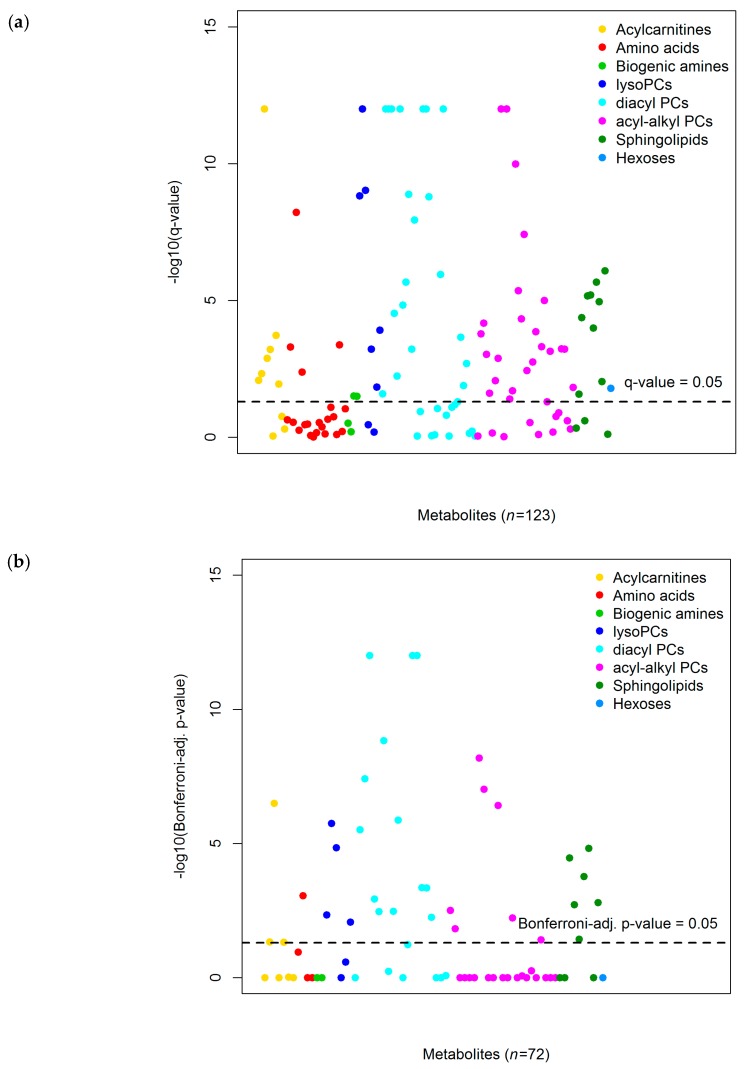
Manhattan plots showing −log_10_ of FDR *q*-values and Bonferroni-adjusted *p*-values of associations of alcohol intake with metabolites in the (**a**) discovery (false discovery rate method; *n* = 1983) and (**b**) replication analysis (Bonferroni correction; *n* = 991), respectively. Footnote: Analyzed with multivariable linear regression analyses analyzing associations of alcohol consumption (ln-transformed alcohol intake + 1) as main independent variable and as dependent variables the residuals obtained from linear mixed models with Z-standardized ln-transformed metabolite concentrations as dependent variables, sex as independent variable, and random intercepts for analytical batches nested within studies. Adjusted for: sex; age (y; continuous), body mass index (kg/m^2^; continuous), self-reported physical activity levels (Cambridge physical activity index [23]: inactive, moderately inactive, moderately active, active, unknown), fasting status (≥6 h, 3–5.9 h, <3 h, unknown), meat intake (g/day; continuous), fish intake (g/day; continuous), energy intake (kcal/day; continuous), country, and smoking status (current, former, never, unknown). The discovery and replication set were taken as random samples without replacement of 66.7% and 33.3% of the total dataset, respectively. Q-values/Bonferroni-adjusted *p*-values <1.0 × 10^−12^ (number of decimals above reporting limits of STATA and thus not provided) were set to 1.0 × 10^−12^.

In the discovery set, alcohol intake was found to be significantly associated with concentrations of 72 out of 123 metabolites, after FDR-adjustment, as shown in Figure 2a and in Table S4. In the replication phase, 34 of these 72 metabolites (47.2%) were significantly related to alcohol intake, after Bonferroni correction, as displayed in Figure 2b and in Table 2 (Table S4 with full results for all tested metabolites). In particular, significant associations were observed of alcohol intake with several lipid metabolites, including four lysoPCs, 13 diacyl PCs, seven acyl-alkyl PCs, and six SMs. In addition, we observed associations with three acylcarnitines and the amino acid citrulline. Associations with acylcarnitines and phosphatidylcholines were generally positive, while mostly inverse associations were observed with citrulline and SMs. The three strongest associations were with PC aa C32:1 (regression coefficient: 0.22; standard error: 0.02), PC aa C36:5 (0.17; 0.02), and PC aa C36:4 (0.15; 0.02) (all Bonferroni-adjusted *p*-value < 1.0 × 10^−12^). The directions of significant associations were similar, as observed in the discovery analysis.

### 3.4. Heterogeneity by Sex and Smoking Status

Significant interactions by sex and smoking status were found for 11 and three metabolites, respectively, in the discovery set after FDR adjustment, as shown in Tables S5 and S6.

In the analysis stratified by sex, a total of 68 and seven metabolites were found to be statistically significant after FDR-adjustment in men (*n* = 1378) and women (*n* = 378), respectively (Table S5). An analysis of the overlap (Figure S3) indicated that the majority of metabolites identified in sex-specific models had also been found in the main analysis, consistently in men (63 out of 68) and women (seven out of seven).

Analysis in never smokers only revealed that 31 metabolites were significantly related to alcohol intake, of which 29 metabolites were previously identified in the main analysis, mostly with the same directions and overall similar magnitude of associations (Table S6).

### 3.5. Sensitivity Analyses

Visual inspection of penalized spline regression models of metabolite concentrations against ln-transformed alcohol did not provide strong evidence for non-linear associations of alcohol intake with metabolite concentrations (Figure S4).

In addition, the analysis comparing heavy (>40 g/day of alcohol) vs. light (0.1–4.9 g/day) drinking gave similar results as the main analysis (Table S7). The majority of significant metabolites (86.2%) had also been identified in the main analysis (Figure S5). Four metabolites were significantly related to extreme alcohol consumption but not to alcohol as continuous variable, i.e., the amino acid serine, lysoPC a C20:4, PC aa C40:4, and PC ae C40:6.

The regression coefficients obtained from analysis stratified by sub-study, for the 34 metabolites detected as significant in the replication dataset, indicated a satisfactory level of consistency of estimates across sub-studies (Figure S6).

## 4. Discussion

In this study, which is one of the largest to explore the associations between circulating metabolites and alcohol consumption, alcohol intake was related to several phospho- and sphingolipids. In particular, we observed associations with circulating concentrations of four lysoPCs, 13 diacyl PCs, seven acyl-alkyl PCs, and six SMs. In addition, alcohol was related to three acylcarnitines and the amino acid citrulline.

**Figure 3:**
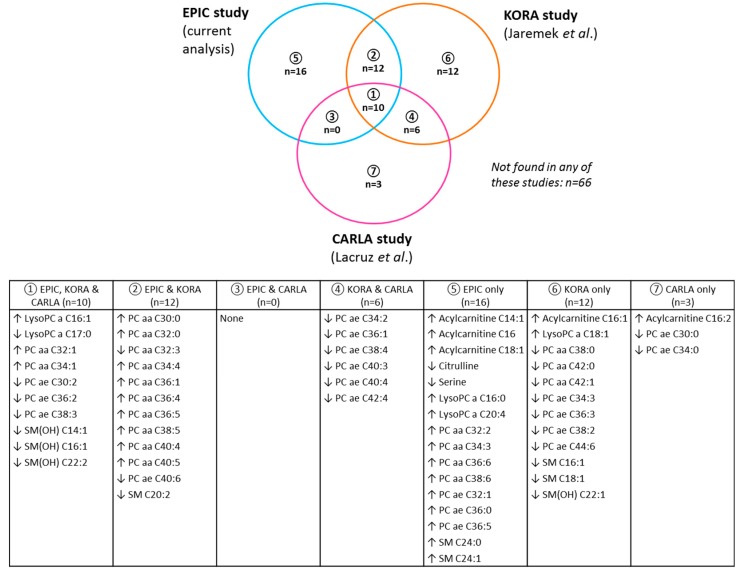
Venn diagram showing overlap in metabolites identified as significantly associated with alcohol intake after discovery and replication analysis in the current study (EPIC) and those identified by two other previous German population-based studies that were described by Jaremek et al. [9] (KORA study; sex-stratified analysis in 1144 men and 946 women) and Lacruz et al. [10] (CARLA study; combined analysis in 534 men and 496 women), that applied the previous version of the assay used in the current analysis (i.e., BIOCRATES AbsoluteIDQTM p150 kit; p180 kit was used in the current analysis). An upwards arrow indicates a positive association (i.e., higher alcohol intake associated with higher blood concentrations of the metabolite), while a downwards arrow indicates a negative association (i.e., higher alcohol intake associated with lower metabolite concentrations). Footnote: The total number of metabolites (*n* = 125) is more than those included in the current analysis (*n* = 123) as two metabolites measured in the KORA and/or CARLA study were not included in the current analysis (acylcarnitine C16:1 and acylcarnitine C16:2; see Table S1). For an explanation of abbreviated metabolite names, see Table S1.

Of the 38 replicated metabolites associated with alcohol in our study, 10 metabolites were similarly linked to alcohol in two previous studies in German populations, namely the KORA study, conducted in 1144 men and 946 women [9], and the CARLA study, a combined analysis of 534 men and 496 women [10] (Figure 3). Another 12 of these 38 metabolites were observed in the KORA study [9] only. The metabolites overlapping with these studies included lysoPC a C16:1 and lysoPC a C17:0, and several diacyl PCs, acyl-alkyl PCs, and SMs. Two of the overlapping diacyl PCs have also been associated with wine intake in a study including over 3500 female twins from the United Kingdom, i.e., PC aa C32:1 and PC aa C36:5 [11].

These findings suggest potential biological disrupting conditions related to alcohol consumption [9,10]. Lower SM concentrations in individuals with higher alcohol consumption may have resulted from alcohol-induced activation of the enzyme acid sphingomyelinase (ASM), which leads to increased catabolism of SMs into ceramide and PCs [36,37]. This could lead to hepatotoxicity [38,39]. Low acyl–alkyl PCs and high diacyl PCs may reflect less lipid remodeling in membranes resulting in inflammation [40], which may, in turn, be associated with alcohol-related pathologies [41]. High lysoPCs may be the result of alcohol-induced stimulation of several metabolic pathways [9,10]. LysoPCs have been associated with increased levels of oxidative stress [10], that has been related to alcohol-induced liver diseases [42]. The association of alcohol intake with blood concentrations of lysoPC a C17:0 may reflect negative confounding by dairy consumption among people with high alcohol intake [9,10], as the fatty acid C17:0 is specifically found in milk fat and cannot be synthesized in the human body [43]. However, a significant inverse association was still observed when we additionally adjusted the analysis of alcohol with lysoPC a C17:0 for dairy consumption (results not shown).

We also observed associations with metabolites that had not been identified before, including positive associations with three acylcarnitines and inverse associations with the amino acids citrulline and serine (the latter only with heavy vs. light drinking). Acylcarnitines are related to mitochondrial oxidation of fatty acids and are biomarkers of mitochondrial dysfunction [44]. Higher plasma concentrations of acylcarnitines have been observed in patients with alcohol-induced cirrhosis in comparison to healthy controls [45,46]. This dysregulation of carnitine metabolism may be caused by an increased carnitine biosynthesis induced by increased muscle protein turnover in cirrhotic patients [45]. Citrulline is a key intermediate in the urea cycle [47], while serine is a proteinogenic amino acid and a precursor of other important metabolites including sphingolipids and nucleotides [48]. Lower concentrations of these amino acids observed in participants with higher alcohol intake may reflect a dysregulation of these metabolic pathways. Previous population-based studies did not find associations of alcohol intake with serine, whilst citrulline was not measured in these studies [9,10,11]. Further research will be necessary to replicate our findings, and investigate whether these metabolites relate to alcohol-related diseases.

Similarly to the CARLA study [10], we did not observe strong heterogeneity of alcohol-metabolite associations by sex as significant interactions were found for 11 metabolites only. In sex-specific analyses in the KORA study, only a subset of the metabolites that were significantly related to alcohol intake in men were also observed in women [9], possibly reflecting a lower sample size and lower alcohol intake in women compared to men. We did not find strong evidence of heterogeneity in associations between alcohol consumption and metabolite concentrations by smoking status, with three statistically significant interactions only. Several metabolites identified in the overall analysis were not related to alcohol in never smokers, which may suggest residual confounding, or may be due to the lower sample size among never smokers.

An important strength of our study is the large sample size including controls from four nested case-control studies within EPIC, encompassing wide variability in alcohol intake and metabolite concentrations. The comprehensive evaluation of the contribution of lifestyle and laboratory variables to overall metabolite variability through the PC-PR2 analysis enabled proper adjustment in our evaluation. An important element of our study was the use of discovery and replication sets that increased the reliability of our findings [49]. The metabolomics analyses were undertaken in one laboratory, thus avoiding inter-laboratory variability [26]. Overall, the consistency of our results with findings in previous studies using the same assay [9,10], and the associations with metabolites involved in lipid pathways in observational studies with different assay methods [50,51,52] suggests that our findings are robust.

Among the limitations of our study, alcohol intake at recruitment was self-reported, which may be prone to misreporting [53], although alcohol measurements in EPIC were validated against multiple 24-h recalls with Spearman correlation of 0.79 [54]. Our analysis was cross-sectional, limiting the possibility of assessing causality. Results of the PC-PR2 analysis showed that the factor ‘sub-study’ explained a relatively large percentage of variability in metabolite concentrations, likely due to analytical differences, e.g., time of acquirement and mass spectrometer used, and population characteristics. Proper adjustment for sub-study was carried out in our analysis using the residual method. Furthermore, results of the sensitivity analysis stratified by sub-study indicated a satisfactory level of consistency across sub-studies. In the hepatobiliary cancer case-control study serum samples were analyzed, while in other studies citrate plasma was used. Although absolute concentrations of metabolites in the two matrices may differ, high correlation between serum and plasma measurements have been observed for the majority of metabolites in the BIOCRATES assay with a mean Pearson correlation of 0.81 [55], and a good reliability for most metabolites in both serum and plasma has been found [55,56]. Furthermore, there were no differences in results when we excluded hepatobiliary cancer controls. Our samples included both fasting and non-fasting samples, but the PC-PR2 analysis conducted in our study showed limited impact of fasting on variability of metabolite concentrations and we adjusted for fasting status in our analysis. Furthermore, it has been found within the EPIC study that the majority of metabolites of the BIOCRATES kit were reliably measured in fasting and non-fasting samples, although a lower stability for certain acylcarnitines, PCs, and SMs was found for non-fasting samples [25]. 

## 5. Conclusions

In conclusion, findings from this large European study confirm that alcohol intake was associated with circulating concentrations of several phospholipids and sphingolipids, and provide novel evidence of a relationship with concentrations of acylcarnitines and specific amino acids. More research will be necessary to replicate these findings in other study populations. The availability of longitudinal data would clarify whether alcohol intake could modify specific metabolic responses within the same individuals over time.

## Figures and Tables

**Table 1:** Socio-demographic, lifestyle, and blood-sampling related characteristics of participants included in the total dataset and in the discovery and replication sets ^a^.

	Discovery Set (*n* = 1983, 66.7%)		Replication Set (*n* = 991, 33.3%)		Total Dataset (*n* = 2974, 100%)	
Age, *mean* (*SD*)	58.5	(7.6)	58.0	(7.9)	58.3	(7.7)
Sex, *n* (%)						
Men	1497	(75.5)	734	(74.1)	2231	(75.0)
Women	486	(24.5)	257	(25.9)	743	(25.0)
Education level, *n* (%) ^b^						
None/primary	891	(46.8)	408	(43.1)	1299	(45.5)
Secondary	236	(12.4)	117	(12.4)	353	(12.4)
Technical/professional	410	(21.5)	239	(25.2)	649	(22.8)
University or higher	369	(19.4)	183	(19.3)	552	(19.4)
Alcohol intake in men (g/day), *median* (*5th*, *95th perc*)	14.0	(0.0, 67.9)	14.0	(0.0, 67.9)	13.7	(0.0, 64.0)
Alcohol intake in women (g/day), *median* (*5th*, *95th perc*)	3.2	(0.0, 26.4)	2.2	(0.0, 25.7)	2.8	(0.0, 25.7)
Categories of alcohol intake, *n* (%)						
Non-drinkers (<0.1 g/day)	227	(11.5)	118	(11.9)	345	(11.6)
Light drinkers (0.1–4.9 g/day)	485	(24.5)	241	(24.3)	726	(24.4)
Moderate drinkers (5–39.9 g/day)	1026	(51.7)	495	(50.0)	1521	(51.1)
Heavy drinkers (≥40 g/day)	245	(12.4)	137	(13.8)	382	(12.8)
Body mass index (kg/m^2^), *mean* (*SD*)	26.8	(3.8)	26.9	(3.6)	26.8	(3.7)
Physical activity, *n* (%) ^c^						
Inactive	542	(27.7)	281	(28.7)	823	(28.1)
Moderately inactive	652	(33.4)	328	(33.5)	980	(33.4)
Moderately active	406	(20.8)	214	(21.9)	620	(21.1)
Active	354	(18.1)	156	(15.9)	510	(17.4)
Smoking status, *n* (%) ^d^						
Current smoker	445	(22.7)	219	(22.6)	664	(22.7)
Former smoker	764	(38.9)	365	(37.7)	1129	(38.5)
Never smoker	754	(38.4)	385	(39.7)	1139	(38.9)
Meat intake (g/day), *median* (*5th*, *95th perc*)	106.1	(34.1, 220.2)	108.4	(31.2, 233.0)	106.6	(32.9, 222.9)
Fish intake (g/day), *median* (*5th*, *95th perc*)	28.8	(2.5, 113.3)	28.8	(3.0, 113.0)	28.8	(2.5, 113.2)
Energy intake (kcal/day), *median* (*5th*, *9th perc*)	2187.4	(1330.0, 3480.4)	2190.5	(1304.9, 3451.3)	2188.3	(1327.7, 3480.4)
Fasting status, *n* (%) ^e^						
≥6 h	775	(40.2)	391	(40.4)	1166	(40.2)
3–5.9 h	371	(19.2)	193	(19.9)	564	(19.5)
<3 h	784	(40.6)	385	(39.7)	1169	(40.3)
Sub-study, *n* (%)						
Colorectal cancer controls	334	(16.8)	157	(15.8)	491	(16.5)
Kidney cancer controls	401	(20.2)	234	(23.6)	635	(21.4)
Hepatobiliary cancer controls	209	(10.5)	118	(11.9)	327	(11.0)
Prostate cancer controls	1039	(52.4)	482	(48.6)	1521	(51.1)

Abbreviations: n, number; perc, percentile; SD, standard deviation. ^a^ The discovery and replication set were taken as random samples without replacement of 66.7% and 33.3% of the total dataset, respectively. ^b^ Data missing for 121 participants (77 from discovery set (3.9%) and 44 from replication set (4.4%)). ^c^ Cambridge physical activity index: cross-classification of the level of occupational activity with cycling and sports activities and recreational activities [23]; data missing for 41 participants (29 from discovery set (1.5%) and 12 from replication set (1.2)). ^d^ Data missing for 42 participants (20 from discovery set (1.0%) and 22 from replication set (2.2%)). ^e^ Data missing for 75 participants (53 from discovery set (2.7) and 22 from replication set (2.2)).

**Table 2:** Results of discovery and replication analysis of metabolites that were significantly associated with alcohol consumption in the discovery and replication set ^a^.

	Discovery Analysis (*n* = 1983) ^b^				Replication Analysis (*n* = 991) ^b^			
Metabolite	β	(SE)	*p*-Value	(FDR *q*-Value) ^c^	β	(SE)	*p*-Value	(Bonf.-adj. *p*-Value) ^d^
Acylcarnitine C14:1	0.05	(0.02)	2.2 × 10^−3^ 2.2 × 10^−3^	(4.7 × 10^−3^)	0.08	(0.02)	6.5 × 10^−4^	(4.7 × 10^−2^)
Acylcarnitine C16	0.11	(0.02)	<1.0 × 10^−12^	(<1.0 × 10^−12^)	0.13	(0.02)	4.4 × 10^−9^	(3.2 × 10^−7^)
Acylcarnitine C18:1	0.06	(0.02)	2.4 × 10^−4^	(6.2 × 10^−4^)	0.08	(0.02)	6.7 × 10^−4^	(4.8 × 10^−2^)
Citrulline	−0.10	(0.02)	8.2 × 10^−10^	(5.9 × 10^−9^)	−0.10	(0.02)	1.2 × 10^−5^	(8.9 × 10^−4^)
LysoPC a C16:0	0.09	(0.01)	1.8 × 10^−10^	(1.5 × 10^−9^)	0.08	(0.02)	6.4 × 10^−5^	(4.6 × 10^−3^)
LysoPC a C16:1	0.11	(0.02)	<1.0 × 10^−12^	(<1.0 × 10^−12^)	0.12	(0.02)	2.5 × 10^−8^	(1.8 × 10^−6^)
LysoPC a C17:0	−0.09	(0.01)	1.0 × 10^−10^	(9.5 × 10^−10^)	−0.11	(0.02)	2.0 × 10^−7^	(1.4 × 10^−5^)
LysoPC a C20:4	0.06	(0.02)	3.5 × 10^−5^	(1.2 × 10^−4^)	0.08	(0.02)	1.2 × 10^−4^	(8.5 × 10^−3^)
PC aa C30:0	0.10	(0.01)	<1.0 × 10^−12^	(<1.0 × 10^−12^)	0.12	(0.02)	4.3 × 10^−8^	(3.1 × 10^−6^)
PC aa C32:0	0.12	(0.01)	<1.0 × 10^−12^	(<1.0 × 10^−12^)	0.13	(0.02)	5.3 × 10^−10^	(3.8 × 10^−8^)
PC aa C32:1	0.20	(0.02)	<1.0 × 10^−12^	(<1.0 × 10^−12^)	0.22	(0.02)	<1.0 × 10^−12^	(<1.0 × 10^−12^)
PC aa C32:2	0.07	(0.02)	7.3 × 10^−6^	(3.0 × 10^−5^)	0.10	(0.02)	1.6 × 10^−5^	(1.2 × 10^−3^)
PC aa C32:3	−0.04	(0.01)	2.7 × 10^−3^	(5.8 × 10^−3^)	−0.08	(0.02)	4.8 × 10^−5^	(3.5 × 10^−3^)
PC aa C34:1	0.17	(0.01)	<1.0 × 10^−12^	(<1.0 × 10^−12^)	0.14	(0.02)	2.0 × 10^−11^	(1.4 × 10^−9^)
PC aa C34:3	0.07	(0.01)	4.0 × 10^−7^	(2.1 × 10^−6^)	0.08	(0.02)	4.7 × 10^−5^	(3.3 × 10^−3^)
PC aa C34:4	0.11	(0.02)	1.5 × 10^−10^	(1.3 × 10^−9^)	0.13	(0.02)	1.9 × 10^−8^	(1.3 × 10^−6^)
PC aa C36:4	0.14	(0.02)	<1.0 × 10^−12^	(<1.0 × 10^−12^)	0.15	(0.02)	<1.0 × 10^−12^	(<1.0 × 10^−12^)
PC aa C36:5	0.16	(0.02)	<1.0 × 10^−12^	(<1.0 × 10^−12^)	0.17	(0.02)	<1.0 × 10^−12^	(<1.0 × 10^−12^)
PC aa C36:6	0.11	(0.02)	2.1 × 10^−10^	(1.6 × 10^−9^)	0.10	(0.02)	6.2 × 10^−6^	(4.4 × 10^−4^)
PC aa C38:5	0.09	(0.02)	1.9 × 10^−7^	(1.1 × 10^−6^)	0.11	(0.02)	6.3 × 10^−6^	(4.6 × 10^−4^)
PC aa C38:6	0.12	(0.02)	<1.0 × 10^−12^	(<1.0 × 10^−12^)	0.09	(0.02)	7.8 × 10^−5^	(5.6 × 10^−3^)
PC ae C30:2	−0.05	(0.01)	5.0 × 10^−5^	(1.7 × 10^−4^)	−0.07	(0.02)	4.4 × 10^−5^	(3.1 × 10^−3^)
PC ae C32:1	0.06	(0.02)	1.8 × 10^−5^	(6.8 × 10^−5^)	0.08	(0.02)	2.1 × 10^−4^	(1.5 × 10^−2^)
PC ae C36:0	0.16	(0.02)	<1.0 × 10^−12^	(<1.0 × 10^−12^)	0.15	(0.02)	9.0 × 10^−11^	(6.5 × 10^−9^)
PC ae C36:2	−0.11	(0.02)	<1.0 × 10^−12^	(<1.0 × 10^−12^)	−0.14	(0.02)	1.3 × 10^−9^	(9.5 × 10^−8^)
PC ae C36:5	0.10	(0.02)	1.0 × 10^−11^	(1.0 × 10^−10^)	0.13	(0.02)	5.3 × 10^−9^	(3.8 × 10^−7^)
PC ae C38:3	−0.09	(0.02)	5.9 × 10^−9^	(3.8 × 10^−8^)	−0.09	(0.02)	8.2 × 10^−5^	(5.9 × 10^−3^)
PC ae C40:6	−0.06	(0.02)	2.8 × 10^−4^	(7.3 × 10^−4^)	−0.08	(0.02)	5.4 × 10^−4^	(3.9 × 10^−2^)
SM C20:2	−0.06	(0.01)	1.4 × 10^−6^	(6.8 × 10^−6^)	−0.08	(0.02)	4.8 × 10^−7^	(3.5 × 10^−5^)
SM C24:0	0.04	(0.01)	1.3 × 10^−6^	(6.3 × 10^−6^)	0.05	(0.01)	2.7 × 10^−5^	(1.9 × 10^−3^)
SM C24:1	0.03	(0.01)	2.8 × 10^−5^	(1.0 × 10^−4^)	0.04	(0.01)	5.2 × 10^−4^	(3.8 × 10^−3^)
SM(OH) C14:1	−0.06	(0.01)	3.9 × 10^−7^	(2.1 × 10^−6^)	−0.08	(0.02)	2.4 × 10^−6^	(1.7 × 10^−4^)
SM(OH) C16:1	−0.05	(0.01)	2.6 × 10^−6^	(1.1 × 10^−5^)	−0.08	(0.02)	2.1 × 10^−7^	(1.5 × 10^−5^)
SM(OH) C22:2	−0.05	(0.01)	1.3 × 10^−7^	(8.3 × 10^−7^)	−0.05	(0.01)	2.3 × 10^−5^	(1.6 × 10^−3^)

Abbreviations: β, unstandardized regression coefficient derived from multivariable linear models; Bonf.-adj. *p*-value, Bonferroni-adjusted *p*-value; SE, standard error. For an explanation of abbreviated metabolite names, see Table S1. ^a^ Analyzed with multivariable linear regression analyses analyzing associations of alcohol consumption (ln-transformed alcohol intake + 1) as main independent variable and as dependent variables the residuals obtained from linear mixed models with Z-standardized ln-transformed metabolite concentrations as dependent variables, sex as independent variable, and random intercepts for analytical batches nested within studies. Adjusted for: sex; age (y; continuous), body mass index (kg/m^2^; continuous), self-reported physical activity levels (Cambridge physical activity index [23]: inactive, moderately inactive, moderately active, active, unknown), fasting status (≥6 h, 3–5.9 h, <3 h, unknown), meat intake (g/day; continuous), fish intake (g/day; continuous), energy intake (kcal/day; continuous), country, and smoking status (current, former, never, unknown). ^b^ The discovery and replication set were taken as random samples without replacement of 66.7% and 33.3% of the total dataset, respectively. ^c^ The analysis in the discovery set was adjusted for multiple testing using the false discovery rate (FDR) method. ^d^ The analysis in the replication set was adjusted for multiple testing using Bonferroni correction.

## Supplementary Materials

The following are available online at http://www.mdpi.com/2072-6643/10/5/654/s1, Figure S1: Flow chart of participant inclusion into the European Prospective Investigation into Cancer and Nutrition (EPIC) study and into the current analysis, Figure S2: Heat map showing median Spearman correlation coefficients among the concentrations of metabolites (µM) included in the current analysis (*n* = 123), Figure S3: Venn diagram showing overlap in metabolites identified as being statistically significantly (FDR-adjusted *q*-value < 0.05) associated with alcohol intake (ln-transformed) in the total discovery set, and in subgroups of men and women (stratified analyses), Figure S4: Smoothened scatterplots obtained from penalized spline regression of the metabolite outcome variables against ln-transformed alcohol and covariates (same as in main analysis), Figure S5: Venn diagram showing overlap in metabolites identified as being statistically significantly (Bonferroni-adjusted *p*-value < 0.05) associated with continuous ln-transformed alcohol intake (main analysis) and with heavy drinking (≥40 g/day of alcohol) vs. light drinking (0.1–4.9 g/day of alcohol) in replication analysis, Figure S6: Regression coefficients obtained from analysis of associations of alcohol intake with metabolites in the discovery dataset, stratified by sub-study, i.e., colorectal cancer (CRC), kidney cancer (KC), hepatobiliary cancer (HC), and prostate cancer (PC) case-control study. Table S1: Information on measurements of metabolites included in the BIOCRATES AbsoluteIDQ™ p180 kit in the total dataset and in each sub-study, and whether the metabolite was included for the current analysis, Table S2: Participant characteristics by sub-study (nested case-control study the controls were derived from) and in the total dataset, Table S3: Summary statistics of untransformed concentrations (µM) of metabolites included in the current analysis (*n* = 123), Table S4: Results of discovery and replication analysis on associations of alcohol intake with concentrations of metabolites included in the current analysis (*n* = 123), Table S5: Comparison of results of sex-stratified discovery analysis on associations of alcohol intake with concentrations of included metabolites (*n* = 123) with results obtained in the total discovery set and results of statistical interaction analysis for sex, Table S6: Comparison of results of discovery analysis on associations of alcohol intake with concentrations of included metabolites (*n* = 123) in never smokers only with results obtained in the total discovery set and results of statistical interaction analysis for smoking status, Table S7: Results of discovery and replication analysis on associations of categories of alcohol intake (heavy vs. light) with concentrations of metabolites included in the current analysis (*n* = 123).
