# Supplementary material for: Circulating Metabolites Associated with Alcohol Intake in the European Prospective Investigation into Cancer and Nutrition Cohort
Source: Nutrients. 2018 May 22;10(5):654. doi: 10.3390/nu10050654 (PMC5986533; doi:10.3390/nu10050654)
Supplement: Supplementary file 1 [file nutrients-10-00654-s001.zip › Supplementary Figures.pdf]

Supplementary Figure 1. Flow chart of participant inclusion into the European Prospective Investigation into Cancer and Nutrition (EPIC) study and into the current analysis.

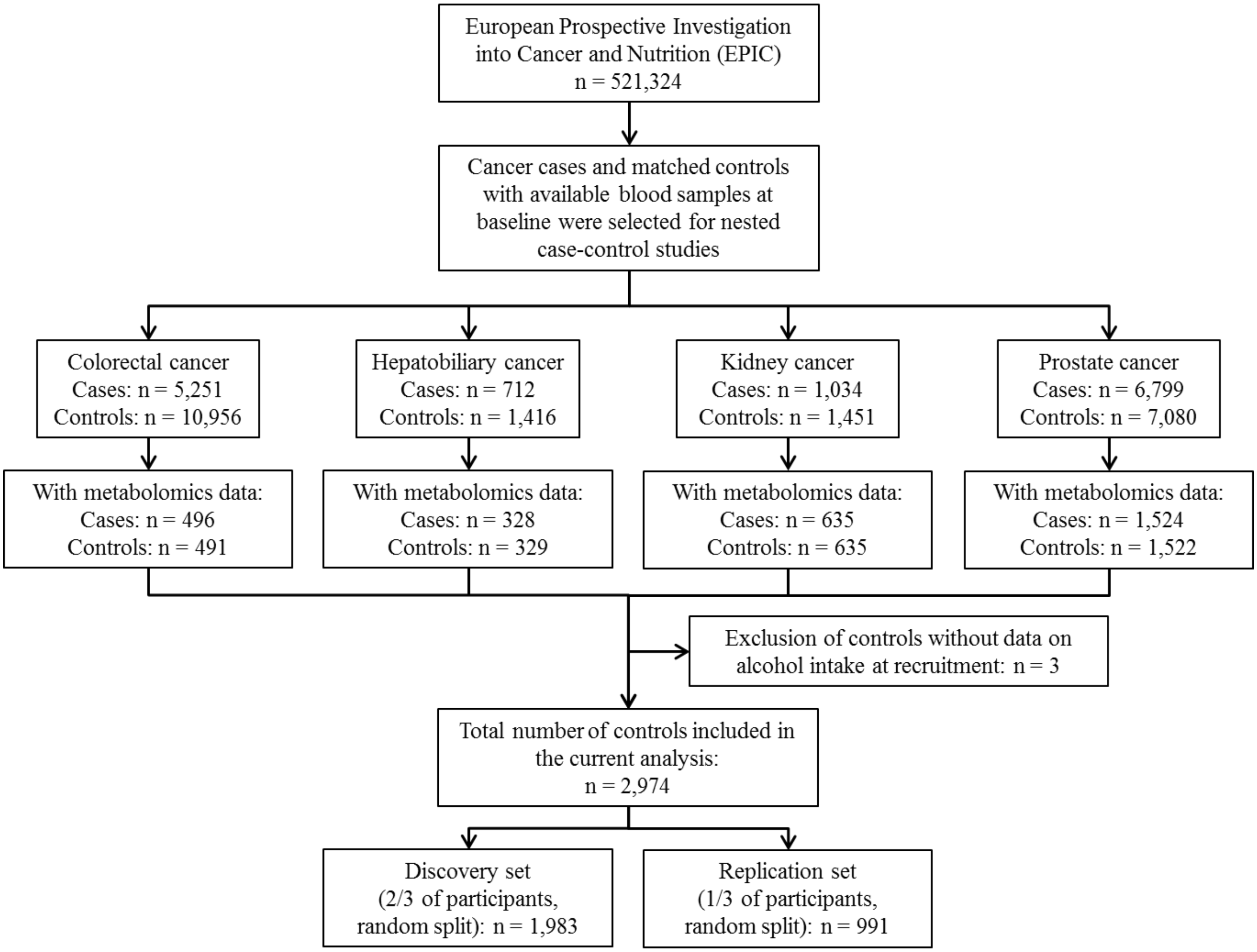

Supplementary Figure 2. Heat map showing median Spearman correlation coefficients among the concentrations of metabolites (μM) included in the current analysis (n=123).

See also submitted TIFF.file: Supplementary Figure 2

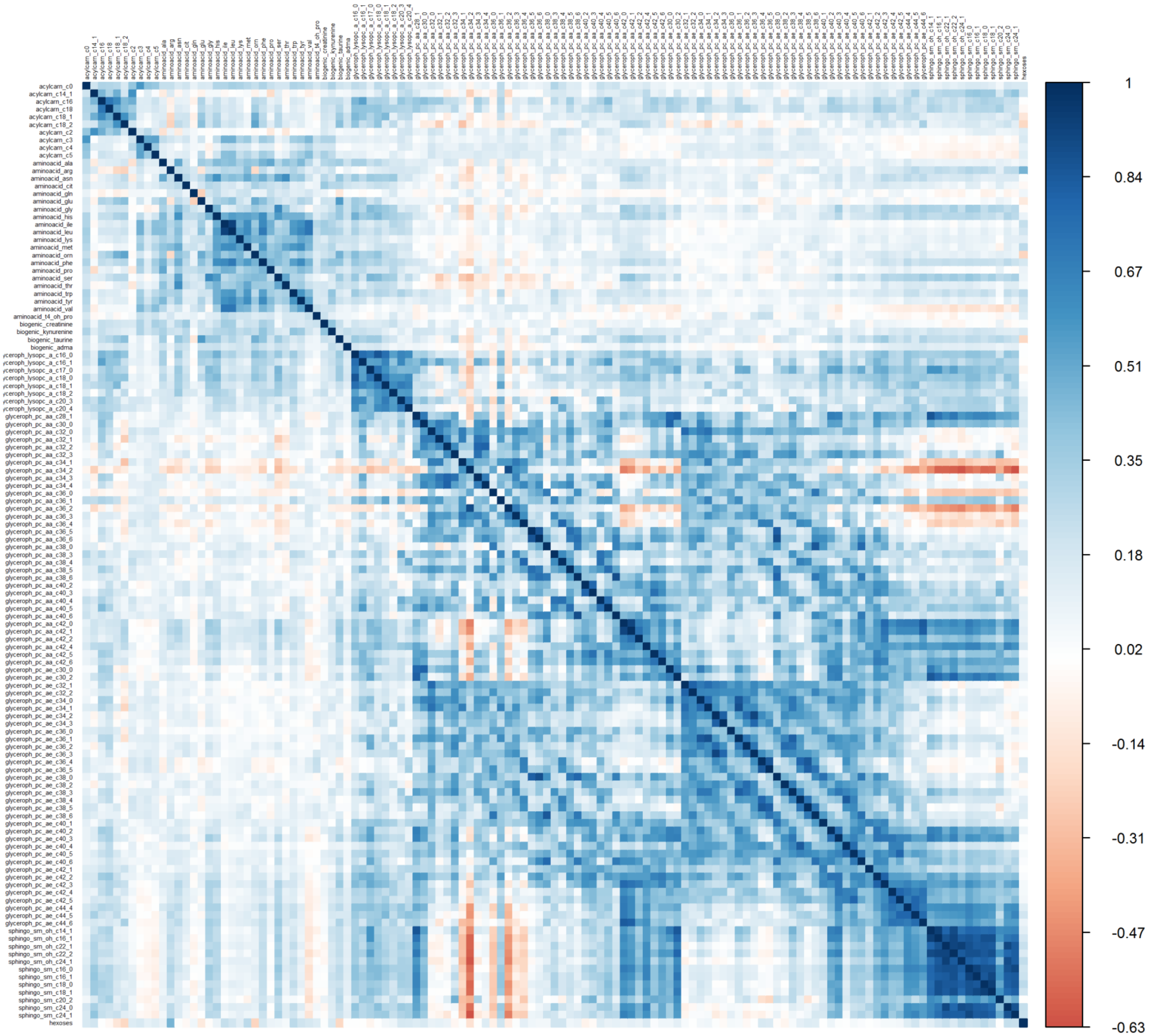

Supplementary Figure 3. Venn diagram showing overlap in metabolites identified as being statistically significantly (FDR-adjusted q-value<0.05) associated with alcohol intake (ln-transformed) in the total discovery set, and in subgroups of men and women (stratified analyses).

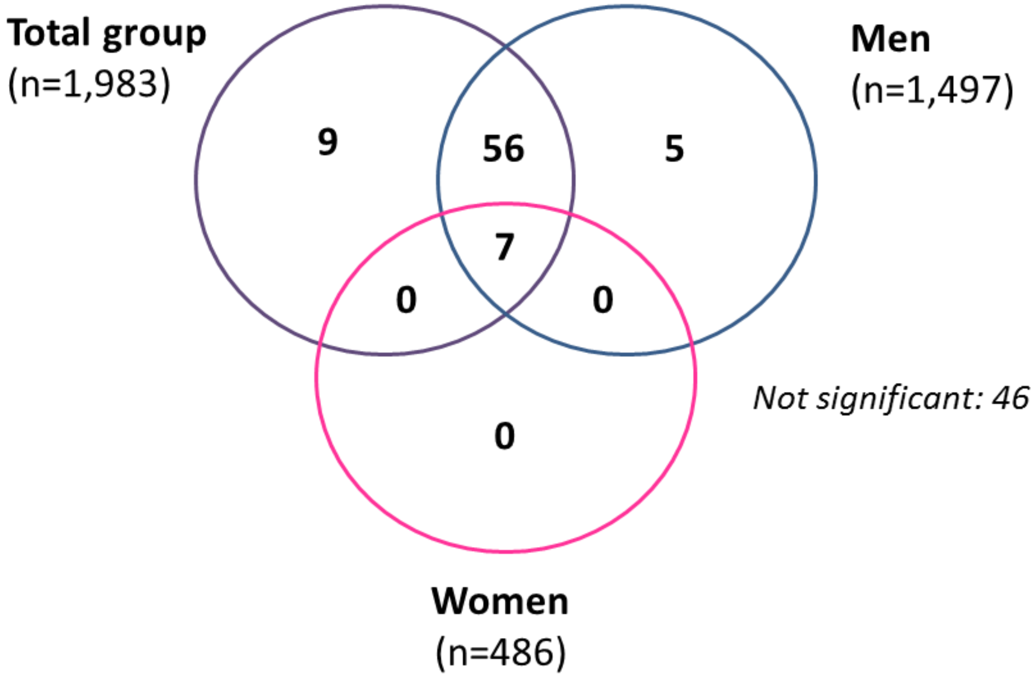

Supplementary Figure 4. Smoothened scatterplots obtained from penalized spline regression of the metabolite outcome variables against ln-transformed alcohol and covariates (same as in main analysis). Scatterplots were generated using 5 knots and goodness-of-fit tests were performed, of which P-values are reported below the plots (lower p-value suggests more evidence against parametric model).

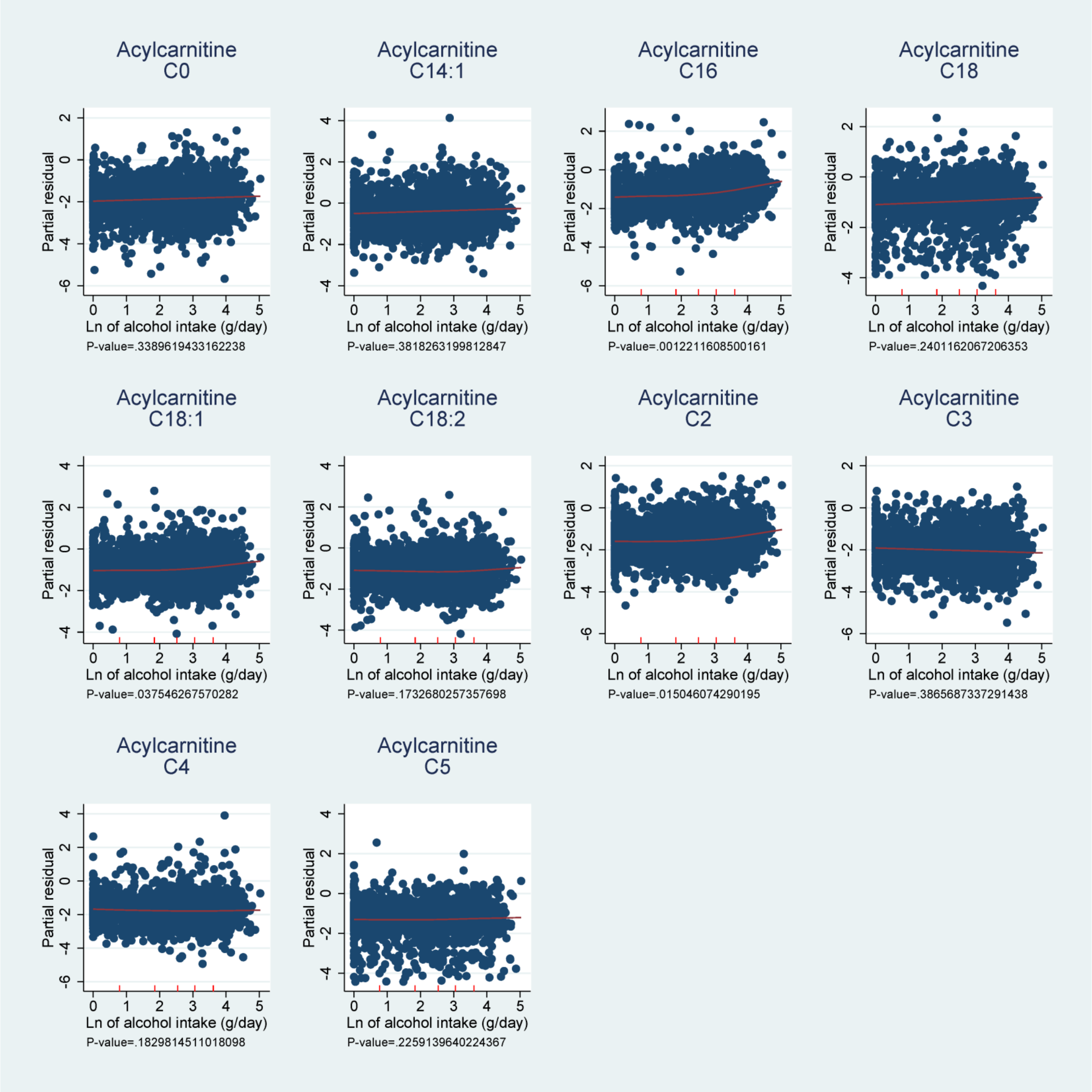

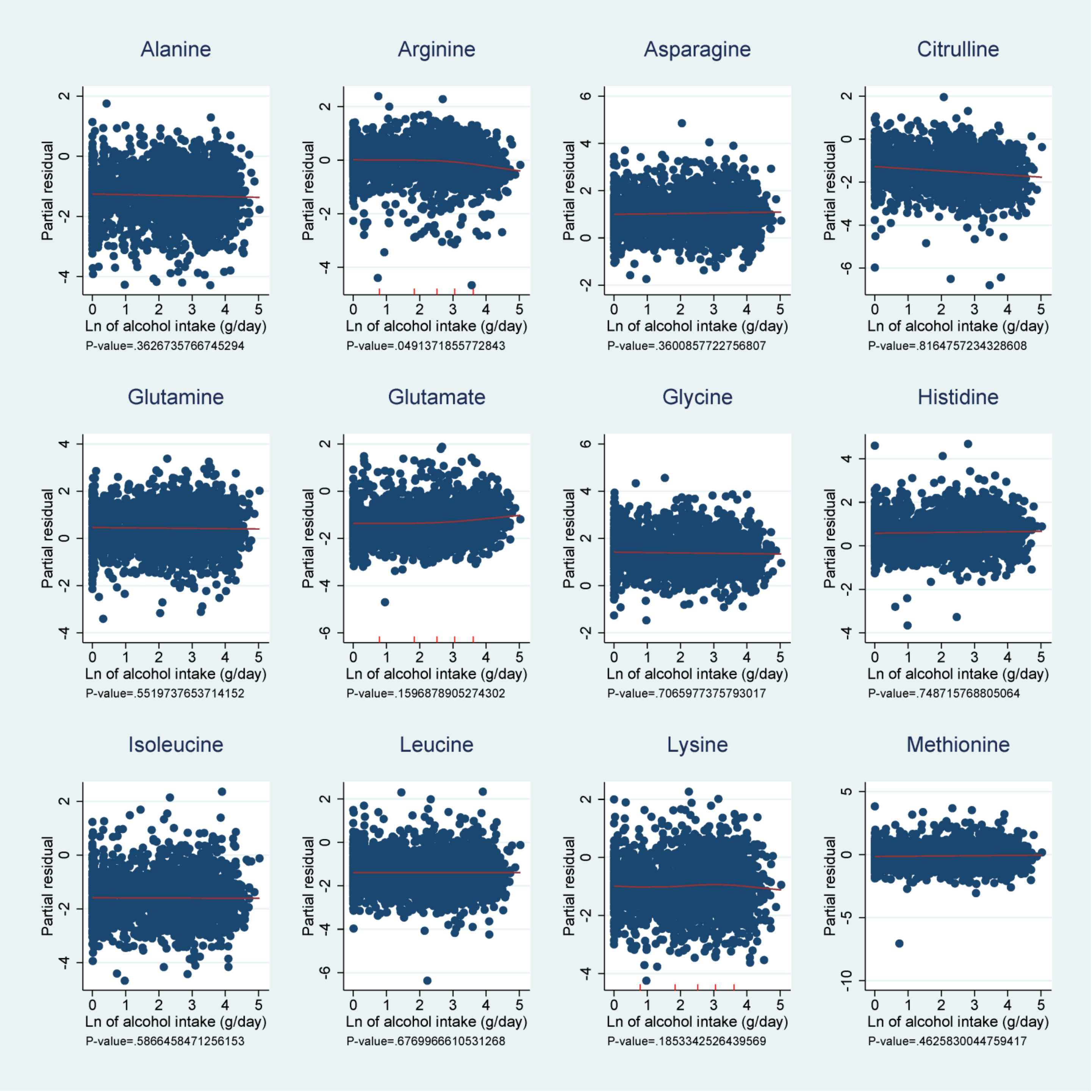
Alanine
Partial residual
Ln of alcohol intake (g/day)
P-value=.3626735766745294
Arginine
Partial residual
Ln of alcohol intake (g/day)
P-value=.0491371855772843
Asparagine
Partial residual
Ln of alcohol intake (g/day)
P-value=.3600857722756807
Citrulline
Partial residual
Ln of alcohol intake (g/day)
P-value=.8164757234328608
Glutamine
Partial residual
Ln of alcohol intake (g/day)
P-value=.5519737653714152
Glutamate
Partial residual
Ln of alcohol intake (g/day)
P-value=.1596878905274302
Glycine
Partial residual
Ln of alcohol intake (g/day)
P-value=.7065977375793017
Histidine
Partial residual
Ln of alcohol intake (g/day)
P-value=.748715768805064
Isoleucine
Partial residual
Ln of alcohol intake (g/day)
P-value=.5866458471256153
Leucine
Partial residual
Ln of alcohol intake (g/day)
P-value=.6769966610531268
Lysine
Partial residual
Ln of alcohol intake (g/day)
P-value=.1853342526439569
Methionine
Partial residual
Ln of alcohol intake (g/day)
P-value=.4625830044759417

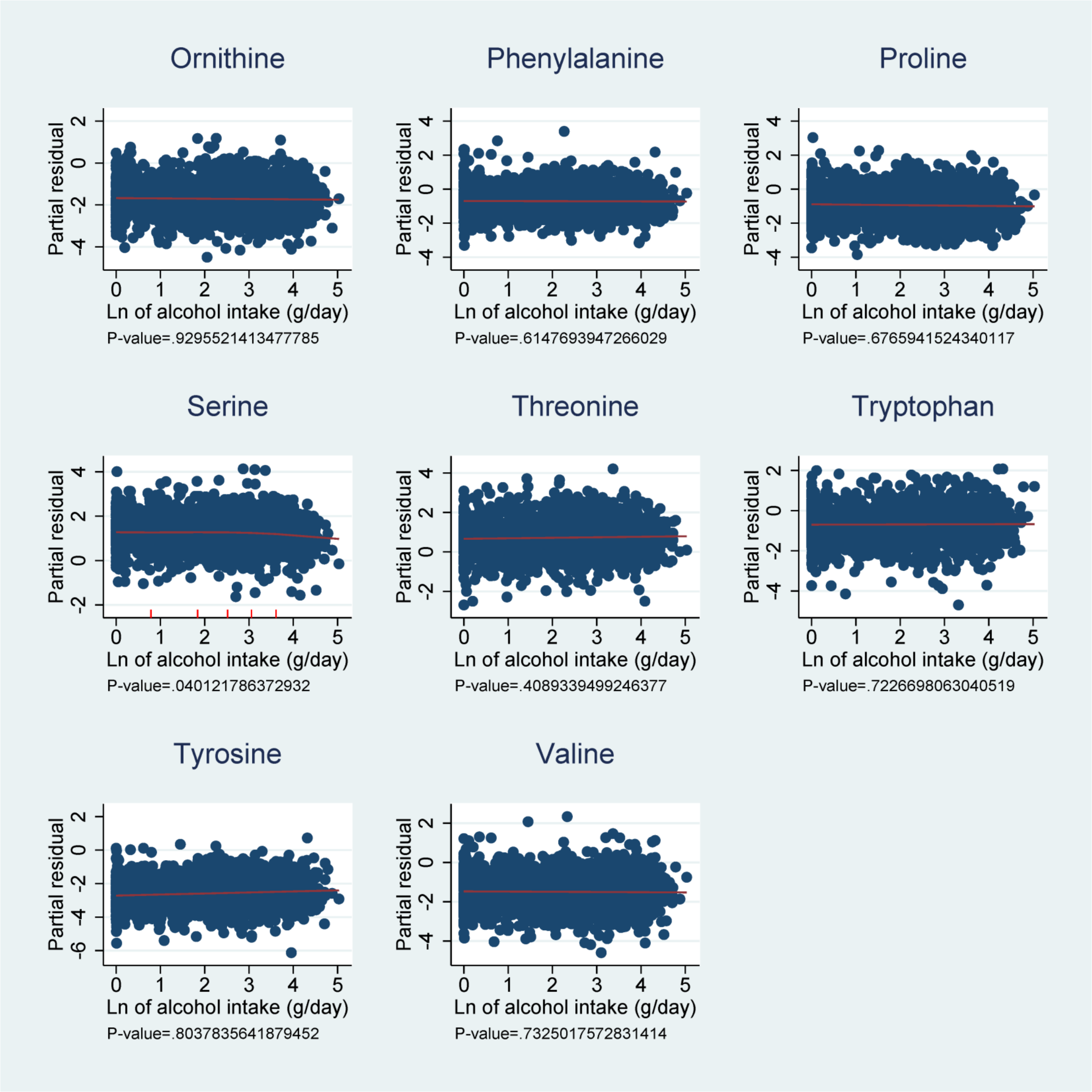
Ornithine
Phenylalanine
Proline
Serine
Threonine
Tryptophan
Tyrosine
Valine
Partial residual
Ln of alcohol intake (g/day)
P-value=.9295521413477785
P-value=.6147693947266029
P-value=.6765941524340117
P-value=.040121786372932
P-value=.4089339499246377
P-value=.7226698063040519
P-value=.8037835641879452
P-value=.7325017572831414

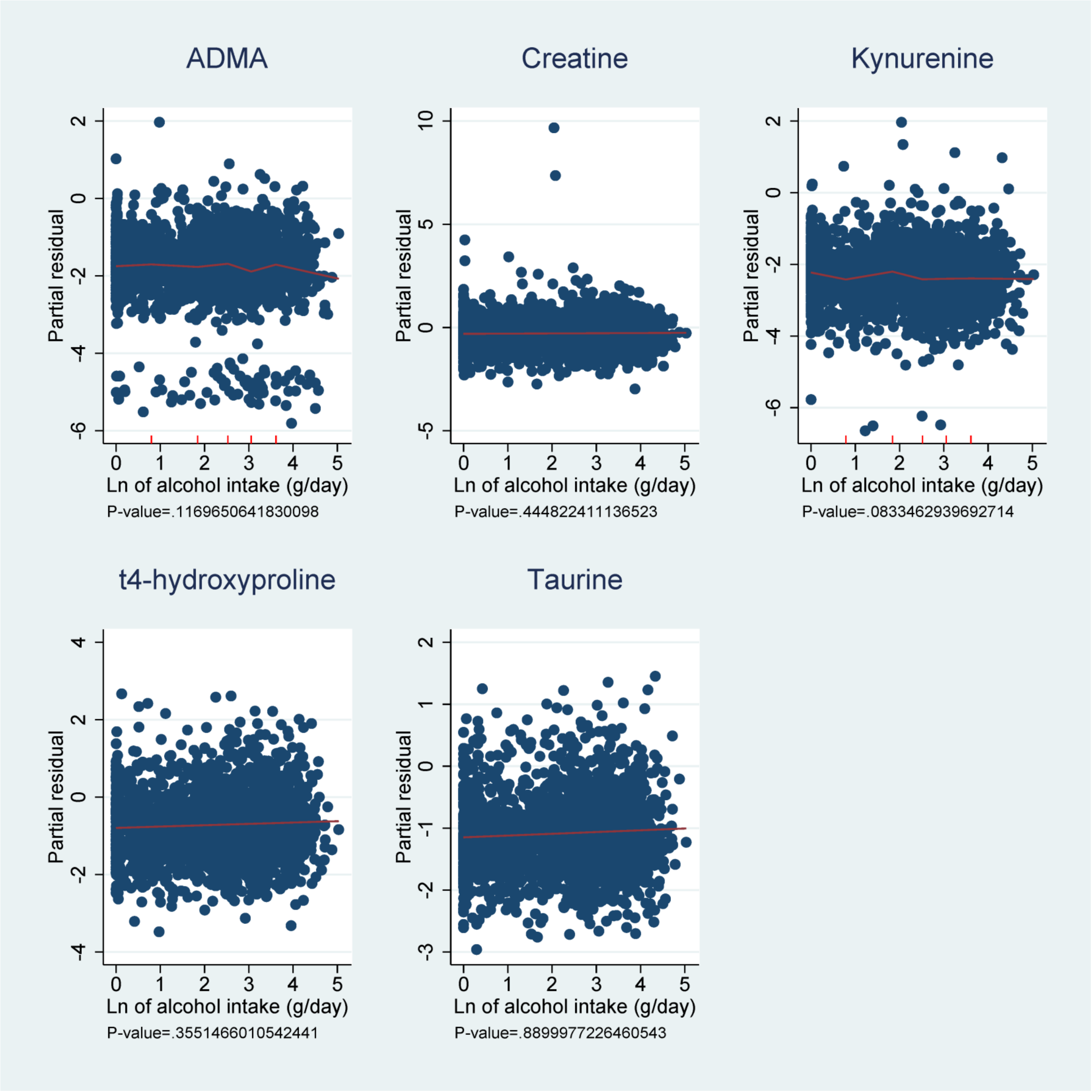
ADMA
Creatine
Kynurenine
t4-hydroxyproline
Taurine
Partial residual
Ln of alcohol intake (g/day)
P-value=.1169650641830098
P-value=.444822411136523
P-value=.0833462939692714
P-value=.3551466010542441
P-value=.8899977226460543

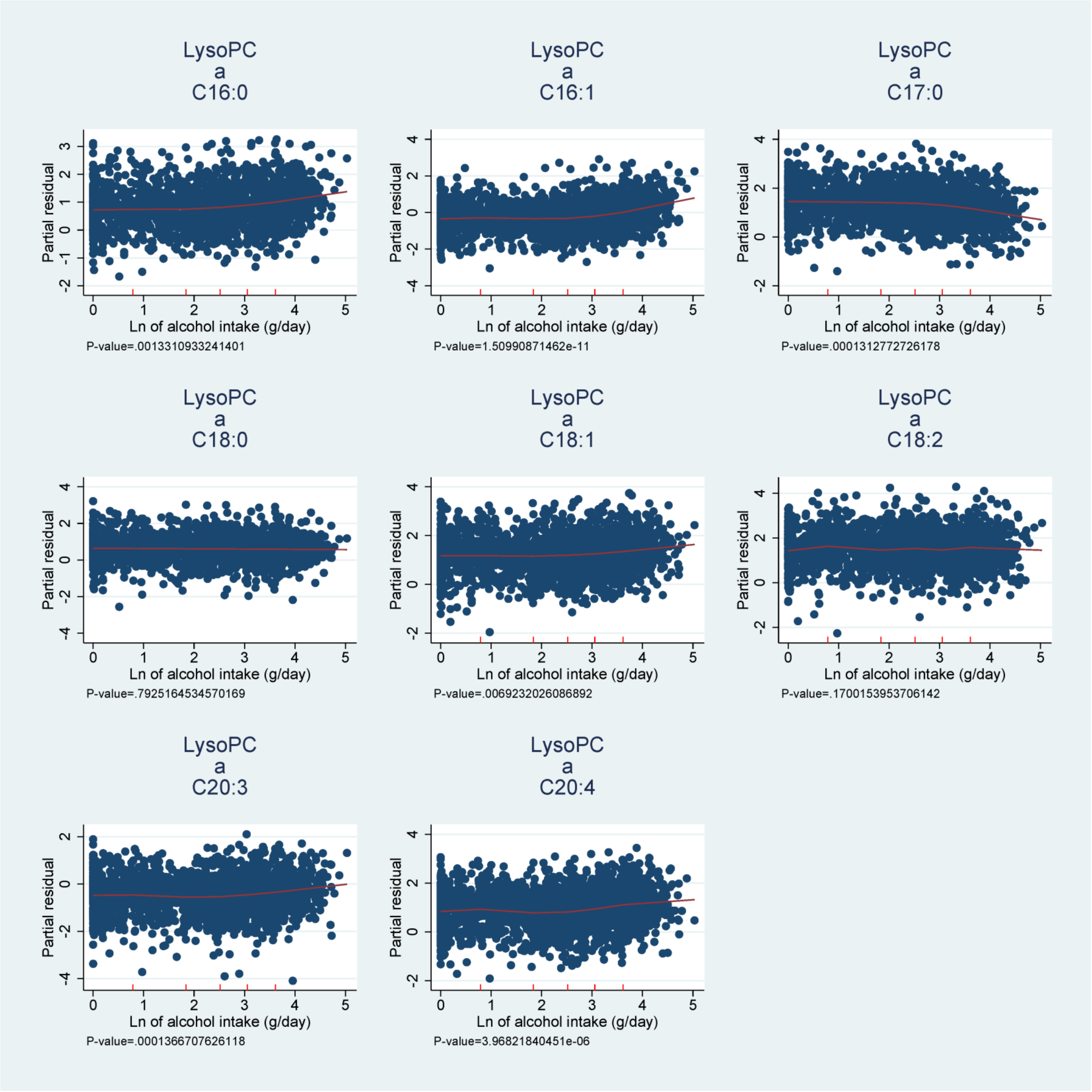
LysoPC a C16:0
Partial residual
Ln of alcohol intake (g/day)
P-value=.0013310933241401
LysoPC a C16:1
Partial residual
Ln of alcohol intake (g/day)
P-value=1.50990871462e-11
LysoPC a C17:0
Partial residual
Ln of alcohol intake (g/day)
P-value=.0001312772726178
LysoPC a C18:0
Partial residual
Ln of alcohol intake (g/day)
P-value=.7925164534570169
LysoPC a C18:1
Partial residual
Ln of alcohol intake (g/day)
P-value=.0069232026086892
LysoPC a C18:2
Partial residual
Ln of alcohol intake (g/day)
P-value=.1700153953706142
LysoPC a C20:3
Partial residual
Ln of alcohol intake (g/day)
P-value=.0001366707626118
LysoPC a C20:4
Partial residual
Ln of alcohol intake (g/day)
P-value=3.96821840451e-06

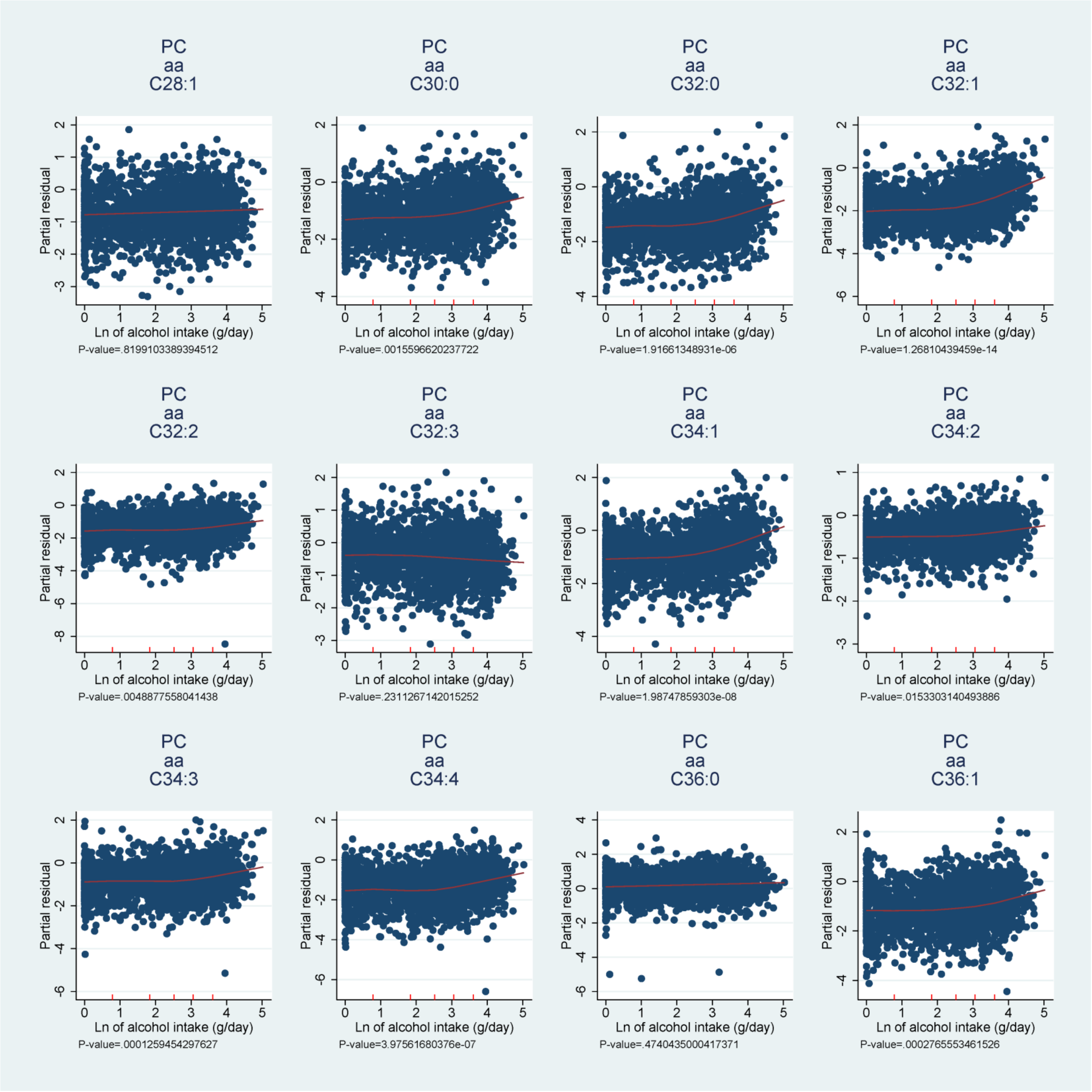
PC aa C28:1
Partial residual
Ln of alcohol intake (g/day)
P-value=.8199103389394512
PC aa C30:0
Partial residual
Ln of alcohol intake (g/day)
P-value=.0015596620237722
PC aa C32:0
Partial residual
Ln of alcohol intake (g/day)
P-value=1.91661348931e-06
PC aa C32:1
Partial residual
Ln of alcohol intake (g/day)
P-value=1.26810439459e-14
PC aa C32:2
Partial residual
Ln of alcohol intake (g/day)
P-value=.0048877558041438
PC aa C32:3
Partial residual
Ln of alcohol intake (g/day)
P-value=.2311267142015252
PC aa C34:1
Partial residual
Ln of alcohol intake (g/day)
P-value=1.98747859303e-08
PC aa C34:2
Partial residual
Ln of alcohol intake (g/day)
P-value=.0153303140493886
PC aa C34:3
Partial residual
Ln of alcohol intake (g/day)
P-value=.0001259454297627
PC aa C34:4
Partial residual
Ln of alcohol intake (g/day)
P-value=3.97561680376e-07
PC aa C36:0
Partial residual
Ln of alcohol intake (g/day)
P-value=.4740435000417371
PC aa C36:1
Partial residual
Ln of alcohol intake (g/day)
P-value=.0002765553461526

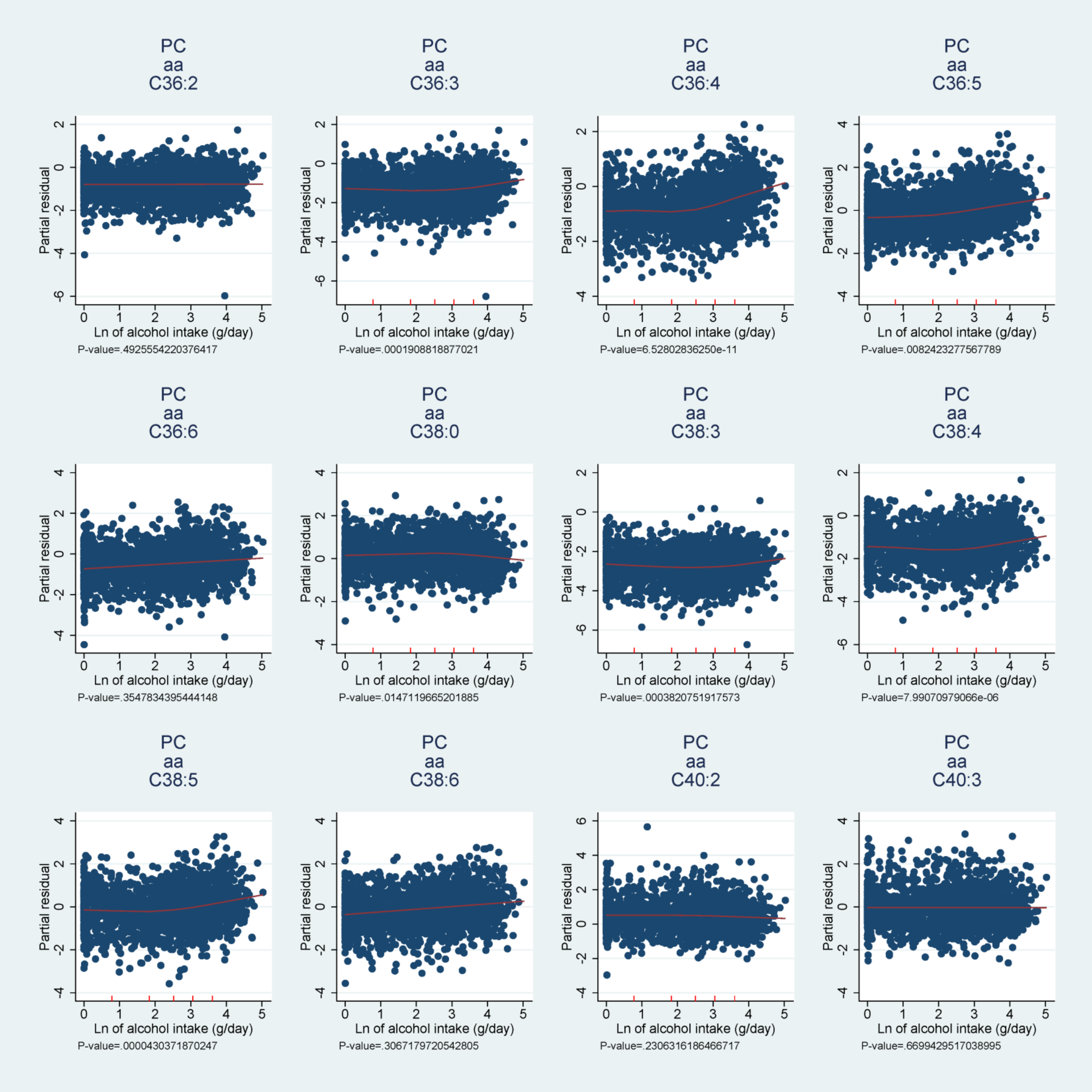
PC aa C36:2
Partial residual
Ln of alcohol intake (g/day)
P-value=.4925554220376417
PC aa C36:3
Partial residual
Ln of alcohol intake (g/day)
P-value=.0001908818877021
PC aa C36:4
Partial residual
Ln of alcohol intake (g/day)
P-value=6.52802836250e-11
PC aa C36:5
Partial residual
Ln of alcohol intake (g/day)
P-value=.0082423277567789
PC aa C36:6
Partial residual
Ln of alcohol intake (g/day)
P-value=.3547834395444148
PC aa C38:0
Partial residual
Ln of alcohol intake (g/day)
P-value=.0147119665201885
PC aa C38:3
Partial residual
Ln of alcohol intake (g/day)
P-value=.0003820751917573
PC aa C38:4
Partial residual
Ln of alcohol intake (g/day)
P-value=7.99070979066e-06
PC aa C38:5
Partial residual
Ln of alcohol intake (g/day)
P-value=.0000430371870247
PC aa C38:6
Partial residual
Ln of alcohol intake (g/day)
P-value=.3067179720542805
PC aa C40:2
Partial residual
Ln of alcohol intake (g/day)
P-value=.2306316186466717
PC aa C40:3
Partial residual
Ln of alcohol intake (g/day)
P-value=.6699429517038995

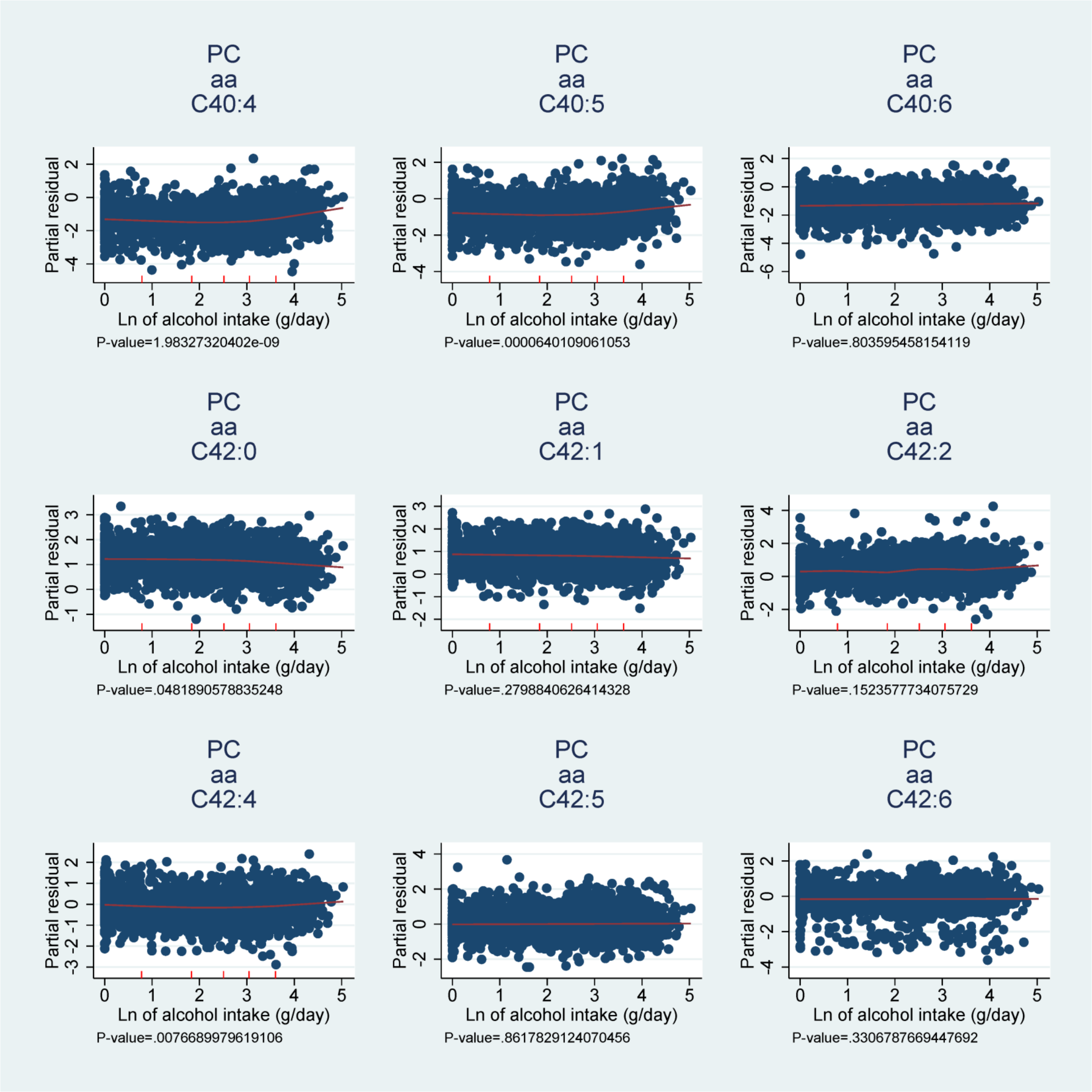
PC
aa
C40:4
Partial residual
Ln of alcohol intake (g/day)
P-value=1.98327320402e-09
PC
aa
C40:5
Partial residual
Ln of alcohol intake (g/day)
P-value=.0000640109061053
PC
aa
C40:6
Partial residual
Ln of alcohol intake (g/day)
P-value=.803595458154119
PC
aa
C42:0
Partial residual
Ln of alcohol intake (g/day)
P-value=.0481890578835248
PC
aa
C42:1
Partial residual
Ln of alcohol intake (g/day)
P-value=.2798840626414328
PC
aa
C42:2
Partial residual
Ln of alcohol intake (g/day)
P-value=.1523577734075729
PC
aa
C42:4
Partial residual
Ln of alcohol intake (g/day)
P-value=.0076689979619106
PC
aa
C42:5
Partial residual
Ln of alcohol intake (g/day)
P-value=.8617829124070456
PC
aa
C42:6
Partial residual
Ln of alcohol intake (g/day)
P-value=.3306787669447692

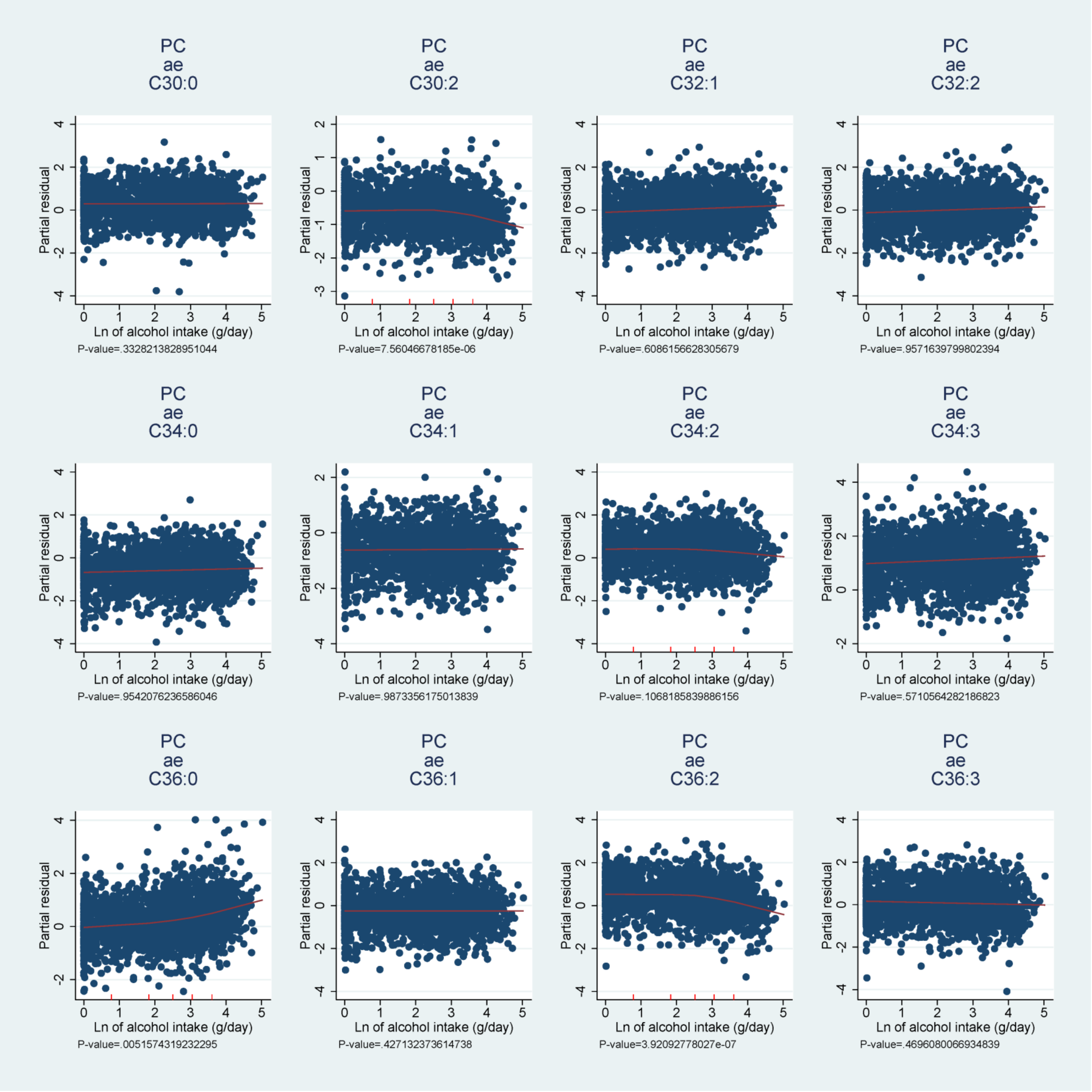
PC ae C30:0
Partial residual
Ln of alcohol intake (g/day)
P-value=.3328213828951044
PC ae C30:2
Partial residual
Ln of alcohol intake (g/day)
P-value=7.56046678185e-06
PC ae C32:1
Partial residual
Ln of alcohol intake (g/day)
P-value=.6086156628305679
PC ae C32:2
Partial residual
Ln of alcohol intake (g/day)
P-value=.9571639799802394
PC ae C34:0
Partial residual
Ln of alcohol intake (g/day)
P-value=.9542076236586046
PC ae C34:1
Partial residual
Ln of alcohol intake (g/day)
P-value=.9873356175013839
PC ae C34:2
Partial residual
Ln of alcohol intake (g/day)
P-value=.1068185839886156
PC ae C34:3
Partial residual
Ln of alcohol intake (g/day)
P-value=.5710564282186823
PC ae C36:0
Partial residual
Ln of alcohol intake (g/day)
P-value=.0051574319232295
PC ae C36:1
Partial residual
Ln of alcohol intake (g/day)
P-value=.427132373614738
PC ae C36:2
Partial residual
Ln of alcohol intake (g/day)
P-value=3.92092778027e-07
PC ae C36:3
Partial residual
Ln of alcohol intake (g/day)
P-value=.4696080066934839

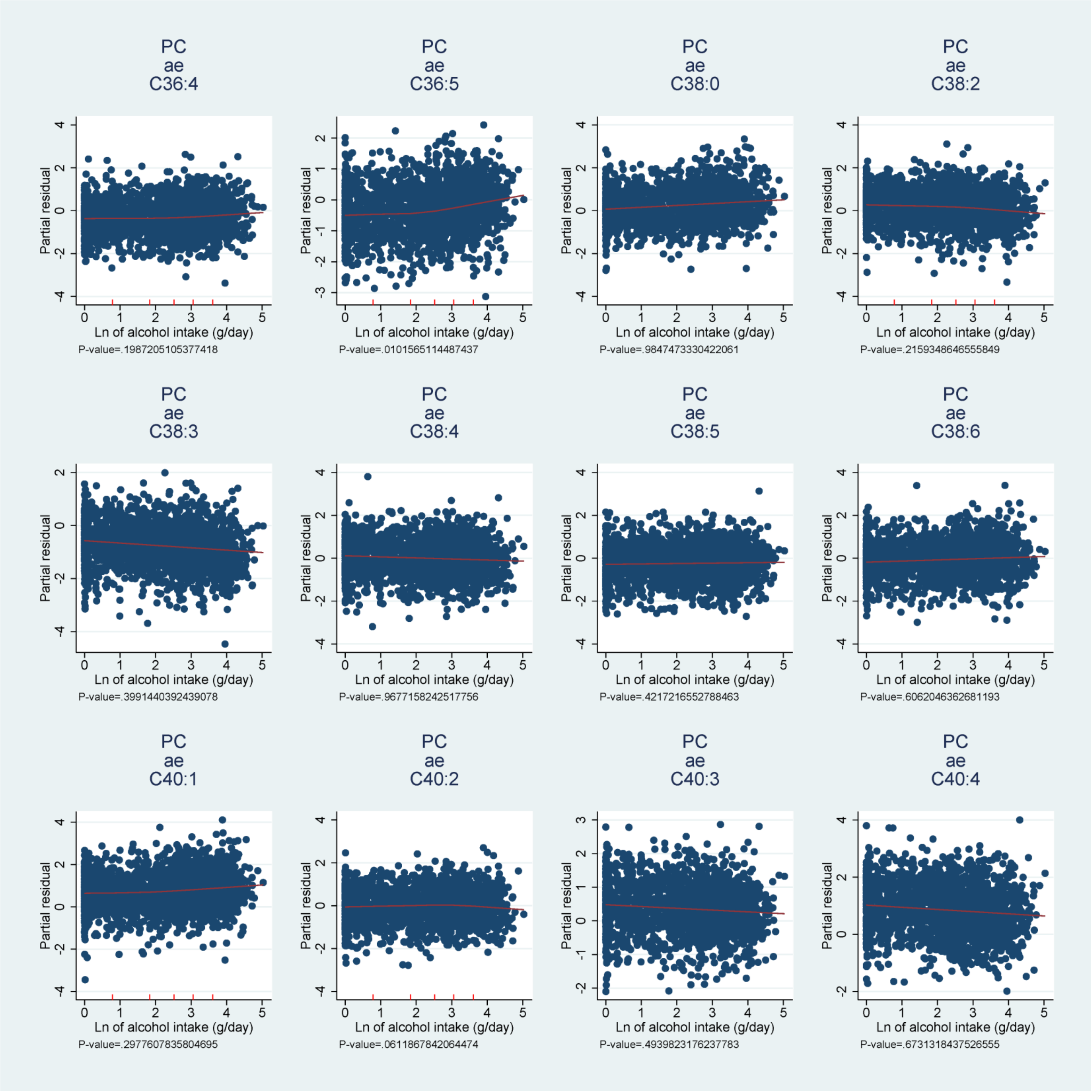
PC ae C36:4
Partial residual
Ln of alcohol intake (g/day)
P-value=.1987205105377418
PC ae C36:5
Partial residual
Ln of alcohol intake (g/day)
P-value=.0101565114487437
PC ae C38:0
Partial residual
Ln of alcohol intake (g/day)
P-value=.9847473330422061
PC ae C38:2
Partial residual
Ln of alcohol intake (g/day)
P-value=.2159348646555849
PC ae C38:3
Partial residual
Ln of alcohol intake (g/day)
P-value=.3991440392439078
PC ae C38:4
Partial residual
Ln of alcohol intake (g/day)
P-value=.9677158242517756
PC ae C38:5
Partial residual
Ln of alcohol intake (g/day)
P-value=.4217216552788463
PC ae C38:6
Partial residual
Ln of alcohol intake (g/day)
P-value=.6062046362681193
PC ae C40:1
Partial residual
Ln of alcohol intake (g/day)
P-value=.2977607835804695
PC ae C40:2
Partial residual
Ln of alcohol intake (g/day)
P-value=.0611867842064474
PC ae C40:3
Partial residual
Ln of alcohol intake (g/day)
P-value=.4939823176237783
PC ae C40:4
Partial residual
Ln of alcohol intake (g/day)
P-value=.6731318437526555

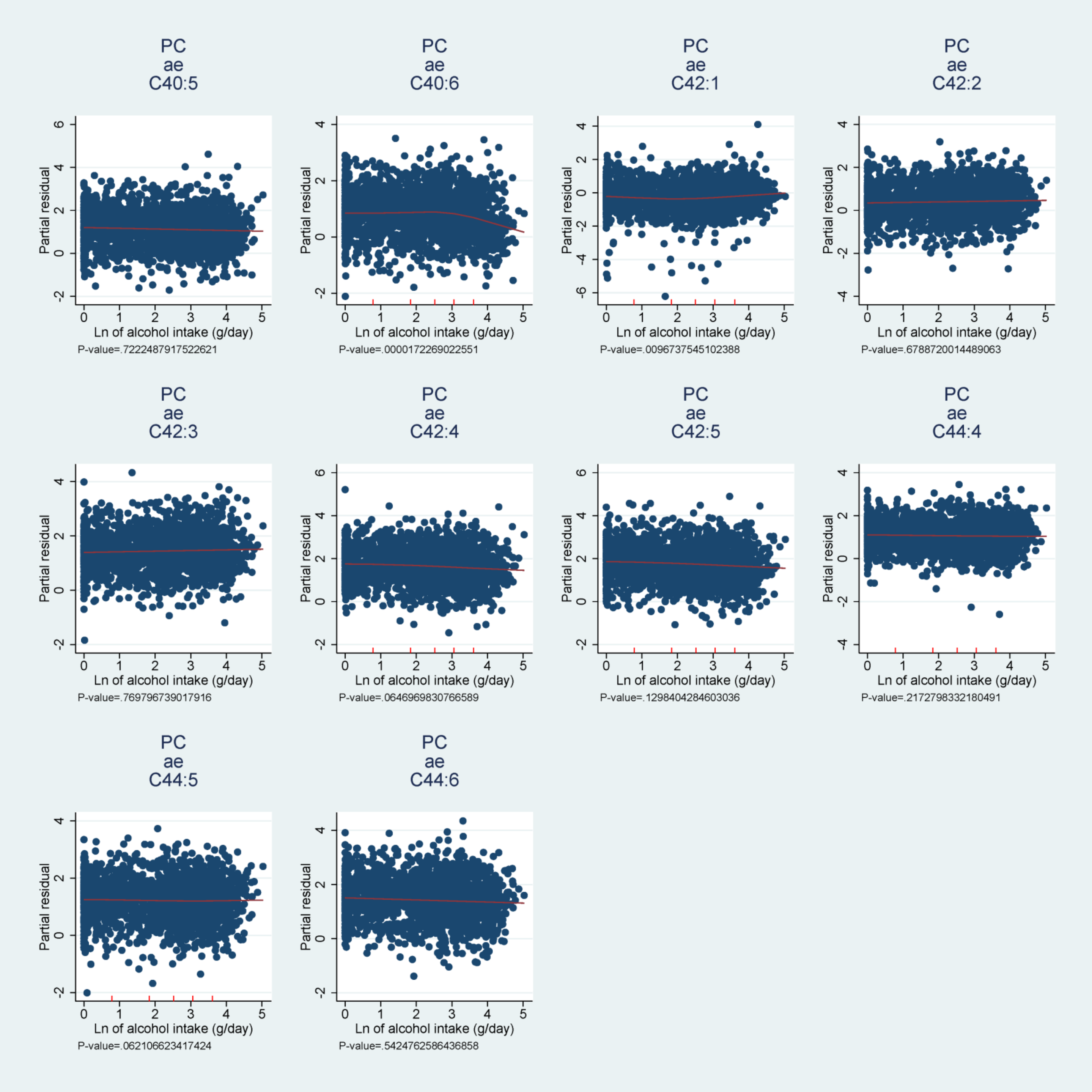
PC ae C40:5
Partial residual
Ln of alcohol intake (g/day)
P-value=.7222487917522621
PC ae C40:6
Partial residual
Ln of alcohol intake (g/day)
P-value=.0000172269022551
PC ae C42:1
Partial residual
Ln of alcohol intake (g/day)
P-value=.0096737545102388
PC ae C42:2
Partial residual
Ln of alcohol intake (g/day)
P-value=.6788720014489063
PC ae C42:3
Partial residual
Ln of alcohol intake (g/day)
P-value=.769796739017916
PC ae C42:4
Partial residual
Ln of alcohol intake (g/day)
P-value=.0646969830766589
PC ae C42:5
Partial residual
Ln of alcohol intake (g/day)
P-value=.1298404284603036
PC ae C44:4
Partial residual
Ln of alcohol intake (g/day)
P-value=.2172798332180491
PC ae C44:5
Partial residual
Ln of alcohol intake (g/day)
P-value=.062106623417424
PC ae C44:6
Partial residual
Ln of alcohol intake (g/day)
P-value=.5424762586436858

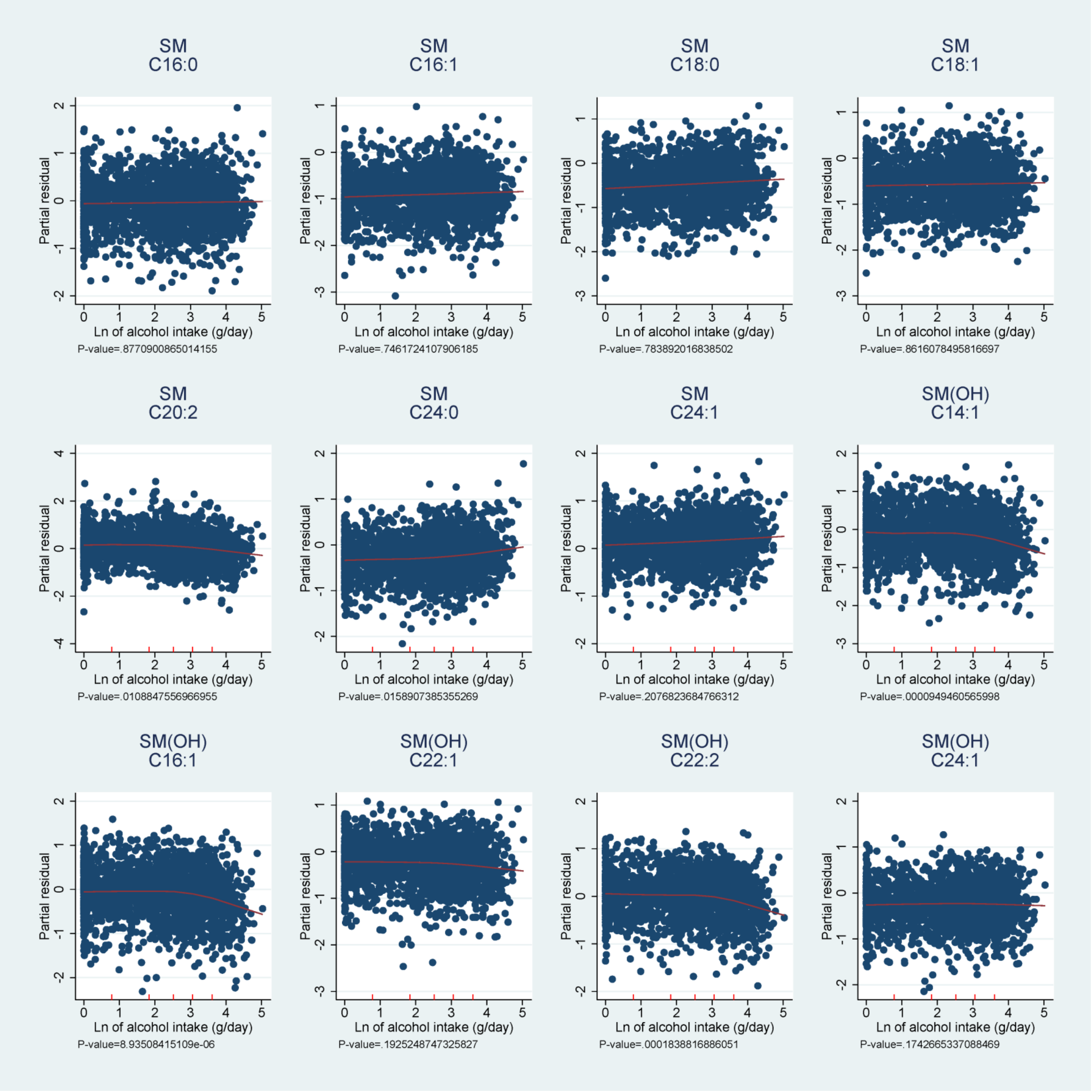
SM C16:0
Partial residual
Ln of alcohol intake (g/day)
P-value=.8770900865014155
SM C16:1
Partial residual
Ln of alcohol intake (g/day)
P-value=.7461724107906185
SM C18:0
Partial residual
Ln of alcohol intake (g/day)
P-value=.783892016838502
SM C18:1
Partial residual
Ln of alcohol intake (g/day)
P-value=.8616078495816697
SM C20:2
Partial residual
Ln of alcohol intake (g/day)
P-value=.0108847556966955
SM C24:0
Partial residual
Ln of alcohol intake (g/day)
P-value=.0158907385355269
SM C24:1
Partial residual
Ln of alcohol intake (g/day)
P-value=.2076823684766312
SM(OH) C14:1
Partial residual
Ln of alcohol intake (g/day)
P-value=.0000949460565998
SM(OH) C16:1
Partial residual
Ln of alcohol intake (g/day)
P-value=8.93508415109e-06
SM(OH) C22:1
Partial residual
Ln of alcohol intake (g/day)
P-value=.1925248747325827
SM(OH) C22:2
Partial residual
Ln of alcohol intake (g/day)
P-value=.0001838816886051
SM(OH) C24:1
Partial residual
Ln of alcohol intake (g/day)
P-value=.1742665337088469

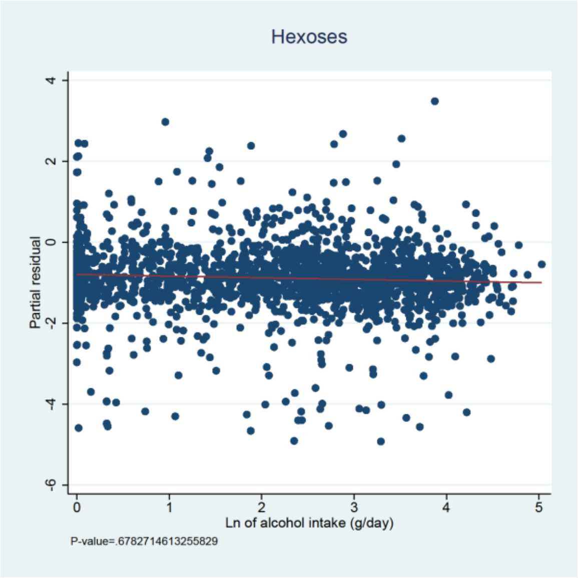
Hexoses
Partial residual
Ln of alcohol intake (g/day)
P-value=.6782714613255829

Supplementary Figure 5. Venn diagram showing overlap in metabolites identified as being statistically significantly (Bonferroni-adjusted p-value<0.05) associated with continuous ln-transformed alcohol intake (main analysis) and with heavy drinking (≥40 g/day of alcohol) *vs.* light drinking (0.1-4.9 g/day of alcohol) in replication analysis.

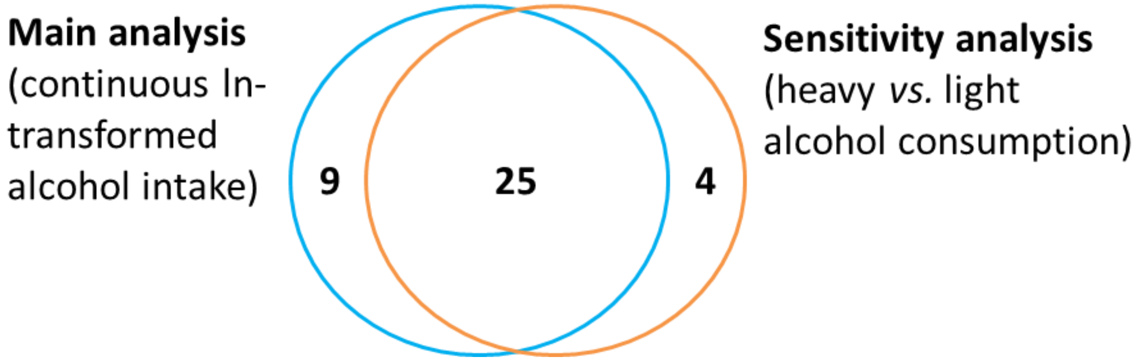

Supplementary Figure 6. Regression coefficients obtained from analysis of associations of alcohol intake with metabolites in the discovery dataset, stratified by sub-study, i.e. colorectal cancer (CRC), kidney cancer (KC), hepatobiliary cancer (HC), and prostate cancer (PC) case-control study.

Footnote: Analyzed with multivariable linear regression analyses analyzing associations of alcohol consumption (ln-transformed alcohol intake + 1) as main independent variable and as dependent variables the residuals obtained from linear mixed models with Z-standardized ln-transformed metabolite concentrations as dependent variables, sex as independent variable, and random intercepts for analytical batches nested within studies. Adjusted for: sex; age (y; continuous), body mass index (kg/m^2; continuous), self-reported physical activity levels (Cambridge physical activity index [22]: inactive, moderately inactive, moderately active, active, unknown), fasting status (≥6 hours, 3-5.9 hours, <3 hours, unknown), meat intake (g/day; continuous), fish intake (g/day; continuous), energy intake (kcal/day; continuous), country, and smoking status (current, former, never, unknown).

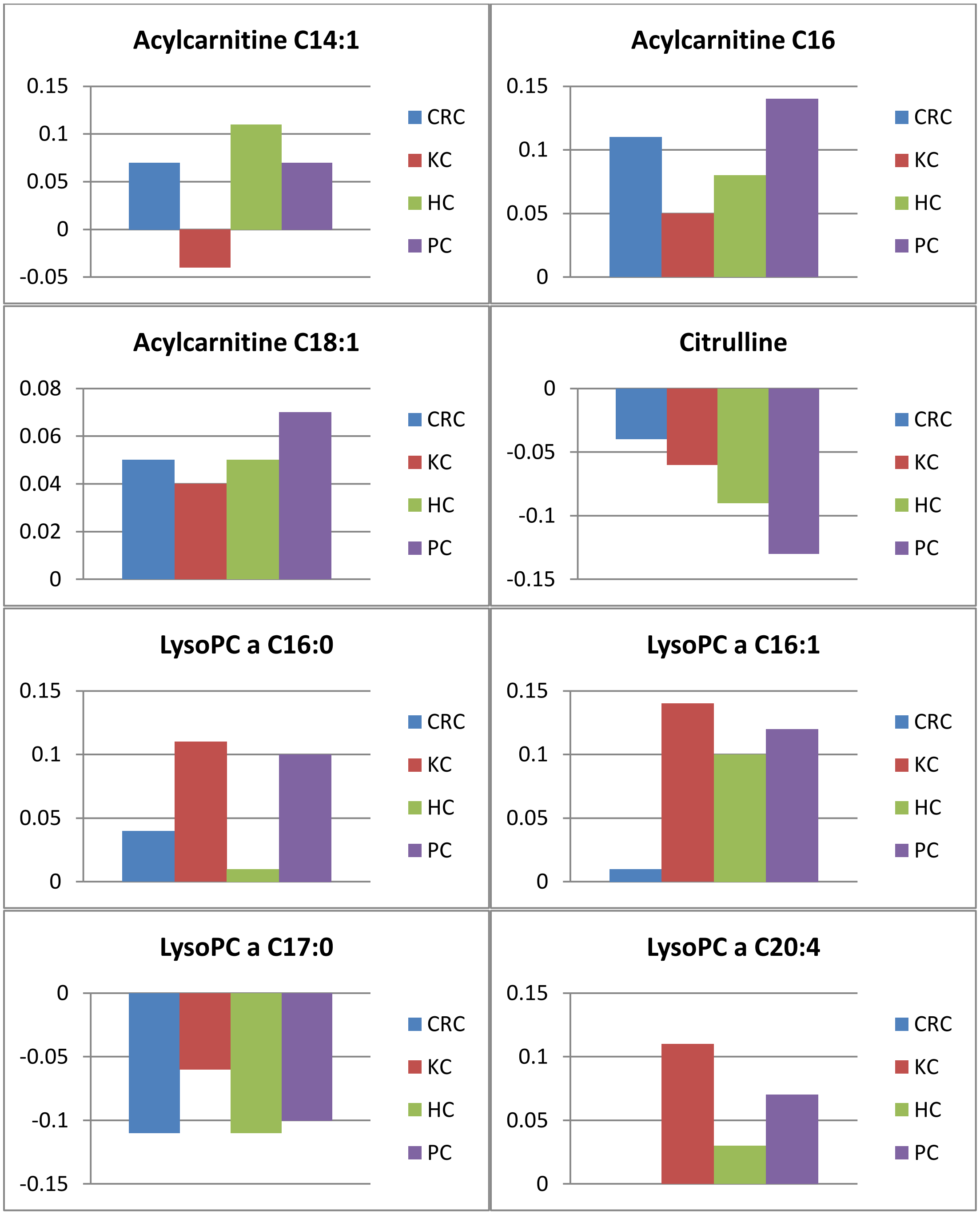

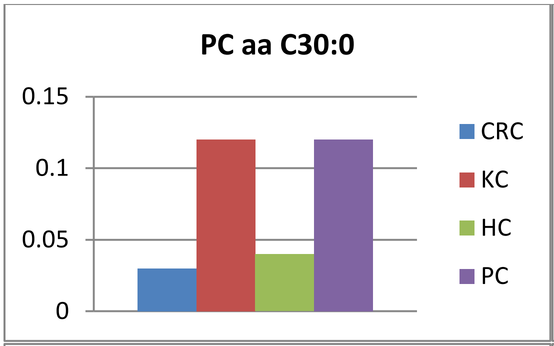
PC aa C30:0
0.15
0.1
0.05
0
CRC
KC
HC
PC

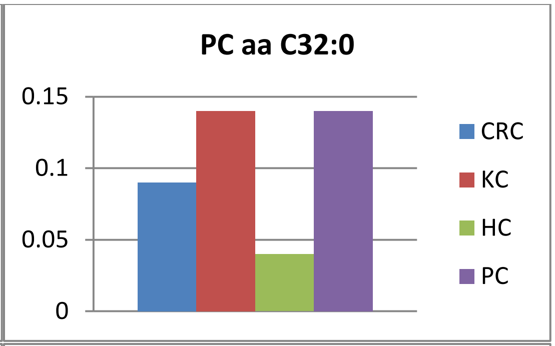
PC aa C32:0
0.15
0.1
0.05
0
CRC
KC
HC
PC

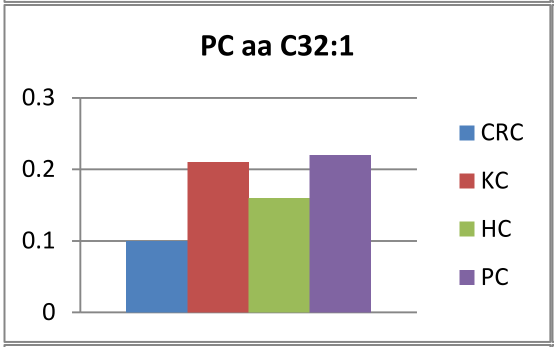
PC aa C32:1
0.3
0.2
0.1
0
CRC
KC
HC
PC

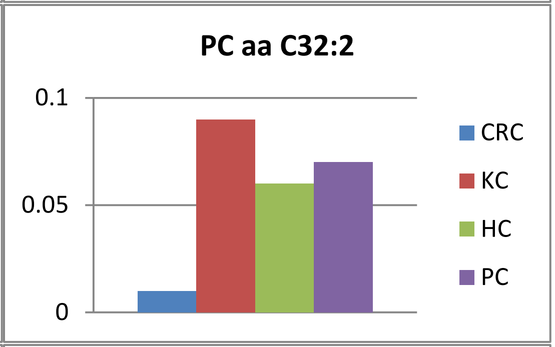
PC aa C32:2
0.1
0.05
0
CRC
KC
HC
PC

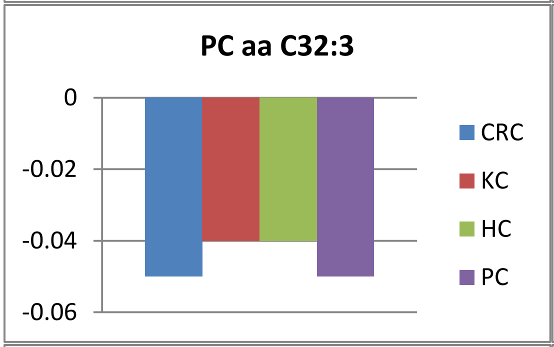
PC aa C32:3
0
-0.02
-0.04
-0.06
CRC
KC
HC
PC

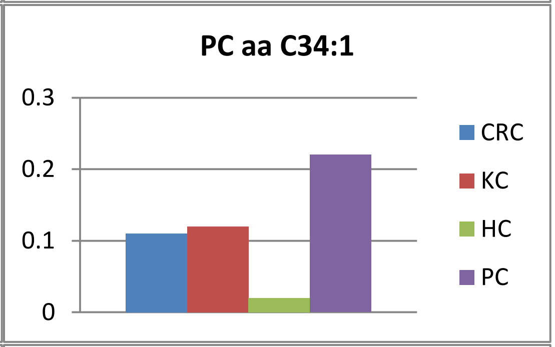
PC aa C34:1
0.3
0.2
0.1
0
CRC
KC
HC
PC

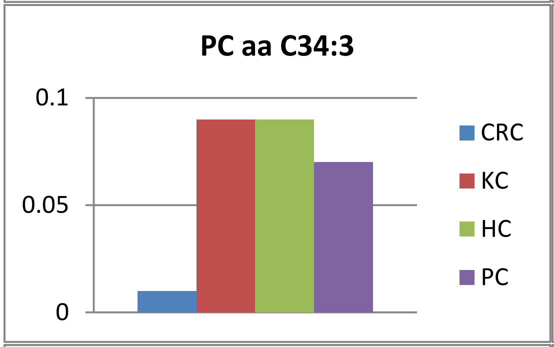
PC aa C34:3
0.1
0.05
0
CRC
KC
HC
PC

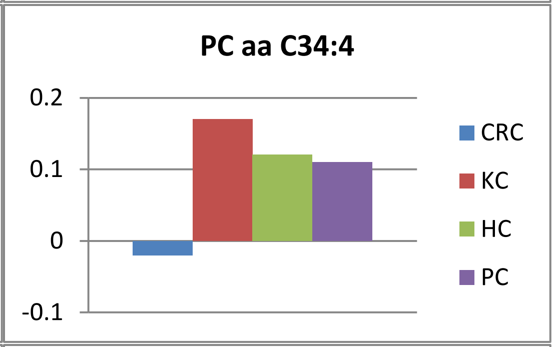
PC aa C34:4
0.2
0.1
0
-0.1
CRC
KC
HC
PC

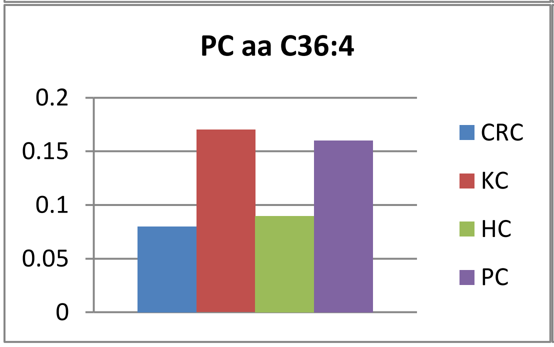
PC aa C36:4
0.2
0.15
0.1
0.05
0
CRC
KC
HC
PC

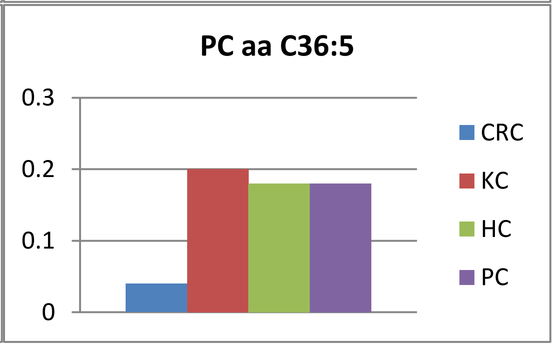
PC aa C36:5
0.3
0.2
0.1
0
CRC
KC
HC
PC

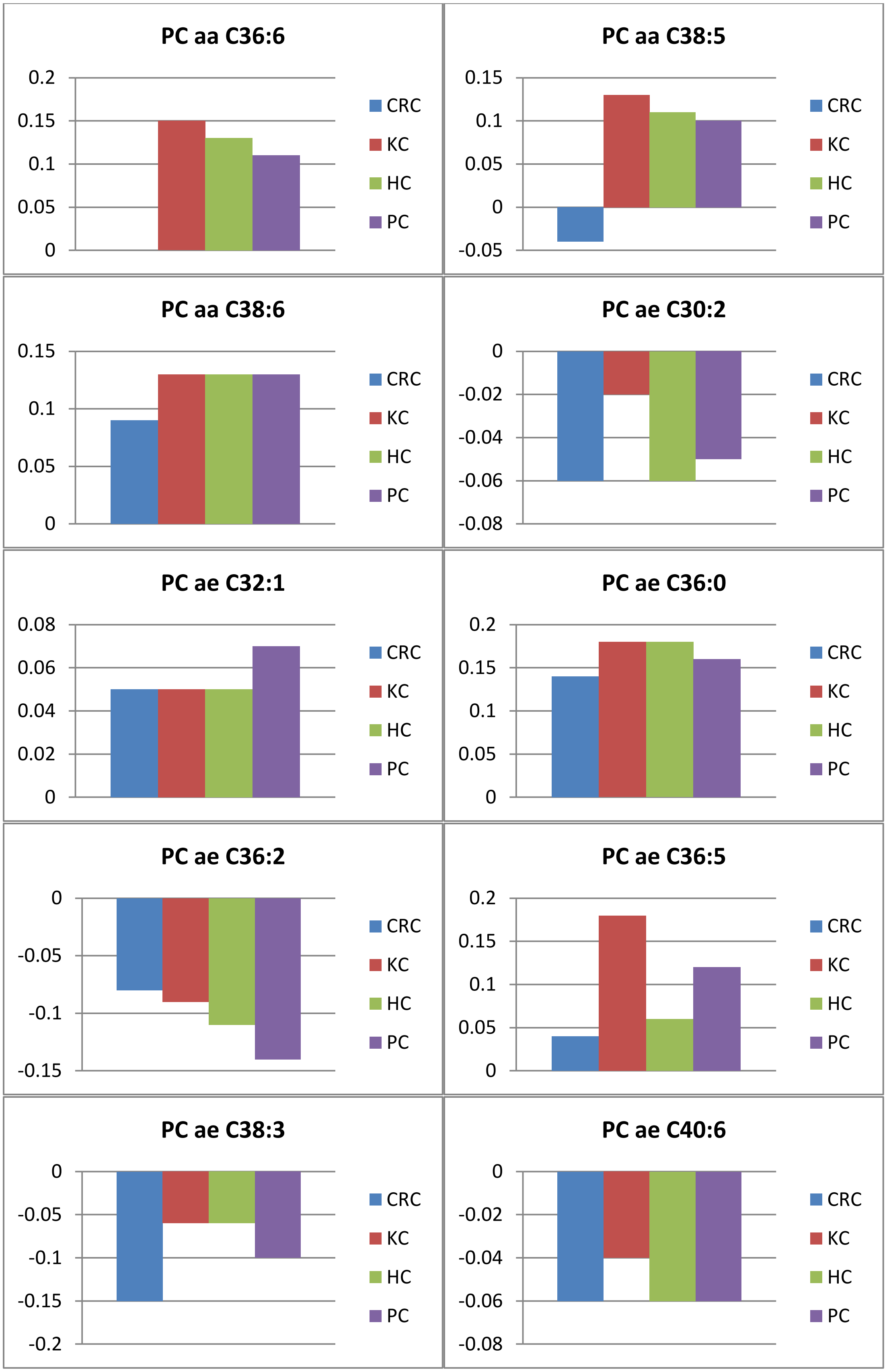
PC aa C36:6
0.2
0.15
0.1
0.05
0
CRC
KC
HC
PC
PC aa C38:5
0.15
0.1
0.05
0
-0.05
CRC
KC
HC
PC
PC aa C38:6
0.15
0.1
0.05
0
CRC
KC
HC
PC
PC ae C30:2
0
-0.02
-0.04
-0.06
-0.08
CRC
KC
HC
PC
PC ae C32:1
0.08
0.06
0.04
0.02
0
CRC
KC
HC
PC
PC ae C36:0
0.2
0.15
0.1
0.05
0
CRC
KC
HC
PC
PC ae C36:2
0
-0.05
-0.1
-0.15
CRC
KC
HC
PC
PC ae C36:5
0.2
0.15
0.1
0.05
0
CRC
KC
HC
PC
PC ae C38:3
0
-0.05
-0.1
-0.15
-0.2
CRC
KC
HC
PC
PC ae C40:6
0
-0.02
-0.04
-0.06
-0.08
CRC
KC
HC
PC

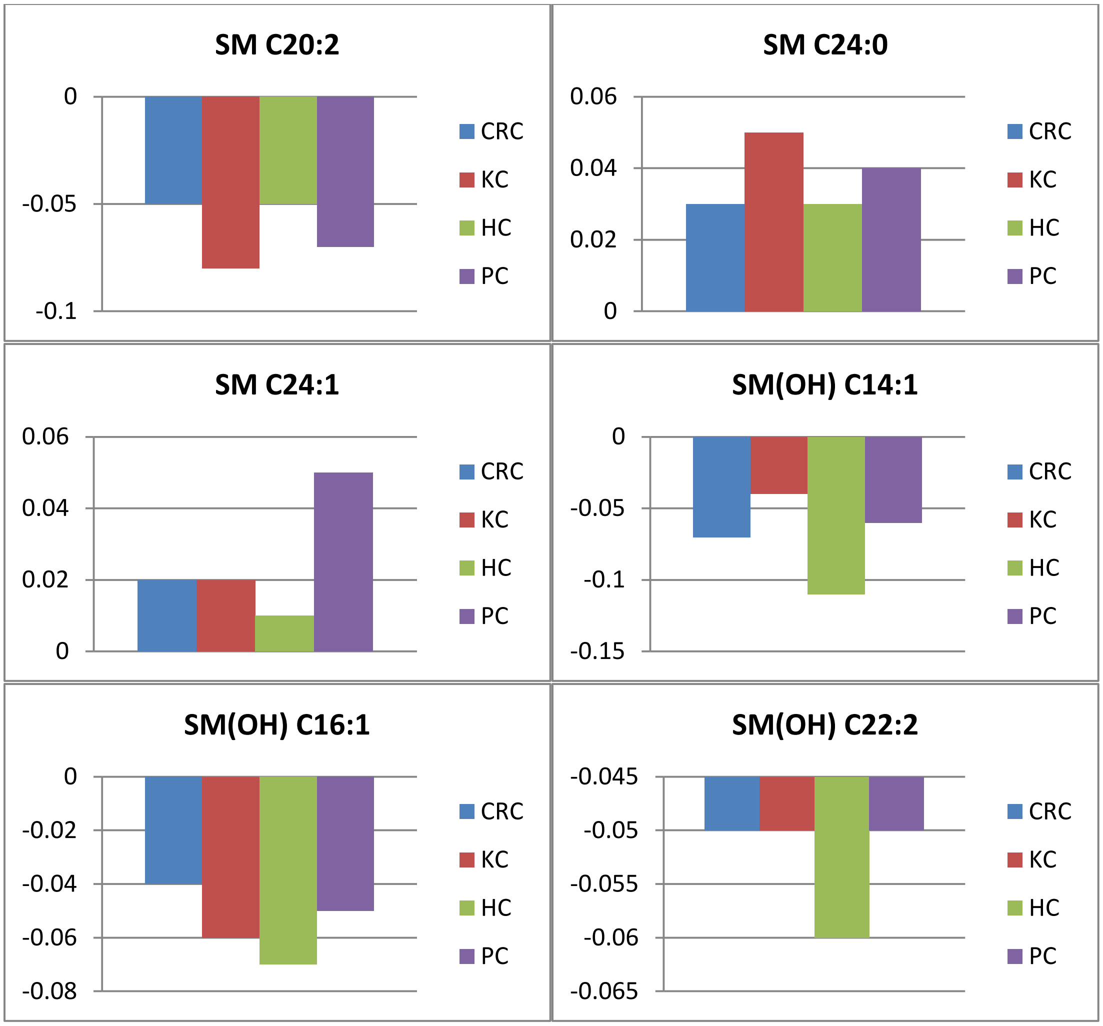
SM C20:2
0
-0.05
-0.1
CRC
KC
HC
PC
SM C24:0
0.06
0.04
0.02
0
CRC
KC
HC
PC
SM C24:1
0.06
0.04
0.02
0
CRC
KC
HC
PC
SM(OH) C14:1
0
-0.05
-0.1
-0.15
CRC
KC
HC
PC
SM(OH) C16:1
0
-0.02
-0.04
-0.06
-0.08
CRC
KC
HC
PC
SM(OH) C22:2
-0.045
-0.05
-0.055
-0.06
-0.065
CRC
KC
HC
PC
