# Supplementary material for: Circulating Metabolites Associated with Alcohol Intake in the European Prospective Investigation into Cancer and Nutrition Cohort
Source: Nutrients. 2018 May 22;10(5):654. doi: 10.3390/nu10050654 (PMC5986533; doi:10.3390/nu10050654)
Supplement: Supplementary file 1 [file nutrients-10-00654-s001.zip › Suppl Tables.pdf]

Supplementary Table 1. Information on measurements of metabolites included in the BIOCRATES AbsoluteIDQ^{TM} p180 kit in the total dataset and in each sub-study, and whether the metabolite was included for the current analysis.

			Total dataset (n=2,974)						Hepatobiliary cancer controls (n=327)		Colorectal cancer controls (n=491)		Prostate cancer controls (n=1,521)		Kidney cancer controls (n=635)	
Metabolite class	Biochemical name	Abbreviation	Detected in ≥1 sub-study	Perc. missing	Perc. <LOD or <LOQ	Perc. above quantify-cation	Total perc. missing /outside limits	Included for analysis	Detected	Total perc. missing /outside limits	Detected	Total perc. missing /outside limits	Detected	Total perc. missing /outside limits	Detected	Total perc. missing /outside limits
Acylcarnitines	Carnitine	C0	Yes	0.0	0.0	0.0	0.0	Yes	Yes	0.0	Yes	0.0	Yes	0.0	Yes	0.0
Acylcarnitines	Acetylcarnitine	C2	Yes	0.0	0.3	0.0	0.3	Yes	Yes	0.0	Yes	0.0	Yes	0.7	Yes	0.0
Acylcarnitines	Propionylcarnitine	C3	Yes	1.3	6.0	0.0	7.3	Yes	Yes	11.9	Yes	0.0	Yes	9.1	Yes	6.3
Acylcarnitines	Propenoylcarnitine	C3:1	No	100.0	0.0	0.0	100.0	No	No	100.0	No	100.0	No	100.0	No	100.0
Acylcarnitines	Hydroxypropionaylcarnitine	C3-OH	No	100.0	0.0	0.0	100.0	No	No	100.0	No	100.0	No	100.0	No	100.0
Acylcarnitines	Butyrylcarnitine	C4	Yes	1.3	10.0	0.0	11.3	Yes	Yes	11.9	Yes	0.2	Yes	14.3	Yes	12.3
Acylcarnitines	Butenylcarnitine	C4:1	Yes	81.6	15.8	0.0	97.4	No	No	100.0	No	100.0	Yes	95.0	No	100.0
Acylcarnitines	Hydroxybutyrylcarnitine	C4-OH (C3-DC)	Yes	34.1	33.4	0.0	67.5	No	Yes	46.8	Yes	63.1	Yes	91.0	Yes	24.9
Acylcarnitines	Valerylcarnitine	C5	Yes	1.3	14.8	0.0	16.1	Yes	Yes	11.9	Yes	9.6	Yes	20.9	Yes	11.8
Acylcarnitines	Tiglylcarnitine	C5:1	No	100.0	0.0	0.0	100.0	No	No	100.0	No	100.0	No	100.0	No	100.0
Acylcarnitines	Glutarylcarnitine	C5-DC (C6-OH)	Yes	62.1	20.2	0.0	82.3	No	No	100.0	Yes	44.4	No	100.0	Yes	60.3
Acylcarnitines	Glutaconylcarnitine	C5-1-Dc	Yes	91.8	6.6	0.0	98.4	No	Yes	85.9	No	100.0	No	100.0	No	100.0
Acylcarnitines	Methylglutarylcarnitine	C5-M-Dc	No	100.0	0.0	0.0	100.0	No	No	100.0	No	100.0	No	100.0	No	100.0
Acylcarnitines	Hydroxyvalerylcarnitine	C5-OH (C3-DC-M)	No	100.0	0.0	0.0	100.0	No	No	100.0	No	100.0	No	100.0	No	100.0
Acylcarnitines	Hexanoylcarnitine	C6 (C4:1-DC)	No	100.0	0.0	0.0	100.0	No	No	100.0	No	100.0	No	100.0	No	100.0
Acylcarnitines	Hexenoylcarnitine	C6:1	No	100.0	0.0	0.0	100.0	No	No	100.0	No	100.0	No	100.0	No	100.0
Acylcarnitines	Pimelylcarnitine	C7-Dc	Yes	69.0	27.0	0.0	96.0	No	Yes	78.6	No	100.0	No	100.0	Yes	92.1
Acylcarnitines	Octanoylcarnitine	C8	Yes	62.1	36.7	0.0	98.8	No	No	100.0	Yes	98.2	No	100.0	Yes	95.8
Acylcarnitines	Nonaylcarnitine	C9	Yes	78.7	14.4	0.0	93.1	No	No	100.0	No	100.0	No	100.0	Yes	67.4
Acylcarnitines	Decanoylcarnitine	C10	Yes	52.5	27.1	0.0	79.6	No	Yes	48.6	Yes	82.5	No	100.0	Yes	44.3
Acylcarnitines	Decenoylcarnitine	C10:1	Yes	1.3	42.4	0.0	43.7	No	Yes	34.5	Yes	22.8	Yes	38.1	Yes	78.0
Acylcarnitines	Decadienylcarnitine	C10:2	No	100.0	0.0	0.0	100.0	No	No	100.0	No	100.0	No	100.0	No	100.0
Acylcarnitines	Dodecanoylcarnitine	C12	Yes	19.7	44.5	0.0	64.2	No	Yes	64.5	Yes	42.8	Yes	70.8	Yes	65.0
Acylcarnitines	Dodecenoylcarnitine	C12:1	Yes	22.7	25.4	0.0	48.1	No	Yes	76.1	Yes	12.4	Yes	31.9	No	100.0
Acylcarnitines	Dodecanedioylcarnitine	C12-DC	No	100.0	0.0	0.0	100.0	No	No	100.0	No	100.0	No	100.0	No	100.0
Acylcarnitines	Tetradecanoylcarnitine	C14	Yes	83.5	10.4	0.0	93.9	No	No	100.0	Yes	63.1	No	100.0	No	100.0
Acylcarnitines	Tetradecenoylcarnitine	C14:1	Yes	1.3	6.0	0.0	7.3	Yes	Yes	12.5	Yes	0.0	Yes	7.0	Yes	11.0
Acylcarnitines	Hydroxytetradecenoyl-carnitine	C14:1-OH	No	100.0	0.0	0.0	100.0	No	No	100.0	No	100.0	No	100.0	No	100.0
Acylcarnitines	Tetradecadienylcarnitine	C14:2	Yes	1.3	44.0	0.0	45.3	No	Yes	48.6	Yes	28.3	Yes	47.0	Yes	52.8
Acylcarnitines	Hydroxytetradecadienyl-carnitine	C14:2-OH	No	100.0	0.0	0.0	100.0	No	No	100.0	No	100.0	No	100.0	No	100.0
Acylcarnitines	Hexadecanoylcarnitine	C16	Yes	1.3	2.4	0.0	3.7	Yes	Yes	13.1	Yes	0.0	Yes	0.3	Yes	9.9
Acylcarnitines	Hexadecenoylcarnitine	C16:1	Yes	52.5	38.7	0.0	91.2	No	Yes	67.3	Yes	92.7	No	100.0	Yes	81.3
Acylcarnitines	Hydroxyhexadecenoylcarnitine	C16:1-OH	Yes	83.5	14.1	0.0	97.6	No	No	100.0	Yes	85.1	No	100.0	No	100.0

Acylcarnitines	Hexadecadienylcarnitine	C16:2	No	100.0	0.0	0.0	100.0	No	No	100.0	No	100.0	No	100.0	No	100.0
Acylcarnitines	Hydroxyhexadecadienyl-carnitine	C16:2-OH	No	100.0	0.0	0.0	100.0	No	No	100.0	No	100.0	No	100.0	No	100.0
Acylcarnitines	Hydroxyhexadecanoylcarnitine	C16-OH	No	100.0	0.0	0.0	100.0	No	No	100.0	No	100.0	No	100.0	No	100.0
Acylcarnitines	Octadecanoylcarnitine	C18	Yes	1.3	10.4	0.0	11.7	Yes	Yes	21.4	Yes	7.9	Yes	9.4	Yes	15.3
Acylcarnitines	Octadecenoylcarnitine	C18:1	Yes	1.3	1.3	0.0	2.6	Yes	Yes	11.9	Yes	0.0	Yes	0.1	Yes	6.1
Acylcarnitines	Hydroxyoctadecenoylcarnitine	C18:1-OH	No	100.0	0.0	0.0	100.0	No	No	100.0	No	100.0	No	100.0	No	100.0
Acylcarnitines	Octadecadienylcarnitine	C18:2	Yes	1.3	7.9	0.0	9.2	Yes	Yes	39.7	Yes	0.4	Yes	2.7	Yes	16.1
Amino acids	Alanine	Ala	Yes	0.1	0.0	0.0	0.1	Yes	Yes	0.0	Yes	0.0	Yes	0.0	Yes	0.3
Amino acids	Arginine	Arg	Yes	0.1	0.4	0.0	0.5	Yes	Yes	0.0	Yes	0.0	Yes	0.5	Yes	1.1
Amino acids	Asparagine	Asn	Yes	0.1	0.0	0.0	0.1	Yes	Yes	0.0	Yes	0.0	Yes	0.0	Yes	0.3
Amino acids	Aspartate	Asp	Yes	0.1	25.4	0.0	25.5	No	Yes	0.3	Yes	30.8	Yes	25.4	Yes	34.3
Amino acids	Citrulline	Cit	Yes	0.1	0.0	0.0	0.1	Yes	Yes	0.0	Yes	0.0	Yes	0.0	Yes	0.3
Amino acids	Glutamine	Gln	Yes	0.1	0.0	0.0	0.1	Yes	Yes	0.0	Yes	0.0	Yes	0.0	Yes	0.3
Amino acids	Glutamate	Glu	Yes	0.1	0.03	0.0	0.1	Yes	Yes	0.0	Yes	0.2	Yes	0.0	Yes	0.3
Amino acids	Glycine	Gly	Yes	0.1	0.0	0.0	0.1	Yes	Yes	0.0	Yes	0.0	Yes	0.0	Yes	0.3
Amino acids	Histidine	His	Yes	0.1	0.0	0.0	0.1	Yes	Yes	0.0	Yes	0.0	Yes	0.0	Yes	0.3
Amino acids	Isoleucine	Ile	Yes	0.1	0.0	0.0	0.1	Yes	Yes	0.0	Yes	0.0	Yes	0.0	Yes	0.3
Amino acids	Leucine	Leu	Yes	0.1	0.1	0.03	0.2	Yes	Yes	0.3	Yes	0.4	Yes	0.0	Yes	0.3
Amino acids	Lysine	Lys	Yes	0.1	0.0	0.0	0.1	Yes	Yes	0.0	Yes	0.0	Yes	0.0	Yes	0.3
Amino acids	Methionine	Met	Yes	0.1	0.03	0.0	0.1	Yes	Yes	0.0	Yes	0.0	Yes	0.0	Yes	0.5
Amino acids	Ornithine	Orn	Yes	0.1	0.0	0.0	0.1	Yes	Yes	0.0	Yes	0.0	Yes	0.0	Yes	0.3
Amino acids	Phenylalanine	Phe	Yes	0.1	0.0	0.0	0.1	Yes	Yes	0.0	Yes	0.0	Yes	0.0	Yes	0.3
Amino acids	Proline	Pro	Yes	0.1	0.0	0.0	0.1	Yes	Yes	0.0	Yes	0.0	Yes	0.0	Yes	0.3
Amino acids	Serine	Ser	Yes	0.1	0.0	0.0	0.1	Yes	Yes	0.0	Yes	0.0	Yes	0.0	Yes	0.3
Amino acids	Threonine	Thr	Yes	0.1	0.0	0.0	0.1	Yes	Yes	0.0	Yes	0.0	Yes	0.0	Yes	0.3
Amino acids	Tryptophan	Trp	Yes	0.1	0.0	0.0	0.1	Yes	Yes	0.0	Yes	0.0	Yes	0.0	Yes	0.3
Amino acids	Tyrosine	Tyr	Yes	0.1	0.0	0.0	0.1	Yes	Yes	0.0	Yes	0.0	Yes	0.0	Yes	0.3
Amino acids	Valine	Val	Yes	0.1	0.0	0.0	0.1	Yes	Yes	0.0	Yes	0.0	Yes	0.0	Yes	0.3
Amino acids	t4-hydroxyproline	t4-OH-Pro	Yes	11.1	0.0	0.0	11.1	Yes	No	100.0	Yes	0.0	Yes	0.0	Yes	0.3
Biogenic amines	Acetylornithine	Ac-Orn	No	100.0	0.0	0.0	100.0	No	No	100.0	No	100.0	No	100.0	No	100.0
Biogenic amines	alpha-Aminoadipic acid	alpha-AAA	Yes	32.8	38.5	0.0	71.3	No	Yes	0.6	Yes	79.0	Yes	89.0	Yes	59.2
Biogenic amines	Carnosine	Carnosine	No	100.0	0.0	0.0	100.0	No	No	100.0	No	100.0	No	100.0	No	100.0
Biogenic amines	Creatinine	Creatinine	Yes	0.1	0.0	0.0	0.1	Yes	Yes	0.0	Yes	0.0	Yes	0.0	Yes	0.3
Biogenic amines	Histamine	Histamine	No	100.0	0.0	0.0	100.0	No	No	100.0	No	100.0	No	100.0	No	100.0
Biogenic amines	Kynurenine	Kynurenine	Yes	0.1	0.2	0.0	0.3	Yes	Yes	0.0	Yes	0.0	Yes	0.2	Yes	0.9
Biogenic amines	Methioninesulfoxide	Met-SO	Yes	89.0	8.6	0.0	97.6	No	Yes	78.6	No	100.0	No	100.0	No	100.0
Biogenic amines	Nitrotyrosine	Nitro-Tyr	No	100.0	0.0	0.0	100.0	No	No	100.0	No	100.0	No	100.0	No	100.0
Biogenic amines	Phenylethylamine	PEA	No	100.0	0.0	0.0	100.0	No	No	100.0	No	100.0	No	100.0	No	100.0
Biogenic amines	Putrescine	Putrescine	Yes	62.2	27.8	0.0	90.0	No	No	100.0	Yes	77.0	No	100.0	Yes	71.0
Biogenic amines	Sarcosine	Sarcosine	Yes	11.1	16.3	0.0	27.4	No	No	100.0	Yes	20.2	Yes	15.2	Yes	24.9
Biogenic amines	Serotonin	Serotonin	Yes	51.2	32.8	0.0	84.0	No	Yes	3.1	Yes	86.6	No	100.0	Yes	85.2
Biogenic amines	Spermidine	Spermidine	Yes	51.6	43.5	0.0	95.1	No	Yes	79.3	Yes	92.7	No	100.0	Yes	93.2
Biogenic amines	Spermine	Spermine	Yes	51.6	45.3	0.0	96.9	No	Yes	80.8	Yes	97.0	No	100.0	Yes	97.6
Biogenic amines	Taurine	Taurine	Yes	0.1	0.0	0.9	1.0	Yes	Yes	8.3	Yes	0.0	Yes	0.0	Yes	0.3
Biogenic amines	Total dimethylarginine	Total DMA	Yes	89.0	9.5	0.0	98.5	No	Yes	85.9	No	100.0	No	100.0	No	100.0
Biogenic amines	Asymmetric dimethylarginine	ADMA	Yes	2.9	3.6	0.0	6.5	Yes	Yes	32.4	Yes	1.0	Yes	4.4	Yes	2.5
Biogenic amines	Symmetric dimethylarginine	SDMA	Yes	29.5	0.0	0.0	29.5	No	No	100.0	Yes	0.0	Yes	36.0	Yes	0.3
Glycerophospho-	Lysophosphatidylcholine (acyl)	lysoPC a	Yes	89.0	9.9	0.0	98.9	No	Yes	90.2	No	100.0	No	100.0	No	100.0

lipids	C14:0	C14:0														
Glycerophospho-lipids	Lysophosphatidylcholine (acyl) C16:0	lysoPC a C16:0	Yes	0.0	0.0	0.0	0.0	Yes	Yes	0.0	Yes	0.0	Yes	0.0	Yes	0.0
Glycerophospho-lipids	Lysophosphatidylcholine (acyl) C16:1	lysoPC a C16:1	Yes	0.0	0.0	0.0	0.0	Yes	Yes	0.0	Yes	0.0	Yes	0.0	Yes	0.0
Glycerophospho-lipids	Lysophosphatidylcholine (acyl) C17:0	lysoPC a C17:0	Yes	0.0	0.0	0.0	0.0	Yes	Yes	0.0	Yes	0.0	Yes	0.0	Yes	0.0
Glycerophospho-lipids	Lysophosphatidylcholine (acyl) C18:0	lysoPC a C18:0	Yes	0.0	0.0	0.0	0.0	Yes	Yes	0.0	Yes	0.0	Yes	0.0	Yes	0.0
Glycerophospho-lipids	Lysophosphatidylcholine (acyl) C18:1	lysoPC a C18:1	Yes	0.0	0.0	0.0	0.0	Yes	Yes	0.0	Yes	0.0	Yes	0.0	Yes	0.0
Glycerophospho-lipids	Lysophosphatidylcholine (acyl) C18:2	lysoPC a C18:2	Yes	0.0	0.0	0.0	0.0	Yes	Yes	0.0	Yes	0.0	Yes	0.0	Yes	0.0
Glycerophospho-lipids	Lysophosphatidylcholine (acyl) C20:3	lysoPC a C20:3	Yes	0.0	0.0	0.0	0.0	Yes	Yes	0.0	Yes	0.0	Yes	0.0	Yes	0.0
Glycerophospho-lipids	Lysophosphatidylcholine (acyl) C20:4	lysoPC a C20:4	Yes	0.0	0.0	0.0	0.0	Yes	Yes	0.0	Yes	0.0	Yes	0.0	Yes	0.0
Glycerophospho-lipids	Lysophosphatidylcholine (acyl) C24:0	lysoPC a C24:0	Yes	89.0	0.6	0.0	89.6	No	Yes	5.5	No	100.0	No	100.0	No	100.0
Glycerophospho-lipids	Lysophosphatidylcholine (acyl) C26:0	lysoPC a C26:0	No	100.0	0.0	0.0	100.0	No	No	100.0	No	100.0	No	100.0	No	100.0
Glycerophospho-lipids	Lysophosphatidylcholine (acyl) C26:1	lysoPC a C26:1	No	100.0	0.0	0.0	100.0	No	No	100.0	No	100.0	No	100.0	No	100.0
Glycerophospho-lipids	Lysophosphatidylcholine (acyl) C28:0	lysoPC a C28:0	Yes	90.3	2.1	0.0	92.4	No	Yes	31.2	No	100.0	No	100.0	No	100.0
Glycerophospho-lipids	Lysophosphatidylcholine (acyl) C28:1	lysoPC a C28:1	Yes	57.2	0.0	0.0	57.2	No	Yes	0.0	Yes	0.0	Yes	70.2	No	100.0
Glycerophospho-lipids	Phospatidylcholine (diacyl) C24:0	PC aa C24:0	Yes	89.0	6.0	0.0	95.0	No	Yes	54.4	No	100.0	No	100.0	No	100.0
Glycerophospho-lipids	Phospatidylcholine (diacyl) C26:0	PC aa C26:0	No	100.0	0.0	0.0	100.0	No	No	100.0	No	100.0	No	100.0	No	100.0
Glycerophospho-lipids	Phospatidylcholine (diacyl) C28:1	PC aa C28:1	Yes	0.0	0.0	0.0	0.0	Yes	Yes	0.0	Yes	0.0	Yes	0.0	Yes	0.0
Glycerophospho-lipids	Phospatidylcholine (diacyl) C30:0	PC aa C30:0	Yes	0.0	0.0	0.0	0.0	Yes	Yes	0.0	Yes	0.0	Yes	0.0	Yes	0.0
Glycerophospho-lipids	Phospatidylcholine (diacyl) C30:2	PC aa C30:2	No	100.0	0.0	0.0	100.0	No	No	100.0	No	100.0	No	100.0	No	100.0
Glycerophospho-lipids	Phospatidylcholine (diacyl) C32:0	PC aa C32:0	Yes	0.0	0.0	0.0	0.0	Yes	Yes	0.0	Yes	0.0	Yes	0.0	Yes	0.0
Glycerophospho-lipids	Phospatidylcholine (diacyl) C32:1	PC aa C32:1	Yes	0.0	0.0	0.0	0.0	Yes	Yes	0.0	Yes	0.0	Yes	0.0	Yes	0.0
Glycerophospho-lipids	Phospatidylcholine (diacyl) C32:2	PC aa C32:2	Yes	0.0	0.0	0.0	0.0	Yes	Yes	0.0	Yes	0.0	Yes	0.0	Yes	0.0
Glycerophospho-lipids	Phospatidylcholine (diacyl) C32:3	PC aa C32:3	Yes	0.0	0.0	0.0	0.0	Yes	Yes	0.0	Yes	0.0	Yes	0.0	Yes	0.0
Glycerophospho-lipids	Phospatidylcholine (diacyl) C34:1	PC aa C34:1	Yes	0.0	0.0	0.0	0.0	Yes	Yes	0.0	Yes	0.0	Yes	0.0	Yes	0.0
Glycerophospho-lipids	Phospatidylcholine (diacyl) C34:2	PC aa C34:2	Yes	0.0	0.0	0.0	0.0	Yes	Yes	0.0	Yes	0.0	Yes	0.0	Yes	0.0

Glycerophospho-lipids	Phospatidylcholine (diacyl) C34:3	PC aa C34:3	Yes	0.0	0.0	0.0	0.0	Yes	Yes	0.0	Yes	0.0	Yes	0.0	Yes	0.0
Glycerophospho-lipids	Phospatidylcholine (diacyl) C34:4	PC aa C34:4	Yes	0.0	0.0	0.0	0.0	Yes	Yes	0.0	Yes	0.0	Yes	0.0	Yes	0.0
Glycerophospho-lipids	Phospatidylcholine (diacyl) C36:0	PC aa C36:0	Yes	0.0	0.3	0.0	0.3	Yes	Yes	2.1	Yes	0.0	Yes	0.0	Yes	0.2
Glycerophospho-lipids	Phospatidylcholine (diacyl) C36:1	PC aa C36:1	Yes	0.0	0.0	0.0	0.0	Yes	Yes	0.0	Yes	0.0	Yes	0.0	Yes	0.0
Glycerophospho-lipids	Phospatidylcholine (diacyl) C36:2	PC aa C36:2	Yes	0.0	0.0	0.0	0.0	Yes	Yes	0.0	Yes	0.0	Yes	0.0	Yes	0.0
Glycerophospho-lipids	Phospatidylcholine (diacyl) C36:3	PC aa C36:3	Yes	0.0	0.0	0.0	0.0	Yes	Yes	0.0	Yes	0.0	Yes	0.0	Yes	0.0
Glycerophospho-lipids	Phospatidylcholine (diacyl) C36:4	PC aa C36:4	Yes	0.0	0.0	0.0	0.0	Yes	Yes	0.0	Yes	0.0	Yes	0.0	Yes	0.0
Glycerophospho-lipids	Phospatidylcholine (diacyl) C36:5	PC aa C36:5	Yes	0.0	0.0	0.0	0.0	Yes	Yes	0.0	Yes	0.0	Yes	0.0	Yes	0.0
Glycerophospho-lipids	Phospatidylcholine (diacyl) C36:6	PC aa C36:6	Yes	0.0	0.0	0.0	0.0	Yes	Yes	0.0	Yes	0.0	Yes	0.0	Yes	0.0
Glycerophospho-lipids	Phospatidylcholine (diacyl) C38:0	PC aa C38:0	Yes	0.0	0.0	0.0	0.0	Yes	Yes	0.0	Yes	0.0	Yes	0.0	Yes	0.0
Glycerophospho-lipids	Phospatidylcholine (diacyl) C38:1	PC aa C38:1	No	100.0	0.0	0.0	100.0	No	No	100.0	No	100.0	No	100.0	No	100.0
Glycerophospho-lipids	Phospatidylcholine (diacyl) C38:3	PC aa C38:3	Yes	0.0	0.0	0.0	0.0	Yes	Yes	0.0	Yes	0.0	Yes	0.0	Yes	0.0
Glycerophospho-lipids	Phospatidylcholine (diacyl) C38:4	PC aa C38:4	Yes	0.0	0.0	0.0	0.0	Yes	Yes	0.0	Yes	0.0	Yes	0.0	Yes	0.0
Glycerophospho-lipids	Phospatidylcholine (diacyl) C38:5	PC aa C38:5	Yes	0.0	0.0	0.0	0.0	Yes	Yes	0.0	Yes	0.0	Yes	0.0	Yes	0.0
Glycerophospho-lipids	Phospatidylcholine (diacyl) C38:6	PC aa C38:6	Yes	0.0	0.0	0.0	0.0	Yes	Yes	0.0	Yes	0.0	Yes	0.0	Yes	0.0
Glycerophospho-lipids	Phospatidylcholine (diacyl) C40:1	PC aa C40:1	Yes	35.9	38.1	0.0	74.0	No	Yes	25.1	Yes	66.6	Yes	92.7	Yes	60.3
Glycerophospho-lipids	Phospatidylcholine (diacyl) C40:2	PC aa C40:2	Yes	3.1	0.0	0.0	3.1	Yes	Yes	0.0	Yes	0.0	Yes	6.1	Yes	0.0
Glycerophospho-lipids	Phospatidylcholine (diacyl) C40:3	PC aa C40:3	Yes	3.1	0.0	0.0	3.1	Yes	Yes	0.0	Yes	0.0	Yes	6.1	Yes	0.0
Glycerophospho-lipids	Phospatidylcholine (diacyl) C40:4	PC aa C40:4	Yes	0.0	0.0	0.0	0.0	Yes	Yes	0.0	Yes	0.0	Yes	0.0	Yes	0.0
Glycerophospho-lipids	Phospatidylcholine (diacyl) C40:5	PC aa C40:5	Yes	0.0	0.0	0.0	0.0	Yes	Yes	0.0	Yes	0.0	Yes	0.0	Yes	0.0
Glycerophospho-lipids	Phospatidylcholine (diacyl) C40:6	PC aa C40:6	Yes	0.0	0.0	0.0	0.0	Yes	Yes	0.0	Yes	0.0	Yes	0.0	Yes	0.0
Glycerophospho-lipids	Phospatidylcholine (diacyl) C42:0	PC aa C42:0	Yes	0.0	0.0	0.0	0.0	Yes	Yes	0.0	Yes	0.0	Yes	0.0	Yes	0.0
Glycerophospho-lipids	Phospatidylcholine (diacyl) C42:1	PC aa C42:1	Yes	0.0	0.0	0.0	0.0	Yes	Yes	0.0	Yes	0.0	Yes	0.0	Yes	0.0
Glycerophospho-lipids	Phospatidylcholine (diacyl) C42:2	PC aa C42:2	Yes	0.0	0.1	0.0	0.1	Yes	Yes	0.0	Yes	0.0	Yes	0.3	Yes	0.0
Glycerophospho-	Phospatidylcholine (diacyl)	PC aa C42:4	Yes	3.1	0.0	0.0	3.1	Yes	Yes	0.0	Yes	0.0	Yes	6.1	Yes	0.0

lipids	C42:4															
Glycerophospho-lipids	Phospatidylcholine (diacyl) C42:5	PC aa C42:5	Yes	3.1	0.0	0.0	3.1	Yes	Yes	0.0	Yes	0.0	Yes	6.1	Yes	0.0
Glycerophospho-lipids	Phospatidylcholine (diacyl) C42:6	PC aa C42:6	Yes	3.1	4.5	0.0	7.6	Yes	Yes	19.0	Yes	2.7	Yes	8.7	Yes	3.2
Glycerophospho-lipids	Phospatidylcholine (acyl-alkyl) C30:0	PC ae C30:0	Yes	3.1	0.5	0.0	3.6	Yes	Yes	0.0	Yes	0.4	Yes	7.0	Yes	0.0
Glycerophospho-lipids	Phospatidylcholine (acyl-alkyl) C30:1	PC ae C30:1	No	100.0	0.0	0.0	100.0	No	No	100.0	No	100.0	No	100.0	No	100.0
Glycerophospho-lipids	Phospatidylcholine (acyl-alkyl) C30:2	PC ae C30:2	Yes	3.1	0.0	0.0	3.1	Yes	Yes	0.0	Yes	0.0	Yes	6.1	Yes	0.0
Glycerophospho-lipids	Phospatidylcholine (acyl-alkyl) C32:1	PC ae C32:1	Yes	0.0	0.0	0.0	0.0	Yes	Yes	0.0	Yes	0.0	Yes	0.0	Yes	0.0
Glycerophospho-lipids	Phospatidylcholine (acyl-alkyl) C32:2	PC ae C32:2	Yes	0.0	0.0	0.0	0.0	Yes	Yes	0.0	Yes	0.0	Yes	0.0	Yes	0.0
Glycerophospho-lipids	Phospatidylcholine (acyl-alkyl) C34:0	PC ae C34:0	Yes	0.0	0.0	0.0	0.0	Yes	Yes	0.0	Yes	0.0	Yes	0.0	Yes	0.0
Glycerophospho-lipids	Phospatidylcholine (acyl-alkyl) C34:1	PC ae C34:1	Yes	0.0	0.0	0.0	0.0	Yes	Yes	0.0	Yes	0.0	Yes	0.0	Yes	0.0
Glycerophospho-lipids	Phospatidylcholine (acyl-alkyl) C34:2	PC ae C34:2	Yes	0.0	0.0	0.0	0.0	Yes	Yes	0.0	Yes	0.0	Yes	0.0	Yes	0.0
Glycerophospho-lipids	Phospatidylcholine (acyl-alkyl) C34:3	PC ae C34:3	Yes	0.0	0.0	0.0	0.0	Yes	Yes	0.0	Yes	0.0	Yes	0.0	Yes	0.0
Glycerophospho-lipids	Phospatidylcholine (acyl-alkyl) C36:0	PC ae C36:0	Yes	3.1	0.0	0.0	3.1	Yes	Yes	0.0	Yes	0.0	Yes	6.1	Yes	0.0
Glycerophospho-lipids	Phospatidylcholine (acyl-alkyl) C36:1	PC ae C36:1	Yes	0.0	0.0	0.0	0.0	Yes	Yes	0.0	Yes	0.0	Yes	0.0	Yes	0.0
Glycerophospho-lipids	Phospatidylcholine (acyl-alkyl) C36:2	PC ae C36:2	Yes	0.0	0.0	0.0	0.0	Yes	Yes	0.0	Yes	0.0	Yes	0.0	Yes	0.0
Glycerophospho-lipids	Phospatidylcholine (acyl-alkyl) C36:3	PC ae C36:3	Yes	0.0	0.0	0.0	0.0	Yes	Yes	0.0	Yes	0.0	Yes	0.0	Yes	0.0
Glycerophospho-lipids	Phospatidylcholine (acyl-alkyl) C36:4	PC ae C36:4	Yes	0.0	0.0	0.0	0.0	Yes	Yes	0.0	Yes	0.0	Yes	0.0	Yes	0.0
Glycerophospho-lipids	Phospatidylcholine (acyl-alkyl) C36:5	PC ae C36:5	Yes	0.0	0.0	0.0	0.0	Yes	Yes	0.0	Yes	0.0	Yes	0.0	Yes	0.0
Glycerophospho-lipids	Phospatidylcholine (acyl-alkyl) C38:0	PC ae C38:0	Yes	18.4	0.0	0.0	18.4	Yes	Yes	0.0	Yes	0.0	Yes	36.0	Yes	0.0
Glycerophospho-lipids	Phospatidylcholine (acyl-alkyl) C38:1	PC ae C38:1	No	100.0	0.0	0.0	100.0	No	No	100.0	No	100.0	No	100.0	No	100.0
Glycerophospho-lipids	Phospatidylcholine (acyl-alkyl) C38:2	PC ae C38:2	Yes	0.0	0.0	0.0	0.0	Yes	Yes	0.0	Yes	0.0	Yes	0.0	Yes	0.0
Glycerophospho-lipids	Phospatidylcholine (acyl-alkyl) C38:3	PC ae C38:3	Yes	0.0	0.0	0.0	0.0	Yes	Yes	0.0	Yes	0.0	Yes	0.0	Yes	0.0
Glycerophospho-lipids	Phospatidylcholine (acyl-alkyl) C38:4	PC ae C38:4	Yes	0.0	0.0	0.0	0.0	Yes	Yes	0.0	Yes	0.0	Yes	0.0	Yes	0.0
Glycerophospho-lipids	Phospatidylcholine (acyl-alkyl) C38:5	PC ae C38:5	Yes	0.0	0.0	0.0	0.0	Yes	Yes	0.0	Yes	0.0	Yes	0.0	Yes	0.0
Glycerophospho-lipids	Phospatidylcholine (acyl-alkyl) C38:6	PC ae C38:6	Yes	0.0	0.0	0.0	0.0	Yes	Yes	0.0	Yes	0.0	Yes	0.0	Yes	0.0

Glycerophospho-lipids	Phospatidylcholine (acyl-alkyl) C40:1	PC ae C40:1	Yes	0.0	0.0	0.0	0.0	Yes	Yes	0.0	Yes	0.0	Yes	0.0	Yes	0.0
Glycerophospho-lipids	Phospatidylcholine (acyl-alkyl) C40:2	PC ae C40:2	Yes	0.0	0.0	0.0	0.0	Yes	Yes	0.0	Yes	0.0	Yes	0.0	Yes	0.0
Glycerophospho-lipids	Phospatidylcholine (acyl-alkyl) C40:3	PC ae C40:3	Yes	3.1	0.0	0.0	3.1	Yes	Yes	0.0	Yes	0.0	Yes	6.1	Yes	0.0
Glycerophospho-lipids	Phospatidylcholine (acyl-alkyl) C40:4	PC ae C40:4	Yes	0.0	0.0	0.0	0.0	Yes	Yes	0.0	Yes	0.0	Yes	0.0	Yes	0.0
Glycerophospho-lipids	Phospatidylcholine (acyl-alkyl) C40:5	PC ae C40:5	Yes	0.0	0.0	0.0	0.0	Yes	Yes	0.0	Yes	0.0	Yes	0.0	Yes	0.0
Glycerophospho-lipids	Phospatidylcholine (acyl-alkyl) C40:6	PC ae C40:6	Yes	0.0	0.0	0.0	0.0	Yes	Yes	0.0	Yes	0.0	Yes	0.0	Yes	0.0
Glycerophospho-lipids	Phospatidylcholine (acyl-alkyl) C42:0	PC ae C42:0	No	100.0	0.0	0.0	100.0	No	No	100.0	No	100.0	No	100.0	No	100.0
Glycerophospho-lipids	Phospatidylcholine (acyl-alkyl) C42:1	PC ae C42:1	Yes	2.1	0.7	0.0	2.8	Yes	Yes	0.0	Yes	0.4	Yes	4.6	Yes	1.6
Glycerophospho-lipids	Phospatidylcholine (acyl-alkyl) C42:2	PC ae C42:2	Yes	0.0	0.0	0.0	0.0	Yes	Yes	0.0	Yes	0.0	Yes	0.0	Yes	0.0
Glycerophospho-lipids	Phospatidylcholine (acyl-alkyl) C42:3	PC ae C42:3	Yes	0.0	0.0	0.0	0.0	Yes	Yes	0.0	Yes	0.0	Yes	0.0	Yes	0.0
Glycerophospho-lipids	Phospatidylcholine (acyl-alkyl) C42:4	PC ae C42:4	Yes	0.0	0.0	0.0	0.0	Yes	Yes	0.0	Yes	0.0	Yes	0.0	Yes	0.0
Glycerophospho-lipids	Phospatidylcholine (acyl-alkyl) C42:5	PC ae C42:5	Yes	0.0	0.0	0.0	0.0	Yes	Yes	0.0	Yes	0.0	Yes	0.0	Yes	0.0
Glycerophospho-lipids	Phospatidylcholine (acyl-alkyl) C44:3	PC ae C44:3	Yes	34.8	0.3	0.0	35.1	No	Yes	0.0	Yes	0.8	Yes	68.4	Yes	0.0
Glycerophospho-lipids	Phospatidylcholine (acyl-alkyl) C44:4	PC ae C44:4	Yes	0.0	0.1	0.0	0.1	Yes	Yes	0.0	Yes	0.0	Yes	0.1	Yes	0.0
Glycerophospho-lipids	Phospatidylcholine (acyl-alkyl) C44:5	PC ae C44:5	Yes	0.0	0.0	0.0	0.0	Yes	Yes	0.0	Yes	0.0	Yes	0.0	Yes	0.0
Glycerophospho-lipids	Phospatidylcholine (acyl-alkyl) C44:6	PC ae C44:6	Yes	0.0	0.0	0.0	0.0	Yes	Yes	0.0	Yes	0.0	Yes	0.0	Yes	0.0
Sphingolipids	Hydroxysphingomyeline C14:1	SM (OH) C14:1	Yes	0.0	0.0	0.0	0.0	Yes	Yes	0.0	Yes	0.0	Yes	0.0	Yes	0.0
Sphingolipids	Hydroxysphingomyeline C16:1	SM (OH) C16:1	Yes	0.0	0.0	0.0	0.0	Yes	Yes	0.0	Yes	0.0	Yes	0.0	Yes	0.0
Sphingolipids	Hydroxysphingomyeline C22:1	SM (OH) C22:1	Yes	0.0	0.0	0.0	0.0	Yes	Yes	0.0	Yes	0.0	Yes	0.0	Yes	0.0
Sphingolipids	Hydroxysphingomyeline C22:2	SM (OH) C22:2	Yes	0.0	0.0	0.0	0.0	Yes	Yes	0.0	Yes	0.0	Yes	0.0	Yes	0.0
Sphingolipids	Hydroxysphingomyeline C24:1	SM (OH) C24:1	Yes	0.0	0.0	0.0	0.0	Yes	Yes	0.0	Yes	0.0	Yes	0.0	Yes	0.0
Sphingolipids	Sphingomyeline C16:0	SM C16:0	Yes	0.0	0.0	0.0	0.0	Yes	Yes	0.0	Yes	0.0	Yes	0.0	Yes	0.0
Sphingolipids	Sphingomyeline C16:1	SM C16:1	Yes	0.0	0.0	0.0	0.0	Yes	Yes	0.0	Yes	0.0	Yes	0.0	Yes	0.0
Sphingolipids	Sphingomyeline C18:0	SM C18:0	Yes	0.0	0.0	0.0	0.0	Yes	Yes	0.0	Yes	0.0	Yes	0.0	Yes	0.0
Sphingolipids	Sphingomyeline C18:1	SM C18:1	Yes	0.0	0.0	0.0	0.0	Yes	Yes	0.0	Yes	0.0	Yes	0.0	Yes	0.0
Sphingolipids	Sphingomyeline C20:2	SM C20:2	Yes	0.0	0.0	0.0	0.0	Yes	Yes	0.0	Yes	0.0	Yes	0.0	Yes	0.0
Sphingolipids	Sphingomyeline C22:3	SM C22:3	No	100.0	0.0	0.0	100.0	No	No	100.0	No	100.0	No	100.0	No	100.0
Sphingolipids	Sphingomyeline C24:0	SM C24:0	Yes	0.0	0.0	0.0	0.0	Yes	Yes	0.0	Yes	0.0	Yes	0.0	Yes	0.0

Sphingolipids	Sphingomyeline C24:1	SM C24:1	Yes	0.0	0.0	0.0	0.0	Yes	Yes	0.0	Yes	0.0	Yes	0.0	Yes	0.0
Sphingolipids	Sphingomyeline C26:0	SM C26:0	Yes	51.1	0.0	0.0	51.1	No	Yes	0.0	Yes	0.0	Yes	100.0	Yes	0.0
Sphingolipids	Sphingomyeline C26:1	SM C26:1	Yes	32.8	0.0	0.0	32.8	No	Yes	0.0	Yes	0.0	Yes	64.0	Yes	0.0
Hexoses	Hexoses	H1	Yes	0.0	2.6	0.0	2.6	Yes	Yes	0.0	Yes	2.7	Yes	3.0	No	3.0
Total number		186	157					123	147		147		138		144	

Abbreviations: LOD, limit of detection; LOQ, limit of quantification; n, number; perc., percentage.

Supplementary Table 2. Participant characteristics by sub-study (nested case-control study the controls were derived from) and in the total dataset

	Colorectal cancer controls (n=491, 16.5%)	Kidney cancer controls (n=635, 21.4%)	Hepatobiliary cancer controls (n=327, 11.0%)	Prostate cancer controls (n=1,521, 51.1%)	Total dataset (n=2,974, 100%)
Age, *mean (SD)*	56.5 (8.0)	55.9 (8.3)	59.6 (7.4)	59.7 (7.0)	58.3 (7.7)
Sex, *n (%)*					
Men	188 (38.3)	337 (53.1)	185 (56.6)	1,521 (100.0)	2,231 (75.0)
Women	303 (61.7)	298 (46.9)	142 (43.4)	0 (0.0)	743 (25.0)
Education level, *n (%)*[a]					
None/primary	262 (54.4)	271 (44.2)	152 (47.8)	614 (42.6)	1,299 (1,299)
Secondary	65 (13.5)	90 (14.7)	37 (11.6)	161 (11.2)	353 (353)
Technical/professional	81 (16.8)	135 (22.0)	74 (23.3)	359 (24.9)	649 (649)
University or higher	74 (15.4)	117 (19.1)	55 (17.3)	306 (21.3)	552 (552)
Alcohol intake in men (g/day), *median (5th, 95th perc)*	18.5 (0.1, 60.8)	15.5 (0.0, 73.9)	12.8 (0.1, 57.1)	13.2 (0.0, 63.4)	13.7 (0.0, 64.0)
Alcohol intake in women (g/day), *median (5th, 95th perc)*	2.0 (0.0, 25.3)	3.3 (0.0, 29.5)	3.3 (0.0, 24.7)	NA NA	2.8 (0.0, 25.7)
Categories of alcohol intake, *n (%)*					
Non-drinkers (<0.1 g/day)	97 (19.8)	85 (13.4)	40 (12.2)	123 (8.1)	345 (11.6)
Light drinkers (0.1-4.9 g/day)	136 (27.7)	184 (29.0)	97 (29.7)	309 (20.3)	726 (24.4)
Moderate drinkers (5-39.9 g/day)	216 (44.0)	300 (47.2)	163 (49.9)	842 (55.4)	1,521 (51.1)
Heavy drinkers (≥40 g/day)	42 (8.6)	66 (10.4)	27 (8.3)	247 (16.2)	382 (12.8)
Body mass index (kg/m^2), *mean (SD)*	26.6 (3.7)	26.7 (4.0)	27.1 (4.1)	26.9 (3.5)	26.8 (26.8)
Physical activity, *n (%)*[b]					
Inactive	154 (31.4)	171 (27.1)	108 (33.2)	390 (26.2)	823 (823)
Moderately inactive	193 (39.4)	195 (31.0)	103 (31.7)	489 (32.9)	980 (980)
Moderately active	80 (16.3)	147 (23.3)	61 (18.8)	332 (22.3)	620 (620)
Active	63 (12.9)	117 (18.6)	53 (16.3)	277 (18.6)	510 (510)
Smoking status, *n (%)*[c]					
Current smoker	92 (18.9)	131 (20.9)	68 (21.2)	373 (24.9)	664 (22.7)
Former smoker	136 (27.9)	210 (33.5)	105 (32.7)	678 (45.3)	1,129 (38.5)
Never smoker	260 (53.3)	286 (45.6)	148 (46.1)	445 (29.8)	1,139 (38.9)
Meat intake (g/day), *median (5th, 95th perc)*	104.4 (32.9, 198.7)	101.7 (30.2, 234.3)	101.1 (35.4, 209.2)	111.0 (36.2, 232.5)	106.6 (32.9, 222.9)
Fish intake (g/day), *median (5th, 95th perc)*	27.1 (1.6, 110.4)	26.3 (3.0, 110.9)	28.6 (3.8, 93.1)	30.4 (2.5, 119.8)	28.8 (2.5, 113.2)
Energy intake (kcal/day), *median (5th, 95th perc)*	2120.1 (1292.2, 3355.0)	2037.3 (1304.3, 3413.5)	2094.8 (1290.7, 3334.1)	2300.0 (1390.6, 3555.7)	2188.3 (1327.7, 3480.4)
Fasting status, *n (%)*[d]					
≥6 hours	375 (76.4)	223 (35.5)	86 (30.8)	482 (32.1)	1,166 (40.2)
3-5.9 hours	92 (18.7)	121 (19.3)	59 (21.2)	292 (19.5)	564 (19.5)
<3 hours	24 (4.9)	284 (45.2)	134 (48.0)	727 (48.4)	1,169 (40.3)
Time between blood collection and metabolomics analysis (years), *median (5th, 95th perc)*	21.1 (18.2, 22.8)	21.0 (18.2, 23.2)	17.3 (14.6, 20.0)	20.7 (18.3, 22.9)	20.6 (16.9, 22.9)
Country, *n (%)*					
Denmark	0 (0.0)	0 (0.0)	51 (15.6)	0 (0.0)	51 (1.7)
France	24 (4.9)	16 (2.5)	0 (0.0)	0 (0.0)	40 (1.3)
Germany	41 (8.4)	153 (24.1)	63 (19.3)	347 (22.8)	604 (20.3)
Greece	23 (4.7)	28 (4.4)	31 (9.5)	36 (2.4)	118 (4.0)
Italy	204 (41.6)	129 (20.3)	52 (15.9)	273 (18.0)	658 (22.1)
Norway	0 (0.0)	14 (2.2)	0 (0.0)	0 (0.0)	14 (0.5)
Spain	114 (23.2)	123 (19.4)	31 (9.5)	303 (19.9)	571 (19.2)
Sweden	0 (0.0)	0 (0.0)	48 (14.7)	0 (0.0)	48 (1.6)

The Netherlands	40	(8.2)	68	(10.7)	16	(4.9)	72	(4.7)	196	(6.6)
United Kingdom	45	(9.2)	104	(16.4)	35	(10.7)	490	(32.2)	674	(22.7)

Abbreviations: n, number; NA, not applicable (no women among prostate cancer controls), perc, percentile; SD, standard deviation.

[a] Data missing for 121 participants (99 men and 22 women).

[b] Cambridge physical activity index: cross-classification of the level of occupational activity with cycling and sports activities and recreational activities(1); data missing for 41 participants (36 men and 5 women)

[c] Data missing for 42 participants (3 from colorectal cancer, 8 from kidney cancer, 6 from hepatobiliary cancer, and 25 from prostate cancer case-control study).

[d] Data missing for 75 participants (24 from colorectal cancer [4.9%], 7 from kidney cancer [1.1%], 48 from hepatobiliary cancer [14.7], and 20 from prostate cancer case-control study [1.3%])).

Supplementary Table 3. Summary statistics of untransformed concentrations (µM) of metabolites included in the current analysis (n=123)

	n	Mean	SD	Median	5th perc	95th perc	Min	Max
**Acylcarnitines**								
Acylcarnitine C0	2,974	32.73461	7.389134	32.1	22.2	45.9	8.38	69.985
Acylcarnitine C14:1	2,935	0.060699	0.030026	0.055	0.027	0.111	0.008	0.391
Acylcarnitine C16	2,935	0.099341	0.036127	0.093	0.057	0.157	0.031	0.499
Acylcarnitine C18	2,935	0.049809	0.018678	0.047	0.025	0.084	0.012	0.278
Acylcarnitine C18:1	2,935	0.115449	0.056586	0.101	0.06	0.216	0.026	0.615
Acylcarnitine C18:2	2,935	0.052078	0.035809	0.043	0.021	0.113	0.006	0.448
Acylcarnitine C2	2,974	5.16648	1.914669	4.79	2.87	8.82	1.58	19.792
Acylcarnitine C3	2,935	0.351729	0.117688	0.333	0.203	0.579	0.086	0.996
Acylcarnitine C4	2,790	0.209533	0.100354	0.187	0.113	0.375	0.05	2.11
Acylcarnitine C5	2,756	0.145771	0.051897	0.141	0.061	0.232	0.029	0.528
**Amino acids**								
Alanine	2,972	346.0491	92.84259	333.5	217	517	136	776
Arginine	2,972	59.87138	29.16129	56.1	17.8	115	2.5	249
Asparagine	2,972	42.23913	11.1565	39.9	29.2	62.5	17.2	122
Citrulline	2,972	30.58997	9.411851	29.2	18.3	47.4	6.21	131
Glutamine	2,972	595.7638	94.65734	594	445	754	271	985
Glutamate	2,972	61.75424	43.80076	49.95	21.9	144	5	513
Glycine	2,972	196.9827	65.91607	181	124	335	86.5	632
Histidine	2,972	72.4718	13.63417	70.2	54.8	97.6	31.8	173
Isoleucine	2,972	70.21726	22.61768	66.05	42.9	112	23.5	267.1
Leucine	2,971	130.3946	37.09274	123	82.4	199	25	383
Lysine	2,972	190.1272	44.11736	185	128	267	67.5	424
Methionine	2,972	20.34027	6.056021	19.3	12.9	31.5	2.5	69.3
Ornithine	2,972	86.15192	32.37985	80.25	46.7	149	23.9	319
Phenylalanine	2,972	59.42937	13.428	56.8	42.6	85.1	28.5	155
Proline	2,972	188.2811	59.69239	179	109	298	57	646
Serine	2,972	92.26396	28.44239	86.95	59.4	146	33.8	329
Threonine	2,972	109.053	27.08418	106	72	157	40.8	254
Tryptophan	2,972	54.13321	11.2671	52.8	38.1	73.9	21	114
Tyrosine	2,972	64.9753	16.65244	62.5	42.9	96.2	20.8	181
Valine	2,972	210.035	46.20568	205	145	293	100	450
t4-hydroxyproline	2,645	9.042219	4.810462	7.84	4.19	17.9	2.43	62.9
**Biogenic amines**								
ADMA	2,888	0.400684	0.109534	0.393	0.258	0.589	0.125	0.951
Creatine	2,972	67.89186	18.30918	66.55	44.7	94.8	29.5	513
Kynurenine	2,972	2.217786	0.671095	2.095	1.37	3.44	0.5	6.99
Taurine	2,972	57.2146	30.62363	48	30.1	120	19.6	200
**Glycerophospholipids**								
LysoPC a C16:0	2,974	110.6712	37.07529	102	68.8	187.694	45.8	321.972
LysoPC a C16:1	2,974	3.202193	1.318059	2.95	1.64	5.621	0.9	18.14
LysoPC a C17:0	2,974	2.174364	0.948001	1.98	1.08	4.062	0.549	7.285
LysoPC a C18:0	2,974	35.91004	14.50811	32.05	20.7	66.8	9.74	146.171
LysoPC a C18:1	2,974	24.52513	9.907317	22.4	13	43.2	7.51	94.262
LysoPC a C18:2	2,974	34.70049	14.16139	31.9	17.9	60.828	7.49	129.118
LysoPC a C20:3	2,974	2.456442	0.974579	2.27	1.24	4.35	0.413	9.243
LysoPC a C20:4	2,974	6.606712	2.508269	6.18	3.41	11.377	1.89	21.659

PC aa C28:1	2,974	2.48236	0.807288	2.36	1.38	3.97	0.573	7.74
PC aa C30:0	2,974	3.471262	1.396116	3.225	1.71	6.16	0.903	12.604
PC aa C32:0	2,974	19.52583	4.882344	19	12.5	28.5	8.17	42.8
PC aa C32:1	2,974	23.27354	13.97404	20.1	7.93	50.1	3.49	123
PC aa C32:2	2,974	5.912849	2.318035	5.62	2.64	10.2	0.333	16.1
PC aa C32:3	2,974	0.773056	0.246226	0.727	0.452	1.24	0.299	2.24
PC aa C34:1	2,974	251.3815	80.18321	236	150	402	76.5	716
PC aa C34:2	2,974	468.2106	165.9334	493.5	212.683	719	147.605	957
PC aa C34:3	2,974	20.58805	7.311645	19.4	10.7	33.614	4.77	67.909
PC aa C34:4	2,974	2.345382	0.87204	2.2	1.17	4.01	0.224	7.415
PC aa C36:0	2,974	2.741276	1.151084	2.57	1.25	4.68	0.101	12.7
PC aa C36:1	2,974	52.63843	16.50842	49.9	31	83.508	16.3	150.315
PC aa C36:2	2,974	274.449	67.76033	271	177	391	78.6	537
PC aa C36:3	2,974	159.0656	37.48239	156	105	225	42.1	336
PC aa C36:4	2,974	206.3662	56.28536	197	130	311	58.2	519
PC aa C36:5	2,974	30.82985	19.38193	26.0705	11.5	68.636	3.58	240
PC aa C36:6	2,974	1.266968	0.563601	1.16	0.59	2.316	0.193	6.52
PC aa C38:0	2,974	2.997285	0.986715	2.83	1.72	4.8	1	8.669
PC aa C38:3	2,974	46.52686	13.09129	44.8	28.2	70.913	14.6	112.289
PC aa C38:4	2,974	99.32027	27.23202	96.1	60.7	149	21.4	236
PC aa C38:5	2,974	51.2289	14.55407	49.2	31.2	77.6	17.6	150
PC aa C38:6	2,974	88.55192	28.45438	84.2	49.9	142	24	230.756
PC aa C40:2	2,881	0.276511	0.110369	0.257	0.169	0.457	0.119	3.02
PC aa C40:3	2,881	0.461409	0.169306	0.422	0.281	0.77	0.139	2.74
PC aa C40:4	2,974	2.654658	0.813049	2.528	1.59	4.1	0.529	8.959
PC aa C40:5	2,974	6.590204	2.266583	6.23	3.58	10.642	2.19	30.1
PC aa C40:6	2,974	26.02427	9.140255	24.8	13.9	42.9	6.8	89.466
PC aa C42:0	2,974	0.376757	0.156556	0.341	0.19	0.679	0.123	1.469
PC aa C42:1	2,974	0.190883	0.079939	0.174	0.098	0.348	0.059	0.722
PC aa C42:2	2,974	0.169405	0.063685	0.158	0.097	0.284	0.039	0.749
PC aa C42:4	2,881	0.108635	0.034551	0.102	0.065	0.172	0.026	0.527
PC aa C42:5	2,881	0.234329	0.091164	0.215	0.128	0.407	0.066	1.09
PC aa C42:6	2,881	0.365058	0.137807	0.337	0.208	0.632	0.092	1.472
PC ae C30:0	2,881	0.298554	0.104036	0.284	0.157	0.489	0.05	0.961
PC ae C30:2	2,881	0.080207	0.03334	0.074	0.042	0.141	0.023	0.461
PC ae C32:1	2,974	3.675022	0.910912	3.59	2.37	5.283	1.36	9.031
PC ae C32:2	2,974	1.049277	0.266466	1.0205	0.665	1.53	0.396	2.382
PC ae C34:0	2,974	2.005947	0.639761	1.92	1.15	3.17	0.587	6.23
PC ae C34:1	2,974	12.69445	3.334533	12.3	7.98	18.7	4.68	28.9
PC ae C34:2	2,974	15.37617	4.099373	14.954	9.5	22.9	5.36	39.151
PC ae C34:3	2,974	10.26412	3.081846	9.8725	5.93	15.9	3.47	29.731
PC ae C36:0	2,881	0.971688	0.308394	0.917	0.587	1.501	0.346	3.86
PC ae C36:1	2,974	9.70715	3.086505	9.27	5.78	14.957	3.45	72.3
PC ae C36:2	2,974	17.87198	4.853692	17.249	10.9	26.3	6.2	45.4
PC ae C36:3	2,974	9.347051	2.313299	9.11	6.05	13.5	3.44	22.635
PC ae C36:4	2,974	19.44095	5.55142	18.7	11.9	29.4	7.33	50.556
PC ae C36:5	2,974	13.52497	3.936125	13.1	7.93	20.6	4.62	31.4
PC ae C38:0	2,427	2.371954	0.827715	2.22	1.35	3.91	0.776	6.41
PC ae C38:2	2,974	2.28626	0.768703	2.17	1.43	3.46	0.948	25.1

PC ae C38:3	2,974	4.21808	1.156773	4.08	2.62	6.12	1.71	22.7
PC ae C38:4	2,974	12.90652	3.18535	12.543	8.41	18.4	4.84	38.1
PC ae C38:5	2,974	17.72872	4.2043	17.3	11.7	25.3	7.71	41.8
PC ae C38:6	2,974	8.03502	2.288435	7.71	4.9	12.1	3.03	23.514
PC ae C40:1	2,974	1.195129	0.31475	1.16	0.77	1.736	0.409	4.62
PC ae C40:2	2,974	1.541132	0.464061	1.47	0.921	2.38	0.539	6.01
PC ae C40:3	2,881	0.818917	0.245829	0.789	0.537	1.2	0.363	7.84
PC ae C40:4	2,974	1.951638	0.437764	1.91	1.35	2.73	0.762	7.17
PC ae C40:5	2,974	3.010946	0.696726	2.937	2.06	4.232	1.39	8.294
PC ae C40:6	2,974	4.359259	1.206708	4.18	2.71	6.523	1.79	11.744
PC ae C42:1	2,913	0.308291	0.079129	0.298	0.213	0.436	0.078	1.88
PC ae C42:2	2,974	0.414051	0.11783	0.395	0.258	0.633	0.168	1.55
PC ae C42:3	2,974	0.5581	0.158788	0.539	0.339	0.846	0.205	1.76
PC ae C42:4	2,974	0.560443	0.160301	0.537	0.345	0.85	0.212	1.811
PC ae C42:5	2,974	1.475112	0.34811	1.43	0.996	2.09	0.665	3.878
PC ae C44:4	2,974	0.2082	0.073364	0.193	0.116	0.352	0.039	0.709
PC ae C44:5	2,974	0.867868	0.324382	0.803	0.474	1.481	0.238	2.917
PC ae C44:6	2,974	0.709821	0.24342	0.669	0.402	1.153	0.249	2.316
**Sphingolipids**								
SM C16:0	2,974	78.57645	25.29808	71.1	48.8	129.135	23.8	181.565
SM C16:1	2,974	11.11282	4.202389	9.72	6.58	19.562	3.58	33.319
SM C18:0	2,974	14.52333	6.613006	12.2585	7.62	28.555	3.72	48.362
SM C18:1	2,974	7.102597	3.262151	5.97	3.69	13.8	2.15	26.78
SM C20:2	2,974	0.3769	0.292159	0.294	0.11	0.924	0.036	4.03
SM C24:0	2,974	9.350157	4.563673	7.525	4.69	18.3	2.47	34.6
SM C24:1	2,974	19.9373	10.00519	15.9	9.58	39.55	4.97	71.3
SM(OH) C14:1	2,974	4.542725	1.863094	4.08	2.34	8.249	1.13	14.151
SM(OH) C16:1	2,974	2.299296	1.053568	1.97	1.14	4.47	0.552	7.327
SM(OH) C22:1	2,974	6.851216	3.262027	5.59	3.39	13.592	1.46	20.798
SM(OH) C22:2	2,974	5.240825	2.565633	4.33	2.58	10.412	1.44	16.456
SM(OH) C24:1	2,974	0.553493	0.311254	0.43	0.236	1.19	0.09	1.86
**Hexoses**								
Hexoses	2,974	4113.384	1542.154	4004.5	2178	6124	750	20297

Abbreviations: min, minimum; max, maximum; n, number; perc, percentile; SD, standard deviation.
For an explanation of abbreviated metabolite names, see Supplementary Table 1.

Supplementary Table 4. Results of discovery and replication analysis on associations of alcohol intake with concentrations of metabolites included in the current analysis (n=123)[a]

	Discovery analysis (n=1,983)[b]						Replication analysis (n=991)[b]					
Metabolite	n	β	SE	p-value	FDR q-value[c]	R^2	n	β	SE	p-value	Bonf.-adj. p-value[d]	R^2
**Acylcarnitines**												
Acylcarnitine C0	1,983	0.05	0.02	0.003939	**0.008354**	0.06	991	0.02	0.02	0.474501	1	0.07
Acylcarnitine C14:1	1,956	0.05	0.02	0.002151	**0.004725**	0.09	979	0.08	0.02	0.000653	**0.047001**	0.14
Acylcarnitine C16	1,956	0.11	0.02	<1E-12	**<1E-12**	0.15	979	0.13	0.02	4.38E-09	**3.15E-07**	0.20
Acylcarnitine C18	1,956	0.06	0.02	0.000534	**0.001289**	0.09	979	0.03	0.02	0.192015	1	0.11
Acylcarnitine C18:1	1,956	0.06	0.02	0.000236	**0.000617**	0.16	979	0.08	0.02	0.000669	**0.048198**	0.25
Acylcarnitine C18:2	1,956	0.00	0.02	0.892851	0.914111	0.18	NA					
Acylcarnitine C2	1,983	0.07	0.02	5.9E-05	**0.000191**	0.10	991	0.06	0.03	0.013201	0.950489	0.13
Acylcarnitine C3	1,956	-0.05	0.02	0.005628	**0.011348**	0.06	979	-0.06	0.02	0.02248	1	0.12
Acylcarnitine C4	1,852	-0.03	0.02	0.119927	0.175608	0.04	NA					
Acylcarnitine C5	1,828	0.01	0.02	0.41167	0.506354	0.04	NA					
**Amino acids**												
Alanine	1,983	-0.02	0.02	0.16263	0.229925	0.11	NA					
Arginine	1,983	-0.05	0.01	0.000173	**0.000506**	0.19	989	-0.07	0.02	0.00155	0.11157	0.23
Asparagine	1,983	0.02	0.02	0.209993	0.28699	0.13	NA					
Citrulline	1,983	-0.10	0.02	8.2E-10	**5.93E-09**	0.11	989	-0.10	0.02	1.24E-05	**0.00089**	0.16
Glutamine	1,983	-0.01	0.02	0.452687	0.551292	0.05	NA					
Glutamate	1,983	0.04	0.01	0.001849	**0.004135**	0.26	989	0.04	0.02	0.059717	1	0.26
Glycine	1,983	-0.02	0.01	0.270663	0.346787	0.11	NA					
Histidine	1,983	0.02	0.01	0.256257	0.335315	0.08	NA					
Isoleucine	1,983	0.00	0.02	0.798684	0.854245	0.08	NA					
Leucine	1,982	0.00	0.02	0.97628	0.97628	0.08	NA					
Lysine	1,983	0.01	0.02	0.600089	0.689822	0.06	NA					
Methionine	1,983	0.02	0.02	0.218007	0.291467	0.08	NA					
Ornithine	1,983	-0.01	0.02	0.327788	0.415648	0.17	NA					
Phenylalanine	1,983	-0.01	0.01	0.675429	0.755252	0.10	NA					
Proline	1,983	-0.02	0.02	0.153941	0.220172	0.10	NA					
Serine	1,983	-0.03	0.01	0.049036	0.079361	0.10	NA					
Threonine	1,983	0.03	0.02	0.123534	0.178761	0.09	NA					
Tryptophan	1,983	0.01	0.02	0.722739	0.786698	0.06	NA					
Tyrosine	1,983	0.06	0.02	0.000135	**0.000416**	0.11	989	0.05	0.02	0.027538	1	0.12
Valine	1,983	-0.01	0.02	0.516758	0.617099	0.08	NA					
t4-hydroxyproline	1,774	0.03	0.02	0.058478	0.091048	0.12	NA					
**Biogenic amines**												
ADMA	1,930	-0.02	0.02	0.229624	0.303697	0.04	NA					
Creatine	1,983	0.01	0.01	0.527088	0.623383	0.04	NA					
Kynurenine	1,983	-0.04	0.02	0.017437	**0.03064**	0.10	989	-0.05	0.02	0.019566	1	0.13
Taurine	1,983	0.03	0.01	0.018218	**0.031561**	0.32	989	0.03	0.02	0.098293	1	0.31
**Glycerophospholipids**												
LysoPC a C16:0	1,983	0.09	0.01	1.8E-10	**1.48E-09**	0.16	991	0.08	0.02	6.38E-05	**0.004596**	0.13
LysoPC a C16:1	1,983	0.11	0.02	<1E-12	**<1E-12**	0.14	991	0.12	0.02	2.47E-08	**1.78E-06**	0.14
LysoPC a C17:0	1,983	-0.09	0.01	1E-10	**9.46E-10**	0.19	991	-0.11	0.02	2E-07	**1.44E-05**	0.17
LysoPC a C18:0	1,983	-0.01	0.01	0.266416	0.344939	0.14	NA					
LysoPC a C18:1	1,983	0.05	0.01	0.000221	**0.000605**	0.15	991	0.05	0.02	0.024597	1	0.15

LysoPC a C18:2	1,983	0.01	0.02	0.558303	0.647842	0.14	NA					
LysoPC a C20:3	1,983	0.04	0.02	0.007569	**0.014778**	0.08	991	0.06	0.02	0.003616	0.260342	0.10
LysoPC a C20:4	1,983	0.06	0.02	3.46E-05	**0.000122**	0.10	991	0.08	0.02	0.000119	**0.008543**	0.12
PC aa C28:1	1,983	0.03	0.01	0.014347	**0.025951**	0.17	991	0.01	0.02	0.460948	1	0.16
PC aa C30:0	1,983	0.10	0.01	<1E-12	**<1E-12**	0.18	991	0.12	0.02	4.29E-08	**3.09E-06**	0.22
PC aa C32:0	1,983	0.12	0.01	<1E-12	**<1E-12**	0.11	991	0.13	0.02	5.3E-10	**3.82E-08**	0.15
PC aa C32:1	1,983	0.20	0.02	<1E-12	**<1E-12**	0.25	991	0.22	0.02	<1E-12	**<1E-12**	0.28
PC aa C32:2	1,983	0.07	0.02	7.27E-06	**2.98E-05**	0.13	991	0.10	0.02	1.62E-05	**0.001163**	0.18
PC aa C32:3	1,983	-0.04	0.01	0.002701	**0.005827**	0.20	991	-0.08	0.02	4.83E-05	**0.003481**	0.22
PC aa C34:1	1,983	0.17	0.01	<1E-12	**<1E-12**	0.12	991	0.14	0.02	2E-11	**1.44E-09**	0.13
PC aa C34:2	1,983	0.03	0.01	3.46E-06	**1.47E-05**	0.04	991	0.03	0.01	0.008202	0.590576	0.04
PC aa C34:3	1,983	0.07	0.01	3.98E-07	**2.13E-06**	0.24	991	0.08	0.02	4.71E-05	**0.003392**	0.28
PC aa C34:4	1,983	0.11	0.02	1.5E-10	**1.32E-09**	0.12	991	0.13	0.02	1.87E-08	**1.34E-06**	0.17
PC aa C36:0	1,983	0.05	0.01	0.000227	**0.000607**	0.16	991	0.04	0.02	0.044044	1	0.18
PC aa C36:1	1,983	0.10	0.02	1.67E-09	**1.14E-08**	0.05	991	0.08	0.02	0.000812	0.058493	0.06
PC aa C36:2	1,983	0.00	0.01	0.875533	0.907071	0.03	NA					
PC aa C36:3	1,983	0.03	0.02	0.074809	0.115019	0.05	NA					
PC aa C36:4	1,983	0.14	0.02	<1E-12	**<1E-12**	0.11	991	0.15	0.02	<1E-12	**<1E-12**	0.17
PC aa C36:5	1,983	0.16	0.02	<1E-12	**<1E-12**	0.18	991	0.17	0.02	<1E-12	**<1E-12**	0.23
PC aa C36:6	1,983	0.11	0.02	2.1E-10	**1.61E-09**	0.15	991	0.10	0.02	6.15E-06	**0.000443**	0.18
PC aa C38:0	1,983	0.00	0.01	0.827574	0.877514	0.25	NA					
PC aa C38:3	1,983	0.00	0.02	0.755364	0.814998	0.13	NA					
PC aa C38:4	1,983	0.03	0.02	0.056358	0.088872	0.10	NA					
PC aa C38:5	1,983	0.09	0.02	1.92E-07	**1.12E-06**	0.10	991	0.11	0.02	6.34E-06	**0.000456**	0.12
PC aa C38:6	1,983	0.12	0.02	<1E-12	**<1E-12**	0.18	991	0.09	0.02	7.82E-05	**0.005632**	0.21
PC aa C40:2	1,922	-0.03	0.02	0.105692	0.158538	0.14	NA					
PC aa C40:3	1,922	0.00	0.02	0.899248	0.914111	0.12	NA					
PC aa C40:4	1,983	0.03	0.02	0.0503	0.080349	0.10	NA					
PC aa C40:5	1,983	0.03	0.02	0.039237	0.064349	0.15	NA					
PC aa C40:6	1,983	0.03	0.02	0.029924	0.050419	0.15	NA					
PC aa C42:0	1,983	-0.05	0.01	7.08E-05	**0.000223**	0.17	991	-0.03	0.02	0.058689	1	0.16
PC aa C42:1	1,983	-0.03	0.01	0.006508	**0.012911**	0.17	991	-0.02	0.02	0.301232	1	0.16
PC aa C42:2	1,983	0.05	0.01	0.000875	**0.002031**	0.24	991	0.05	0.02	0.01158	0.83377	0.26
PC aa C42:4	1,922	-0.01	0.01	0.629828	0.710723	0.04	NA					
PC aa C42:5	1,922	0.01	0.01	0.499936	0.602864	0.15	NA					
PC aa C42:6	1,922	0.00	0.01	0.868533	0.907071	0.08	NA					
PC ae C30:0	1,922	0.00	0.01	0.877573	0.907071	0.18	NA					
PC ae C30:2	1,922	-0.05	0.01	4.96E-05	**0.000165**	0.11	959	-0.07	0.02	4.36E-05	**0.003142**	0.12
PC ae C32:1	1,983	0.06	0.02	1.81E-05	**6.75E-05**	0.07	991	0.08	0.02	0.000208	**0.014996**	0.12
PC ae C32:2	1,983	0.06	0.02	0.000367	**0.000921**	0.07	991	0.05	0.02	0.035601	1	0.08
PC ae C34:0	1,983	0.04	0.02	0.013391	**0.024583**	0.09	991	0.02	0.02	0.464805	1	0.12
PC ae C34:1	1,983	0.01	0.02	0.612845	0.697962	0.08	NA					
PC ae C34:2	1,983	-0.04	0.02	0.004067	**0.008478**	0.08	991	-0.05	0.02	0.021188	1	0.10
PC ae C34:3	1,983	0.06	0.02	0.000527	**0.001289**	0.09	991	0.04	0.02	0.062088	1	0.08
PC ae C36:0	1,922	0.16	0.02	<1E-12	**<1E-12**	0.13	959	0.15	0.02	9E-11	**6.48E-09**	0.12
PC ae C36:1	1,983	0.00	0.02	0.940879	0.948592	0.06	NA					
PC ae C36:2	1,983	-0.11	0.02	<1E-12	**<1E-12**	0.12	991	-0.14	0.02	1.32E-09	**9.5E-08**	0.14
PC ae C36:3	1,983	-0.04	0.02	0.023764	**0.040596**	0.06	991	-0.05	0.02	0.033217	1	0.06
PC ae C36:4	1,983	0.04	0.01	0.010777	**0.020084**	0.15	991	0.05	0.02	0.032606	1	0.19

PC ae C36:5	1,983	0.10	0.02	1E-11	**1.02E-10**	0.15	991	0.13	0.02	5.31E-09	**3.82E-07**	0.18
PC ae C38:0	1,616	0.09	0.02	8.47E-07	**4.34E-06**	0.17	811	0.05	0.02	0.049169	1	0.22
PC ae C38:2	1,983	-0.07	0.01	1.21E-05	**4.65E-05**	0.06	991	-0.05	0.02	0.014344	1	0.09
PC ae C38:3	1,983	-0.09	0.02	5.86E-09	**3.79E-08**	0.08	991	-0.09	0.02	8.22E-05	**0.005921**	0.11
PC ae C38:4	1,983	-0.05	0.02	0.001614	**0.003677**	0.11	991	-0.05	0.02	0.061835	1	0.14
PC ae C38:5	1,983	0.02	0.02	0.213677	0.288816	0.12	NA					
PC ae C38:6	1,983	0.05	0.02	0.000744	**0.001759**	0.17	991	0.06	0.02	0.011821	0.851126	0.18
PC ae C40:1	1,983	0.07	0.02	4.05E-05	**0.000138**	0.09	991	0.05	0.02	0.043308	1	0.08
PC ae C40:2	1,983	0.01	0.01	0.721044	0.786698	0.12	NA					
PC ae C40:3	1,922	-0.05	0.01	0.000163	**0.000489**	0.10	959	-0.06	0.02	0.00777	0.559431	0.12
PC ae C40:4	1,983	-0.08	0.02	2.17E-06	**9.9E-06**	0.08	991	-0.05	0.03	0.039047	1	0.09
PC ae C40:5	1,983	-0.03	0.02	0.030533	0.050751	0.08	NA					
PC ae C40:6	1,983	-0.06	0.02	0.000284	**0.000727**	0.17	991	-0.08	0.02	0.000539	**0.038842**	0.19
PC ae C42:1	1,938	0.01	0.02	0.557053	0.647842	0.04	NA					
PC ae C42:2	1,983	0.02	0.02	0.118477	0.175574	0.10	NA					
PC ae C42:3	1,983	0.02	0.01	0.084541	0.128377	0.14	NA					
PC ae C42:4	1,983	-0.06	0.02	0.000204	**0.000584**	0.11	991	-0.04	0.02	0.094471	1	0.12
PC ae C42:5	1,983	-0.06	0.02	0.000217	**0.000605**	0.08	991	-0.03	0.02	0.181366	1	0.07
PC ae C44:4	1,983	-0.02	0.01	0.181462	0.250785	0.06	NA					
PC ae C44:5	1,983	-0.01	0.01	0.399652	0.496538	0.10	NA					
PC ae C44:6	1,983	-0.04	0.01	0.007742	**0.01488**	0.09	991	0.00	0.02	0.979539	1	0.07
**Sphingolipids**												
SM C16:0	1,983	0.01	0.01	0.36541	0.458627	0.05	NA					
SM C16:1	1,983	0.02	0.01	0.014882	**0.026528**	0.07	991	0.00	0.01	0.761088	1	0.07
SM C18:0	1,983	0.04	0.01	1.07E-05	**4.25E-05**	0.07	991	0.00	0.01	0.737895	1	0.09
SM C18:1	1,983	0.01	0.01	0.178527	0.249532	0.05	NA					
SM C20:2	1,983	-0.06	0.01	1.43E-06	**6.75E-06**	0.26	991	-0.08	0.02	4.84E-07	**3.48E-05**	0.30
SM C24:0	1,983	0.04	0.01	1.27E-06	**6.26E-06**	0.05	991	0.05	0.01	2.68E-05	**0.00193**	0.08
SM C24:1	1,983	0.03	0.01	2.8E-05	**0.000101**	0.08	991	0.04	0.01	0.000521	**0.037513**	0.14
SM(OH) C14:1	1,983	-0.06	0.01	3.89E-07	**2.13E-06**	0.14	991	-0.08	0.02	2.35E-06	**0.000169**	0.16
SM(OH) C16:1	1,983	-0.05	0.01	2.55E-06	**1.12E-05**	0.07	991	-0.08	0.02	2.12E-07	**1.53E-05**	0.11
SM(OH) C22:1	1,983	-0.03	0.01	0.004525	**0.009275**	0.05	991	-0.02	0.01	0.101529	1	0.07
SM(OH) C22:2	1,983	-0.05	0.01	1.34E-07	**8.25E-07**	0.09	991	-0.05	0.01	2.25E-05	**0.001621**	0.10
SM(OH) C24:1	1,983	0.00	0.01	0.694071	0.769106	0.05	NA					
**Hexoses**												
Hexoses	1,983	-0.04	0.02	0.008591	**0.016256**	0.16	991	-0.02	0.02	0.346628	1	0.24

Abbreviations: β, unstandardized regression coefficient derived from multivariable linear models; Bonf.-adj. p-value, Bonferroni-adjusted p-value ; NA, not applicable (metabolites that were not significant in the discovery analysis were not taken forward to the replication analysis); SE, standard error.

For an explanation of abbreviated metabolite names, see Supplementary Table 1.

[a] Analyzed with multivariable linear regression analyses analyzing associations of alcohol consumption (ln-transformed alcohol intake + 1) as main independent variable and as dependent variables the residuals obtained from linear mixed models with Z-standardized ln-transformed metabolite concentrations as dependent variables, sex as independent variable, and random intercepts for analytical batches nested within studies. Adjusted for: sex; age (y; continuous), body mass index (kg/m^2; continuous), self-reported physical activity levels (Cambridge physical activity index(1): inactive, moderately inactive, moderately active, active, unknown), fasting status (≥6 hours, 3-5.9 hours, <3 hours, unknown), meat intake (g/day; continuous), fish intake (g/day; continuous), energy intake (kcal/day; continuous), country, and smoking status (current, former, never, unknown).

[b] The discovery and replication set were taken as random samples without replacement of 66.7% and 33.3% of the total dataset, respectively.

[c] The analysis in the discovery set was adjusted for multiple testing using the false discovery rate (FDR) method. Statistically significant associations (FDR q-value<0.05) are depicted in bold.

[d] The analysis in the replication set was adjusted for multiple testing using Bonferroni correction. Statistically significant associations (Bonferroni-adjusted p-value<0.05) are depicted in bold.

Supplementary Table 5. Comparison of results of sex-stratified discovery analysis on associations of alcohol intake with concentrations of included metabolites (n=123) with results obtained in the total discovery set and results of statistical interaction analysis for sex[a]

	Total group (n=1,756)						Men (n=1,378)						Women (n=378)						Interaction analysis[b]
Metabolite	n	B	SE	p-value	FDR q-value[c]	R^2	N	β	SE	p-value	FDR q-value[c]	R^2	N	β	SE	p-value	FDR q-value[b]	R^2	FDR q-value[c]
**Acylcarnitines**																			
Acylcarnitine C0	1,983	0.05	0.02	0.003939	**0.008354**	0.06	1,497	0.06	0.02	0.000871	**0.002379**	0.06	486	0.00	0.04	0.967893	0.977559	0.14	0.196795
Acylcarnitine C14:1	1,956	0.05	0.02	0.002151	**0.004725**	0.09	1,482	0.06	0.02	0.001833	**0.004336**	0.09	474	0.01	0.04	0.738715	0.842254	0.15	0.474573
Acylcarnitine C16	1,956	0.11	0.02	<1E-12	**<1E-12**	0.15	1,482	0.12	0.02	<1E-12	**<1E-12**	0.13	474	0.07	0.03	0.036787	0.17403	0.25	0.392036
Acylcarnitine C18	1,956	0.06	0.02	0.000534	**0.001289**	0.09	1,482	0.05	0.02	0.002657	**0.005837**	0.07	474	0.05	0.04	0.255448	0.476062	0.16	0.702646
Acylcarnitine C18:1	1,956	0.06	0.02	0.000236	**0.000617**	0.16	1,482	0.06	0.02	0.001242	**0.003183**	0.15	474	0.02	0.03	0.508463	0.687092	0.27	0.942879
Acylcarnitine C18:2	1,956	0.00	0.02	0.892851	0.914111	0.18	1,482	0.00	0.02	0.891534	0.937254	0.15	474	-0.03	0.03	0.305858	0.537437	0.33	0.392036
Acylcarnitine C2	1,983	0.07	0.02	5.9E-05	**0.000191**	0.10	1,497	0.07	0.02	5.45E-05	**0.000186**	0.09	486	0.05	0.04	0.217508	0.438582	0.19	0.592031
Acylcarnitine C3	1,956	-0.05	0.02	0.005628	**0.011348**	0.06	1,482	-0.04	0.02	0.028891	0.051501	0.06	474	-0.07	0.04	0.076491	0.232922	0.15	0.459804
Acylcarnitine C4	1,852	-0.03	0.02	0.119927	0.175608	0.04	1,378	-0.02	0.02	0.211669	0.29071	0.04	474	-0.01	0.04	0.888349	0.933906	0.11	0.695685
Acylcarnitine C5	1,828	0.01	0.02	0.41167	0.506354	0.04	1,354	0.01	0.02	0.621033	0.713897	0.05	474	0.01	0.04	0.802835	0.881685	0.09	0.609073
**Amino acids**																			
Alanine	1,983	-0.02	0.02	0.16263	0.229925	0.11	1,497	-0.01	0.02	0.514048	0.626019	0.11	486	-0.08	0.04	0.032798	0.17403	0.15	0.453176
Arginine	1,983	-0.05	0.01	0.000173	**0.000506**	0.19	1,497	-0.05	0.02	0.002054	**0.004714**	0.13	486	-0.04	0.03	0.128381	0.322262	0.39	0.840132
Asparagine	1,983	0.02	0.02	0.209993	0.28699	0.13	1,497	0.02	0.02	0.295165	0.390379	0.12	486	0.00	0.03	0.969612	0.977559	0.21	0.850574
Citrulline	1,983	-0.10	0.02	8.2E-10	**5.93E-09**	0.11	1,497	-0.11	0.02	1.2E-09	**9.22E-09**	0.11	486	-0.07	0.04	0.047813	0.177204	0.14	0.702646
Glutamine	1,983	-0.01	0.02	0.452687	0.551292	0.05	1,497	-0.01	0.02	0.641384	0.730465	0.05	486	-0.02	0.04	0.690128	0.81621	0.11	0.859952
Glutamate	1,983	0.04	0.01	0.001849	**0.004135**	0.26	1,497	0.04	0.02	0.00597	**0.012239**	0.20	486	-0.01	0.03	0.777842	0.869768	0.47	0.394299
Glycine	1,983	-0.02	0.01	0.270663	0.346787	0.11	1,497	-0.01	0.01	0.467161	0.580412	0.12	486	-0.04	0.04	0.292812	0.521969	0.13	0.548849
Histidine	1,983	0.02	0.01	0.256257	0.335315	0.08	1,497	0.03	0.02	0.114683	0.167928	0.08	486	-0.04	0.03	0.253023	0.476062	0.16	0.338359
Isoleucine	1,983	0.00	0.02	0.798684	0.854245	0.08	1,497	0.00	0.02	0.860703	0.928653	0.09	486	-0.04	0.04	0.269377	0.494528	0.14	0.4572
Leucine	1,982	0.00	0.02	0.97628	0.97628	0.08	1,496	0.01	0.02	0.550486	0.639862	0.09	486	-0.05	0.04	0.195468	0.414526	0.11	0.392036
Lysine	1,983	0.01	0.02	0.600089	0.689822	0.06	1,497	0.02	0.02	0.336802	0.436071	0.07	486	-0.03	0.04	0.376601	0.6095	0.08	0.453176
Methionine	1,983	0.02	0.02	0.218007	0.291467	0.08	1,497	0.03	0.02	0.082273	0.126495	0.08	486	-0.02	0.03	0.496148	0.685687	0.12	0.378752
Ornithine	1,983	-0.01	0.02	0.327788	0.415648	0.17	1,497	-0.02	0.02	0.163855	0.231658	0.16	486	-0.02	0.03	0.630818	0.784524	0.27	0.803036
Phenylalanine	1,983	-0.01	0.01	0.675429	0.755252	0.10	1,497	0.00	0.02	0.912747	0.943427	0.10	486	-0.07	0.03	0.046638	0.177204	0.16	0.392036
Proline	1,983	-0.02	0.02	0.153941	0.220172	0.10	1,497	-0.02	0.02	0.311322	0.407368	0.10	486	-0.05	0.04	0.223843	0.439219	0.15	0.628543
Serine	1,983	-0.03	0.01	0.049036	0.079361	0.10	1,497	-0.02	0.02	0.167136	0.233611	0.10	486	-0.06	0.03	0.048983	0.177204	0.17	0.427211
Threonine	1,983	0.03	0.02	0.123534	0.178761	0.09	1,497	0.03	0.02	0.111257	0.164874	0.09	486	0.02	0.04	0.655986	0.794669	0.16	0.800906
Tryptophan	1,983	0.01	0.02	0.722739	0.786698	0.06	1,497	0.01	0.02	0.397324	0.498682	0.08	486	-0.06	0.04	0.107264	0.293188	0.08	0.378752
Tyrosine	1,983	0.06	0.02	0.000135	**0.000416**	0.11	1,497	0.07	0.02	0.000131	**0.000423**	0.12	486	0.01	0.04	0.720306	0.842254	0.14	0.392036
Valine	1,983	-0.01	0.02	0.516758	0.617099	0.08	1,497	0.01	0.02	0.696935	0.765384	0.09	486	-0.08	0.04	0.040912	0.177204	0.11	0.211397
t4-hydroxyproline	1,774	0.03	0.02	0.058478	0.091048	0.12	1,383	0.03	0.02	0.212714	0.29071	0.13	391	0.07	0.04	0.073523	0.232922	0.10	0.628543
**Biogenic amines**																			
ADMA	1,930	-0.02	0.02	0.229624	0.303697	0.04	1,467	-0.03	0.02	0.102514	0.15377	0.03	463	0.02	0.04	0.631446	0.784524	0.14	0.605156
Creatine	1,983	0.01	0.01	0.527088	0.623383	0.04	1,497	0.00	0.02	0.885936	0.937254	0.05	486	0.03	0.04	0.457913	0.662628	0.07	0.394299
Kynurenine	1,983	-0.04	0.02	0.017437	**0.03064**	0.10	1,497	-0.03	0.02	0.074583	0.117612	0.10	486	-0.06	0.04	0.095175	0.27064	0.14	0.628543
Taurine	1,983	0.03	0.01	0.018218	**0.031561**	0.32	1,497	0.03	0.01	0.041361	0.068749	0.27	486	0.00	0.03	0.865606	0.925822	0.48	0.550657
**Glycerophospholipids**																			
LysoPC a C16:0	1,983	0.09	0.01	1.8E-10	**1.48E-09**	0.16	1,497	0.10	0.02	1E-10	**8.79E-10**	0.14	486	0.02	0.03	0.480863	0.672116	0.30	0.098905
LysoPC a C16:1	1,983	0.11	0.02	<1E-12	**<1E-12**	0.14	1,497	0.13	0.02	<1E-12	**<1E-12**	0.11	486	0.02	0.03	0.625131	0.784524	0.33	**0.0462**
LysoPC a C17:0	1,983	-0.09	0.01	1E-10	**9.46E-10**	0.19	1,497	-0.09	0.02	2.7E-09	**1.95E-08**	0.19	486	-0.09	0.03	0.002835	**0.04981**	0.27	0.981878

LysoPC a C18:0	1,983	-0.01	0.01	0.266416	0.344939	0.14	1,497	0.00	0.01	0.889911	0.937254	0.14	486	-0.07	0.03	0.03423	0.17403	0.25	**0.0462**
LysoPC a C18:1	1,983	0.05	0.01	0.000221	**0.000605**	0.15	1,497	0.07	0.02	3.66E-05	**0.000136**	0.16	486	-0.02	0.03	0.506347	0.687092	0.21	0.145357
LysoPC a C18:2	1,983	0.01	0.02	0.558303	0.647842	0.14	1,497	0.02	0.02	0.363746	0.466049	0.15	486	-0.03	0.03	0.4548	0.662628	0.19	0.378752
LysoPC a C20:3	1,983	0.04	0.02	0.007569	**0.014778**	0.08	1,497	0.06	0.02	0.000431	**0.001325**	0.10	486	-0.05	0.03	0.143782	0.3401	0.16	**0.0462**
LysoPC a C20:4	1,983	0.06	0.02	3.46E-05	**0.000122**	0.10	1,497	0.09	0.02	1.44E-07	**8.06E-07**	0.14	486	-0.04	0.03	0.224966	0.439219	0.09	**0.018324**
PC aa C28:1	1,983	0.03	0.01	0.014347	**0.025951**	0.17	1,497	0.04	0.02	0.014447	**0.027338**	0.15	486	0.00	0.03	0.928075	0.959271	0.28	0.392036
PC aa C30:0	1,983	0.10	0.01	<1E-12	**<1E-12**	0.18	1,497	0.11	0.02	1E-10	**8.79E-10**	0.17	486	0.05	0.03	0.085363	0.249992	0.26	0.392036
PC aa C32:0	1,983	0.12	0.01	<1E-12	**<1E-12**	0.11	1,497	0.13	0.02	<1E-12	**<1E-12**	0.10	486	0.08	0.03	0.00424	0.057952	0.19	0.471988
PC aa C32:1	1,983	0.20	0.02	<1E-12	**<1E-12**	0.25	1,497	0.21	0.02	<1E-12	**<1E-12**	0.24	486	0.12	0.03	0.00016	**0.009828**	0.32	0.145357
PC aa C32:2	1,983	0.07	0.02	7.27E-06	**2.98E-05**	0.13	1,497	0.08	0.02	5.69E-06	**2.42E-05**	0.14	486	0.01	0.04	0.734098	0.842254	0.17	0.162393
PC aa C32:3	1,983	-0.04	0.01	0.002701	**0.005827**	0.20	1,497	-0.04	0.02	0.005086	**0.010604**	0.17	486	-0.05	0.03	0.077641	0.232922	0.35	0.694067
PC aa C34:1	1,983	0.17	0.01	<1E-12	**<1E-12**	0.12	1,497	0.19	0.02	<1E-12	**<1E-12**	0.12	486	0.08	0.03	0.00768	0.072662	0.18	**0.042409**
PC aa C34:2	1,983	0.03	0.01	3.46E-06	**1.47E-05**	0.04	1,497	0.04	0.01	1.21E-06	**6.48E-06**	0.05	486	0.01	0.01	0.553253	0.723937	0.07	0.127192
PC aa C34:3	1,983	0.07	0.01	3.98E-07	**2.13E-06**	0.24	1,497	0.08	0.02	2.24E-06	**1.15E-05**	0.22	486	0.03	0.03	0.4207	0.63105	0.35	0.207953
PC aa C34:4	1,983	0.11	0.02	1.5E-10	**1.32E-09**	0.12	1,497	0.13	0.02	<1E-12	**<1E-12**	0.14	486	0.01	0.04	0.73954	0.842254	0.16	**0.04154**
PC aa C36:0	1,983	0.05	0.01	0.000227	**0.000607**	0.16	1,497	0.05	0.01	0.001759	**0.004242**	0.15	486	0.06	0.03	0.036293	0.17403	0.27	0.911148
PC aa C36:1	1,983	0.10	0.02	1.67E-09	**1.14E-08**	0.05	1,497	0.12	0.02	4E-10	**3.28E-09**	0.06	486	0.02	0.04	0.577745	0.740236	0.10	0.098905
PC aa C36:2	1,983	0.00	0.01	0.875533	0.907071	0.03	1,497	0.01	0.01	0.513982	0.626019	0.03	486	-0.02	0.03	0.426885	0.632613	0.08	0.251914
PC aa C36:3	1,983	0.03	0.02	0.074809	0.115019	0.05	1,497	0.04	0.02	0.026737	**0.048362**	0.05	486	-0.03	0.03	0.392161	0.618409	0.12	0.187222
PC aa C36:4	1,983	0.14	0.02	<1E-12	**<1E-12**	0.11	1,497	0.16	0.02	<1E-12	**<1E-12**	0.14	486	0.05	0.03	0.137856	0.332477	0.13	**0.018324**
PC aa C36:5	1,983	0.16	0.02	<1E-12	**<1E-12**	0.18	1,497	0.17	0.02	<1E-12	**<1E-12**	0.17	486	0.10	0.03	0.002469	**0.04981**	0.27	0.474573
PC aa C36:6	1,983	0.11	0.02	2.1E-10	**1.61E-09**	0.15	1,497	0.11	0.02	5.4E-09	**3.69E-08**	0.14	486	0.06	0.03	0.096814	0.27064	0.23	0.392036
PC aa C38:0	1,983	0.00	0.01	0.827574	0.877514	0.25	1,497	0.00	0.02	0.909485	0.943427	0.24	486	-0.01	0.03	0.788294	0.873515	0.39	0.803036
PC aa C38:3	1,983	0.00	0.02	0.755364	0.814998	0.13	1,497	0.01	0.02	0.688023	0.765384	0.12	486	-0.06	0.04	0.068242	0.232922	0.25	0.145357
PC aa C38:4	1,983	0.03	0.02	0.056358	0.088872	0.10	1,497	0.06	0.02	0.002069	**0.004714**	0.11	486	-0.06	0.04	0.076921	0.232922	0.16	**0.018324**
PC aa C38:5	1,983	0.09	0.02	1.92E-07	**1.12E-06**	0.10	1,497	0.11	0.02	1.6E-08	**1.04E-07**	0.11	486	-0.01	0.04	0.81269	0.88461	0.16	0.063778
PC aa C38:6	1,983	0.12	0.02	<1E-12	**<1E-12**	0.18	1,497	0.13	0.02	<1E-12	**<1E-12**	0.17	486	0.09	0.03	0.006257	0.068576	0.28	0.4572
PC aa C40:2	1,922	-0.03	0.02	0.105692	0.158538	0.14	1,436	-0.03	0.02	0.087664	0.13312	0.13	486	-0.03	0.03	0.404987	0.622667	0.24	0.989802
PC aa C40:3	1,922	0.00	0.02	0.899248	0.914111	0.12	1,436	0.00	0.02	0.980177	0.983765	0.10	486	-0.05	0.03	0.119314	0.305742	0.25	0.507345
PC aa C40:4	1,983	0.03	0.02	0.0503	0.080349	0.10	1,497	0.06	0.02	0.001171	**0.003065**	0.11	486	-0.07	0.04	0.045489	0.177204	0.21	**0.018324**
PC aa C40:5	1,983	0.03	0.02	0.039237	0.064349	0.15	1,497	0.05	0.02	0.002341	**0.005235**	0.16	486	-0.06	0.03	0.07217	0.232922	0.24	**0.018324**
PC aa C40:6	1,983	0.03	0.02	0.029924	0.050419	0.15	1,497	0.04	0.02	0.019052	**0.035505**	0.14	486	0.00	0.03	0.964774	0.977559	0.24	0.302384
PC aa C42:0	1,983	-0.05	0.01	7.08E-05	**0.000223**	0.17	1,497	-0.04	0.01	0.000971	**0.002597**	0.16	486	-0.06	0.02	0.013227	0.101681	0.28	0.719524
PC aa C42:1	1,983	-0.03	0.01	0.006508	**0.012911**	0.17	1,497	-0.03	0.01	0.064633	0.104604	0.16	486	-0.06	0.02	0.009759	0.084396	0.30	0.251914
PC aa C42:2	1,983	0.05	0.01	0.000875	**0.002031**	0.24	1,497	0.05	0.02	0.003369	**0.007269**	0.22	486	0.04	0.03	0.18723	0.414526	0.36	0.628543
PC aa C42:4	1,922	-0.01	0.01	0.629828	0.710723	0.04	1,436	0.01	0.02	0.526042	0.634345	0.04	486	-0.06	0.03	0.044275	0.177204	0.08	0.134127
PC aa C42:5	1,922	0.01	0.01	0.499936	0.602864	0.15	1,436	0.01	0.02	0.542748	0.639862	0.13	486	-0.03	0.03	0.389592	0.618409	0.27	0.679339
PC aa C42:6	1,922	0.00	0.01	0.868533	0.907071	0.08	1,436	0.01	0.02	0.683511	0.765384	0.08	486	-0.04	0.03	0.194471	0.414526	0.16	0.362465
PC ae C30:0	1,922	0.00	0.01	0.877573	0.907071	0.18	1,436	0.00	0.02	0.928044	0.951246	0.17	486	0.01	0.03	0.853675	0.92107	0.27	0.82908
PC ae C30:2	1,922	-0.05	0.01	4.96E-05	**0.000165**	0.11	1,436	-0.06	0.01	2.72E-05	**0.000105**	0.10	486	-0.02	0.02	0.412759	0.626781	0.19	0.702646
PC ae C32:1	1,983	0.06	0.02	1.81E-05	**6.75E-05**	0.07	1,497	0.06	0.02	0.000294	**0.000928**	0.06	486	0.05	0.03	0.112697	0.301341	0.14	0.85351
PC ae C32:2	1,983	0.06	0.02	0.000367	**0.000921**	0.07	1,497	0.05	0.02	0.007897	**0.015924**	0.06	486	0.06	0.03	0.046568	0.177204	0.17	0.989802
PC ae C34:0	1,983	0.04	0.02	0.013391	**0.024583**	0.09	1,497	0.03	0.02	0.068193	0.108931	0.08	486	0.05	0.03	0.133745	0.329012	0.16	0.803036
PC ae C34:1	1,983	0.01	0.02	0.612845	0.697962	0.08	1,497	0.01	0.02	0.543822	0.639862	0.07	486	-0.02	0.03	0.525433	0.694928	0.18	0.923927
PC ae C34:2	1,983	-0.04	0.02	0.004067	**0.008478**	0.08	1,497	-0.05	0.02	0.004524	**0.009594**	0.09	486	-0.02	0.03	0.513922	0.687092	0.10	0.984001
PC ae C34:3	1,983	0.06	0.02	0.000527	**0.001289**	0.09	1,497	0.06	0.02	0.000738	**0.002112**	0.10	486	0.03	0.03	0.35019	0.590494	0.15	0.308286
PC ae C36:0	1,922	0.16	0.02	<1E-12	**<1E-12**	0.13	1,436	0.16	0.02	<1E-12	**<1E-12**	0.11	486	0.16	0.03	3.52E-07	**4.33E-05**	0.22	0.989802
PC ae C36:1	1,983	0.00	0.02	0.940879	0.948592	0.06	1,497	0.00	0.02	0.983765	0.983765	0.05	486	-0.02	0.03	0.576932	0.740236	0.16	0.861659
PC ae C36:2	1,983	-0.11	0.02	<1E-12	**<1E-12**	0.12	1,497	-0.13	0.02	<1E-12	**<1E-12**	0.13	486	-0.07	0.03	0.051579	0.181262	0.15	0.394299

PC ae C36:3	1,983	-0.04	0.02	0.023764	**0.040596**	0.06	1,497	-0.04	0.02	0.033095	0.058153	0.07	486	-0.03	0.03	0.355256	0.590494	0.09	0.803036
PC ae C36:4	1,983	0.04	0.01	0.010777	**0.020084**	0.15	1,497	0.05	0.02	0.001594	**0.003921**	0.17	486	0.00	0.03	0.981032	0.981032	0.16	0.207953
PC ae C36:5	1,983	0.10	0.02	1E-11	**1.02E-10**	0.15	1,497	0.12	0.02	<1E-12	**<1E-12**	0.17	486	0.04	0.03	0.198911	0.414678	0.13	**0.0462**
PC ae C38:0	1,616	0.09	0.02	8.47E-07	**4.34E-06**	0.17	1,130	0.09	0.02	1.03E-05	**4.21E-05**	0.15	486	0.03	0.03	0.287731	0.520454	0.31	0.308286
PC ae C38:2	1,983	-0.07	0.01	1.21E-05	**4.65E-05**	0.06	1,497	-0.08	0.02	1.41E-05	**5.59E-05**	0.06	486	-0.04	0.03	0.171414	0.393153	0.11	0.705171
PC ae C38:3	1,983	-0.09	0.02	5.86E-09	**3.79E-08**	0.08	1,497	-0.09	0.02	6.83E-08	**4E-07**	0.07	486	-0.09	0.03	0.005427	0.066758	0.16	0.944055
PC ae C38:4	1,983	-0.05	0.02	0.001614	**0.003677**	0.11	1,497	-0.04	0.02	0.025237	**0.04633**	0.11	486	-0.08	0.03	0.016027	0.110172	0.16	0.4572
PC ae C38:5	1,983	0.02	0.02	0.213677	0.288816	0.12	1,497	0.04	0.02	0.038506	0.064879	0.12	486	-0.03	0.03	0.335917	0.581941	0.14	0.308286
PC ae C38:6	1,983	0.05	0.02	0.000744	**0.001759**	0.17	1,497	0.06	0.02	0.000717	**0.0021**	0.16	486	0.02	0.03	0.479871	0.672116	0.24	0.378752
PC ae C40:1	1,983	0.07	0.02	4.05E-05	**0.000138**	0.09	1,497	0.08	0.02	4.6E-05	**0.000166**	0.09	486	0.03	0.03	0.35212	0.590494	0.16	0.230262
PC ae C40:2	1,983	0.01	0.01	0.721044	0.786698	0.12	1,497	0.00	0.02	0.942795	0.958378	0.09	486	0.01	0.03	0.747041	0.842991	0.25	0.705171
PC ae C40:3	1,922	-0.05	0.01	0.000163	**0.000489**	0.10	1,436	-0.05	0.02	0.001368	**0.003435**	0.11	486	-0.07	0.03	0.010292	0.084396	0.16	0.803036
PC ae C40:4	1,983	-0.08	0.02	2.17E-06	**9.9E-06**	0.08	1,497	-0.06	0.02	0.000619	**0.001856**	0.08	486	-0.13	0.04	0.000443	**0.01816**	0.13	0.378752
PC ae C40:5	1,983	-0.03	0.02	0.030533	0.050751	0.08	1,497	-0.03	0.02	0.141617	0.202546	0.08	486	-0.07	0.03	0.046623	0.177204	0.16	0.709057
PC ae C40:6	1,983	-0.06	0.02	0.000284	**0.000727**	0.17	1,497	-0.06	0.02	0.000845	**0.002361**	0.15	486	-0.05	0.03	0.116999	0.305742	0.29	0.989802
PC ae C42:1	1,938	0.01	0.02	0.557053	0.647842	0.04	1,452	0.02	0.02	0.287403	0.384245	0.05	486	-0.03	0.04	0.400005	0.622667	0.11	0.211397
PC ae C42:2	1,983	0.02	0.02	0.118477	0.175574	0.10	1,497	0.03	0.02	0.082209	0.126495	0.10	486	-0.01	0.03	0.658403	0.794669	0.16	0.394299
PC ae C42:3	1,983	0.02	0.01	0.084541	0.128377	0.14	1,497	0.03	0.02	0.03721	0.063568	0.13	486	-0.02	0.03	0.473695	0.672116	0.24	0.378752
PC ae C42:4	1,983	-0.06	0.02	0.000204	**0.000584**	0.11	1,497	-0.05	0.02	0.009529	**0.018605**	0.12	486	-0.10	0.03	0.00142	**0.04366**	0.14	0.444066
PC ae C42:5	1,983	-0.06	0.02	0.000217	**0.000605**	0.08	1,497	-0.05	0.02	0.008104	**0.016078**	0.08	486	-0.11	0.03	0.001956	**0.048114**	0.17	0.581284
PC ae C44:4	1,983	-0.02	0.01	0.181462	0.250785	0.06	1,497	-0.01	0.01	0.691974	0.765384	0.06	486	-0.06	0.03	0.020246	0.118586	0.13	0.427211
PC ae C44:5	1,983	-0.01	0.01	0.399652	0.496538	0.10	1,497	0.00	0.02	0.820449	0.893055	0.09	486	-0.07	0.03	0.016733	0.110172	0.19	0.392036
PC ae C44:6	1,983	-0.04	0.01	0.007742	**0.01488**	0.09	1,497	-0.02	0.02	0.136503	0.197527	0.10	486	-0.09	0.03	0.003635	0.055888	0.12	0.308286
**Sphingolipids**																			
SM C16:0	1,983	0.01	0.01	0.36541	0.458627	0.05	1,497	0.01	0.01	0.256339	0.34648	0.06	486	-0.01	0.02	0.665454	0.794669	0.10	0.628543
SM C16:1	1,983	0.02	0.01	0.014882	**0.026528**	0.07	1,497	0.02	0.01	0.036575	0.063363	0.05	486	0.03	0.02	0.207867	0.426127	0.16	0.928366
SM C18:0	1,983	0.04	0.01	1.07E-05	**4.25E-05**	0.07	1,497	0.04	0.01	6.43E-05	**0.000214**	0.07	486	0.03	0.02	0.190824	0.414526	0.16	0.803036
SM C18:1	1,983	0.01	0.01	0.178527	0.249532	0.05	1,497	0.01	0.01	0.374309	0.47464	0.04	486	0.03	0.02	0.232678	0.447179	0.13	0.742517
SM C20:2	1,983	-0.06	0.01	1.43E-06	**6.75E-06**	0.26	1,497	-0.07	0.01	3.97E-06	**1.81E-05**	0.22	486	-0.06	0.02	0.00669	0.068576	0.47	0.989802
SM C24:0	1,983	0.04	0.01	1.27E-06	**6.26E-06**	0.05	1,497	0.05	0.01	5.06E-08	**3.11E-07**	0.08	486	0.00	0.02	0.87462	0.927399	0.10	0.055943
SM C24:1	1,983	0.03	0.01	2.8E-05	**0.000101**	0.08	1,497	0.04	0.01	4.71E-05	**0.000166**	0.08	486	0.02	0.02	0.369723	0.606346	0.18	0.679339
SM(OH) C14:1	1,983	-0.06	0.01	3.89E-07	**2.13E-06**	0.14	1,497	-0.06	0.01	4.72E-06	**2.07E-05**	0.13	486	-0.06	0.02	0.021886	0.122363	0.21	0.972987
SM(OH) C16:1	1,983	-0.05	0.01	2.55E-06	**1.12E-05**	0.07	1,497	-0.06	0.01	3.94E-06	**1.81E-05**	0.06	486	-0.03	0.02	0.172604	0.393153	0.14	0.476525
SM(OH) C22:1	1,983	-0.03	0.01	0.004525	**0.009275**	0.05	1,497	-0.02	0.01	0.052577	0.086227	0.05	486	-0.05	0.02	0.017018	0.110172	0.12	0.54816
SM(OH) C22:2	1,983	-0.05	0.01	1.34E-07	**8.25E-07**	0.09	1,497	-0.05	0.01	2.84E-06	**1.39E-05**	0.09	486	-0.04	0.02	0.019293	0.118586	0.14	0.969953
SM(OH) C24:1	1,983	0.00	0.01	0.694071	0.769106	0.05	1,497	0.01	0.01	0.551426	0.639862	0.06	486	-0.01	0.02	0.65952	0.794669	0.12	0.803036
**Hexoses**																			
Hexoses	1,983	-0.04	0.02	0.008591	**0.016256**	0.16	1,497	-0.05	0.02	0.010598	**0.020368**	0.14	486	0.00	0.03	0.916543	0.95538	0.27	0.427211

Abbreviations: β, unstandardized regression coefficient derived from multivariable linear models; SE, standard error.

For an explanation of abbreviated metabolite names, see Supplementary Table 1.

[a] Analyzed with multivariable linear regression analyses analyzing associations of alcohol consumption (ln-transformed alcohol intake + 1) as main independent variable and as dependent variables the residuals obtained from linear mixed models with Z-standardized ln-transformed metabolite concentrations as dependent variables, sex as independent variable, and random intercepts for analytical batches nested within studies. Adjusted for: sex (not in sex-stratified analysis); age (y; continuous), body mass index (kg/m^2; continuous), self-reported physical activity levels (Cambridge physical activity index(1): inactive, moderately inactive, moderately active, active, unknown), fasting status (≥6 hours, 3-5.9 hours, <3 hours, unknown), meat intake (g/day; continuous), fish intake (g/day; continuous), energy intake (kcal/day; continuous), country, and smoking status (current, former, never, unknown).

[b] Testing for statistical interaction was performed by including a product term for alcohol intake and sex and evaluating significance.

[c] The analysis in the discovery set was adjusted for multiple testing using the false discovery rate (FDR) method. Statistically significant associations (FDR q-value<0.05) are depicted in bold.

Supplementary Table 6. Comparison of results of discovery analysis on associations of alcohol intake with concentrations of included metabolites (n=123) in never smokers only with results obtained in the total discovery set and results of statistical interaction analysis for smoking status[a]

	Total group (n=1,756)						Never smokers (n=639)						Interaction analysis[b]	Interaction former vs never smokers[b]		Interaction current vs never smokers[b]	
Metabolite	n	B	SE	p-value	FDR q-value[c]	R^2	n	β	SE	p-value	FDR q-value[c]	R^2	FDR q-value[c]	B	SE	B	SE
**Acylcarnitines**																	
Acylcarnitine C0	1,983	0.05	0.02	0.003939	**0.008354**	0.06	754	0.03	0.03	0.210385	0.341715	0.08	0.572879	0.03	0.03	0.07	0.04
Acylcarnitine C14:1	1,956	0.05	0.02	0.002151	**0.004725**	0.09	740	0.03	0.03	0.357029	0.510634	0.12	0.686143	0.05	0.03	0.02	0.04
Acylcarnitine C16	1,956	0.11	0.02	<1E-12	**<1E-12**	0.15	740	0.09	0.03	0.000481	0.003946	0.16	0.608217	0.05	0.03	0.02	0.04
Acylcarnitine C18	1,956	0.06	0.02	0.000534	**0.001289**	0.09	740	0.08	0.03	0.003789	0.018853	0.12	0.983917	-0.01	0.03	0.01	0.04
Acylcarnitine C18:1	1,956	0.06	0.02	0.000236	**0.000617**	0.16	740	0.08	0.03	0.001542	0.00998	0.19	0.801755	-0.03	0.03	-0.04	0.04
Acylcarnitine C18:2	1,956	0.00	0.02	0.892851	0.914111	0.18	740	0.01	0.03	0.791577	0.85407	0.23	0.973462	0.02	0.03	0.02	0.04
Acylcarnitine C2	1,983	0.07	0.02	5.9E-05	**0.000191**	0.10	754	0.02	0.03	0.532795	0.675607	0.14	0.042838	0.05	0.04	0.13	0.04
Acylcarnitine C3	1,956	-0.05	0.02	0.005628	**0.011348**	0.06	740	-0.01	0.03	0.652344	0.756965	0.07	0.59968	-0.06	0.04	0.00	0.04
Acylcarnitine C4	1,852	-0.03	0.02	0.119927	0.175608	0.04	715	-0.04	0.03	0.182116	0.315497	0.05	0.59968	0.06	0.04	0.01	0.04
Acylcarnitine C5	1,828	0.01	0.02	0.41167	0.506354	0.04	706	0.01	0.03	0.772378	0.840731	0.07	0.790111	0.01	0.04	0.05	0.04
**Amino acids**																	
Alanine	1,983	-0.02	0.02	0.16263	0.229925	0.11	754	-0.04	0.03	0.166137	0.311701	0.13	0.76721	0.02	0.03	0.05	0.04
Arginine	1,983	-0.05	0.01	0.000173	**0.000506**	0.19	754	-0.08	0.02	0.000863	0.006246	0.25	0.345544	0.06	0.03	0.01	0.03
Asparagine	1,983	0.02	0.02	0.209993	0.28699	0.13	754	0.02	0.02	0.424187	0.579722	0.17	0.998364	0.00	0.03	0.00	0.04
Citrulline	1,983	-0.10	0.02	8.2E-10	**5.93E-09**	0.11	754	-0.07	0.03	0.010058	0.042661	0.12	0.801755	-0.01	0.03	-0.04	0.04
Glutamine	1,983	-0.01	0.02	0.452687	0.551292	0.05	754	-0.07	0.03	0.019617	0.06137	0.09	0.551109	0.07	0.04	0.04	0.04
Glutamate	1,983	0.04	0.01	0.001849	**0.004135**	0.26	754	0.07	0.02	0.003832	0.018853	0.34	0.59968	-0.03	0.03	0.03	0.03
Glycine	1,983	-0.02	0.01	0.270663	0.346787	0.11	754	-0.02	0.03	0.376624	0.532469	0.09	0.94797	0.02	0.03	0.00	0.03
Histidine	1,983	0.02	0.01	0.256257	0.335315	0.08	754	-0.01	0.02	0.815245	0.871958	0.12	0.676824	0.05	0.03	0.02	0.04
Isoleucine	1,983	0.00	0.02	0.798684	0.854245	0.08	754	-0.02	0.03	0.444527	0.600844	0.09	0.620042	-0.01	0.03	0.05	0.04
Leucine	1,982	0.00	0.02	0.97628	0.97628	0.08	754	-0.01	0.03	0.603252	0.72745	0.08	0.962043	0.01	0.03	0.03	0.04
Lysine	1,983	0.01	0.02	0.600089	0.689822	0.06	754	-0.01	0.03	0.6298	0.749217	0.08	0.790111	0.05	0.04	0.02	0.04
Methionine	1,983	0.02	0.02	0.218007	0.291467	0.08	754	-0.03	0.03	0.211141	0.341715	0.10	0.345544	0.08	0.03	0.07	0.04
Ornithine	1,983	-0.01	0.02	0.327788	0.415648	0.17	754	0.03	0.02	0.288723	0.434653	0.21	0.59968	-0.06	0.03	-0.03	0.04
Phenylalanine	1,983	-0.01	0.01	0.675429	0.755252	0.10	754	-0.03	0.02	0.169788	0.311701	0.14	0.520145	0.06	0.03	0.00	0.03
Proline	1,983	-0.02	0.02	0.153941	0.220172	0.10	754	-0.04	0.03	0.206979	0.341715	0.13	0.804924	0.01	0.04	0.04	0.04
Serine	1,983	-0.03	0.01	0.049036	0.079361	0.10	754	-0.05	0.02	0.020628	0.06137	0.14	0.551109	0.05	0.03	0.01	0.03
Threonine	1,983	0.03	0.02	0.123534	0.178761	0.09	754	-0.01	0.03	0.597345	0.72745	0.14	0.620849	0.04	0.04	0.06	0.04
Tryptophan	1,983	0.01	0.02	0.722739	0.786698	0.06	754	-0.04	0.03	0.166534	0.311701	0.05	0.572879	0.04	0.03	0.06	0.04
Tyrosine	1,983	0.06	0.02	0.000135	**0.000416**	0.11	754	0.03	0.03	0.343318	0.502716	0.11	0.509718	0.04	0.03	0.08	0.04
Valine	1,983	-0.01	0.02	0.516758	0.617099	0.08	754	-0.03	0.03	0.190793	0.321474	0.09	0.892646	0.02	0.03	0.03	0.04
t4-hydroxyproline	1,774	0.03	0.02	0.058478	0.091048	0.12	657	0.02	0.03	0.551174	0.684792	0.14	0.616518	0.03	0.04	-0.04	0.04
**Biogenic amines**																	
ADMA	1,930	-0.02	0.02	0.229624	0.303697	0.04	735	0.01	0.03	0.749998	0.823659	0.11	0.520145	0.02	0.04	-0.06	0.04
Creatine	1,983	0.01	0.01	0.527088	0.623383	0.04	754	0.00	0.02	0.952747	0.952747	0.07	0.801755	-0.01	0.03	0.03	0.04
Kynurenine	1,983	-0.04	0.02	0.017437	**0.03064**	0.10	754	-0.02	0.02	0.313557	0.464668	0.11	0.801755	-0.01	0.03	-0.04	0.04
Taurine	1,983	0.03	0.01	0.018218	**0.031561**	0.32	754	0.04	0.02	0.039358	0.100855	0.38	0.517739	-0.02	0.03	0.04	0.03
**Glycerophospolipids**																	
LysoPC a C16:0	1,983	0.09	0.01	1.8E-10	**1.48E-09**	0.16	754	0.08	0.02	0.000242	0.002711	0.19	0.801755	0.00	0.03	0.03	0.03
LysoPC a C16:1	1,983	0.11	0.02	<1E-12	**<1E-12**	0.14	754	0.06	0.02	0.013849	0.053232	0.19	0.0108	0.06	0.03	0.14	0.04
LysoPC a C17:0	1,983	-0.09	0.01	1E-10	**9.46E-10**	0.19	754	-0.03	0.02	0.17986	0.315497	0.18	0.0108	-0.06	0.03	-0.13	0.03

LysoPC a C18:0	1,983	-0.01	0.01	0.266416	0.344939	0.14	754	0.00	0.02	0.898826	0.913682	0.16	0.899268	0.01	0.03	-0.02	0.03
LysoPC a C18:1	1,983	0.05	0.01	0.000221	**0.000605**	0.15	754	0.04	0.02	0.10454	0.229614	0.15	0.899268	0.02	0.03	0.03	0.03
LysoPC a C18:2	1,983	0.01	0.02	0.558303	0.647842	0.14	754	-0.01	0.02	0.636598	0.749217	0.20	0.59968	0.05	0.03	0.05	0.04
LysoPC a C20:3	1,983	0.04	0.02	0.007569	**0.014778**	0.08	754	0.03	0.03	0.282719	0.434653	0.09	0.811707	0.02	0.03	0.04	0.04
LysoPC a C20:4	1,983	0.06	0.02	3.46E-05	**0.000122**	0.10	754	0.06	0.02	0.011069	0.045382	0.13	0.984326	0.01	0.03	0.01	0.04
PC aa C28:1	1,983	0.03	0.01	0.014347	**0.025951**	0.17	754	0.06	0.02	0.005485	0.02595	0.28	0.572879	-0.03	0.03	-0.06	0.03
PC aa C30:0	1,983	0.10	0.01	<1E-12	**<1E-12**	0.18	754	0.07	0.02	0.001919	0.011805	0.26	0.899268	0.01	0.03	0.03	0.04
PC aa C32:0	1,983	0.12	0.01	<1E-12	**<1E-12**	0.11	754	0.10	0.02	4.14E-05	0.000566	0.19	0.801755	0.00	0.03	0.04	0.04
PC aa C32:1	1,983	0.20	0.02	<1E-12	**<1E-12**	0.25	754	0.13	0.02	1.51E-07	6.18E-06	0.30	0.021712	0.06	0.03	0.13	0.04
PC aa C32:2	1,983	0.07	0.02	7.27E-06	**2.98E-05**	0.13	754	0.03	0.03	0.258261	0.402103	0.16	0.203914	0.05	0.03	0.10	0.04
PC aa C32:3	1,983	-0.04	0.01	0.002701	**0.005827**	0.20	754	-0.03	0.02	0.136019	0.269844	0.24	0.976658	-0.02	0.03	-0.01	0.03
PC aa C34:1	1,983	0.17	0.01	<1E-12	**<1E-12**	0.12	754	0.14	0.02	1.84E-08	1.13E-06	0.13	0.509718	0.00	0.03	0.07	0.04
PC aa C34:2	1,983	0.03	0.01	3.46E-06	**1.47E-05**	0.04	754	0.01	0.01	0.257175	0.402103	0.05	0.203914	0.03	0.02	0.04	0.02
PC aa C34:3	1,983	0.07	0.01	3.98E-07	**2.13E-06**	0.24	754	0.03	0.02	0.180403	0.315497	0.29	0.066999	0.05	0.03	0.11	0.03
PC aa C34:4	1,983	0.11	0.02	1.5E-10	**1.32E-09**	0.12	754	0.08	0.03	0.003013	0.016844	0.15	0.509718	0.01	0.04	0.07	0.04
PC aa C36:0	1,983	0.05	0.01	0.000227	**0.000607**	0.16	754	0.05	0.02	0.022733	0.063549	0.21	0.983917	0.01	0.03	0.01	0.03
PC aa C36:1	1,983	0.10	0.02	1.67E-09	**1.14E-08**	0.05	754	0.09	0.03	0.000989	0.00676	0.05	0.977406	-0.01	0.04	0.01	0.04
PC aa C36:2	1,983	0.00	0.01	0.875533	0.907071	0.03	754	-0.02	0.02	0.417816	0.577431	0.04	0.501162	0.06	0.03	0.03	0.03
PC aa C36:3	1,983	0.03	0.02	0.074809	0.115019	0.05	754	0.00	0.03	0.869043	0.905917	0.08	0.59968	0.04	0.03	0.06	0.04
PC aa C36:4	1,983	0.14	0.02	<1E-12	**<1E-12**	0.11	754	0.12	0.02	3.75E-07	1.15E-05	0.15	0.620812	0.01	0.03	0.05	0.04
PC aa C36:5	1,983	0.16	0.02	<1E-12	**<1E-12**	0.18	754	0.17	0.03	2.2E-09	2.71E-07	0.20	0.686143	-0.05	0.04	-0.01	0.04
PC aa C36:6	1,983	0.11	0.02	2.1E-10	**1.61E-09**	0.15	754	0.12	0.03	1.53E-05	0.000235	0.17	0.790111	-0.04	0.04	0.01	0.04
PC aa C38:0	1,983	0.00	0.01	0.827574	0.877514	0.25	754	0.02	0.02	0.477939	0.632113	0.30	0.801755	-0.02	0.03	-0.04	0.03
PC aa C38:3	1,983	0.00	0.02	0.755364	0.814998	0.13	754	-0.01	0.03	0.74278	0.823081	0.14	0.899268	0.01	0.03	0.03	0.04
PC aa C38:4	1,983	0.03	0.02	0.056358	0.088872	0.10	754	0.03	0.03	0.289768	0.434653	0.12	0.983917	0.01	0.04	0.01	0.04
PC aa C38:5	1,983	0.09	0.02	1.92E-07	**1.12E-06**	0.10	754	0.10	0.03	0.000378	0.003572	0.12	0.801755	-0.04	0.04	0.00	0.04
PC aa C38:6	1,983	0.12	0.02	<1E-12	**<1E-12**	0.18	754	0.14	0.03	4.95E-07	1.22E-05	0.21	0.801755	-0.03	0.03	0.02	0.04
PC aa C40:2	1,922	-0.03	0.02	0.105692	0.158538	0.14	734	0.00	0.03	0.883822	0.905917	0.19	0.891805	-0.03	0.03	-0.03	0.04
PC aa C40:3	1,922	0.00	0.02	0.899248	0.914111	0.12	734	0.00	0.03	0.878653	0.905917	0.17	0.927214	-0.01	0.03	0.02	0.04
PC aa C40:4	1,983	0.03	0.02	0.0503	0.080349	0.10	754	0.00	0.03	0.874242	0.905917	0.16	0.509718	0.03	0.04	0.08	0.04
PC aa C40:5	1,983	0.03	0.02	0.039237	0.064349	0.15	754	0.02	0.03	0.506941	0.656355	0.20	0.501162	0.00	0.03	0.07	0.04
PC aa C40:6	1,983	0.03	0.02	0.029924	0.050419	0.15	754	0.04	0.03	0.131037	0.264222	0.17	0.801755	-0.01	0.03	0.03	0.04
PC aa C42:0	1,983	-0.05	0.01	7.08E-05	**0.000223**	0.17	754	-0.05	0.02	0.006725	0.030638	0.19	0.983917	0.00	0.02	-0.01	0.03
PC aa C42:1	1,983	-0.03	0.01	0.006508	**0.012911**	0.17	754	-0.05	0.02	0.017156	0.059504	0.20	0.962043	0.01	0.03	0.02	0.03
PC aa C42:2	1,983	0.05	0.01	0.000875	**0.002031**	0.24	754	0.05	0.02	0.03433	0.093834	0.30	0.998364	0.00	0.03	0.00	0.03
PC aa C42:4	1,922	-0.01	0.01	0.629828	0.710723	0.04	734	0.00	0.02	0.873776	0.905917	0.08	0.976658	-0.02	0.03	-0.01	0.03
PC aa C42:5	1,922	0.01	0.01	0.499936	0.602864	0.15	734	0.01	0.02	0.662097	0.758164	0.20	0.529174	-0.02	0.03	0.04	0.04
PC aa C42:6	1,922	0.00	0.01	0.868533	0.907071	0.08	734	0.01	0.02	0.701766	0.784702	0.10	0.899268	-0.02	0.03	0.01	0.03
PC ae C30:0	1,922	0.00	0.01	0.877573	0.907071	0.18	734	0.01	0.02	0.665705	0.758164	0.26	0.59968	-0.03	0.03	-0.06	0.03
PC ae C30:2	1,922	-0.05	0.01	4.96E-05	**0.000165**	0.11	734	-0.03	0.02	0.083522	0.193834	0.15	0.572879	-0.01	0.02	-0.05	0.03
PC ae C32:1	1,983	0.06	0.02	1.81E-05	**6.75E-05**	0.07	754	0.05	0.02	0.068076	0.161026	0.12	0.966039	0.01	0.03	0.02	0.04
PC ae C32:2	1,983	0.06	0.02	0.000367	**0.000921**	0.07	754	0.06	0.03	0.017416	0.059504	0.12	0.977406	-0.02	0.03	-0.01	0.04
PC ae C34:0	1,983	0.04	0.02	0.013391	**0.024583**	0.09	754	0.06	0.03	0.019638	0.06137	0.16	0.501162	-0.05	0.03	-0.08	0.04
PC ae C34:1	1,983	0.01	0.02	0.612845	0.697962	0.08	754	0.01	0.03	0.597487	0.72745	0.11	0.790842	-0.02	0.03	-0.05	0.04
PC ae C34:2	1,983	-0.04	0.02	0.004067	**0.008478**	0.08	754	-0.04	0.03	0.124872	0.255988	0.10	0.794049	0.01	0.03	-0.03	0.04
PC ae C34:3	1,983	0.06	0.02	0.000527	**0.001289**	0.09	754	0.04	0.03	0.181238	0.315497	0.12	0.59968	0.06	0.03	0.04	0.04
PC ae C36:0	1,922	0.16	0.02	<1E-12	**<1E-12**	0.13	734	0.13	0.03	8.37E-07	1.72E-05	0.16	0.801755	0.03	0.03	0.04	0.04
PC ae C36:1	1,983	0.00	0.02	0.940879	0.948592	0.06	754	0.04	0.03	0.08962	0.204135	0.11	0.171999	-0.06	0.03	-0.10	0.04
PC ae C36:2	1,983	-0.11	0.02	<1E-12	**<1E-12**	0.12	754	-0.06	0.02	0.020501	0.06137	0.15	0.040198	-0.04	0.03	-0.12	0.04

PC ae C36:3	1,983	-0.04	0.02	0.023764	**0.040596**	0.06	754	-0.04	0.03	0.114979	0.243836	0.07	0.891805	0.03	0.03	0.00	0.04
PC ae C36:4	1,983	0.04	0.01	0.010777	**0.020084**	0.15	754	0.04	0.02	0.122414	0.255203	0.18	0.984326	-0.01	0.03	0.00	0.04
PC ae C36:5	1,983	0.10	0.02	1E-11	**1.02E-10**	0.15	754	0.10	0.03	7.57E-05	0.000931	0.19	0.977406	0.00	0.03	0.02	0.04
PC ae C38:0	1,616	0.09	0.02	8.47E-07	**4.34E-06**	0.17	653	0.10	0.03	0.000772	0.005938	0.20	0.983917	-0.01	0.04	0.01	0.04
PC ae C38:2	1,983	-0.07	0.01	1.21E-05	**4.65E-05**	0.06	754	-0.05	0.02	0.060341	0.145529	0.09	0.49181	0.00	0.03	-0.07	0.04
PC ae C38:3	1,983	-0.09	0.02	5.86E-09	**3.79E-08**	0.08	754	-0.04	0.03	0.107934	0.232911	0.11	0.176035	-0.06	0.03	-0.10	0.04
PC ae C38:4	1,983	-0.05	0.02	0.001614	**0.003677**	0.11	754	-0.01	0.03	0.683601	0.771403	0.14	0.295845	-0.06	0.03	-0.09	0.04
PC ae C38:5	1,983	0.02	0.02	0.213677	0.288816	0.12	754	0.03	0.03	0.190163	0.321474	0.14	0.790111	-0.03	0.03	-0.05	0.04
PC ae C38:6	1,983	0.05	0.02	0.000744	**0.001759**	0.17	754	0.08	0.03	0.003658	0.018853	0.21	0.801755	-0.04	0.03	-0.03	0.04
PC ae C40:1	1,983	0.07	0.02	4.05E-05	**0.000138**	0.09	754	0.08	0.03	0.002424	0.0142	0.11	0.983917	0.01	0.04	0.00	0.04
PC ae C40:2	1,983	0.01	0.01	0.721044	0.786698	0.12	754	0.06	0.02	0.012359	0.049037	0.18	0.012344	-0.08	0.03	-0.13	0.04
PC ae C40:3	1,922	-0.05	0.01	0.000163	**0.000489**	0.10	734	-0.05	0.02	0.04763	0.119561	0.14	0.624792	-0.01	0.03	-0.05	0.03
PC ae C40:4	1,983	-0.08	0.02	2.17E-06	**9.9E-06**	0.08	754	-0.06	0.03	0.020237	0.06137	0.11	0.59968	-0.03	0.03	-0.06	0.04
PC ae C40:5	1,983	-0.03	0.02	0.030533	0.050751	0.08	754	-0.02	0.03	0.502522	0.656355	0.12	0.520145	-0.05	0.03	-0.07	0.04
PC ae C40:6	1,983	-0.06	0.02	0.000284	**0.000727**	0.17	754	0.00	0.03	0.912514	0.919993	0.20	0.158904	-0.07	0.03	-0.10	0.04
PC ae C42:1	1,938	0.01	0.02	0.557053	0.647842	0.04	741	-0.02	0.03	0.454949	0.608246	0.06	0.509718	0.07	0.04	0.07	0.04
PC ae C42:2	1,983	0.02	0.02	0.118477	0.175574	0.10	754	0.04	0.03	0.169513	0.311701	0.12	0.899268	-0.02	0.03	-0.03	0.04
PC ae C42:3	1,983	0.02	0.01	0.084541	0.128377	0.14	754	0.03	0.02	0.249364	0.398335	0.17	0.983917	-0.01	0.03	-0.01	0.03
PC ae C42:4	1,983	-0.06	0.02	0.000204	**0.000584**	0.11	754	-0.06	0.03	0.022107	0.063236	0.13	0.899268	-0.01	0.03	-0.03	0.04
PC ae C42:5	1,983	-0.06	0.02	0.000217	**0.000605**	0.08	754	-0.06	0.03	0.035412	0.094688	0.10	0.608217	-0.04	0.03	-0.06	0.04
PC ae C44:4	1,983	-0.02	0.01	0.181462	0.250785	0.06	754	-0.03	0.02	0.102852	0.229614	0.07	0.801755	0.01	0.03	0.03	0.03
PC ae C44:5	1,983	-0.01	0.01	0.399652	0.496538	0.10	754	-0.02	0.02	0.350598	0.507336	0.09	0.801755	-0.03	0.03	-0.01	0.03
PC ae C44:6	1,983	-0.04	0.01	0.007742	**0.01488**	0.09	754	-0.06	0.02	0.016888	0.059504	0.10	0.966039	-0.01	0.03	0.02	0.03
**Sphingolipids**																	
SM C16:0	1,983	0.01	0.01	0.36541	0.458627	0.05	754	0.03	0.02	0.056067	0.137924	0.07	0.501162	-0.02	0.02	-0.05	0.02
SM C16:1	1,983	0.02	0.01	0.014882	**0.026528**	0.07	754	0.04	0.02	0.020955	0.06137	0.09	0.840841	-0.02	0.02	-0.02	0.02
SM C18:0	1,983	0.04	0.01	1.07E-05	**4.25E-05**	0.07	754	0.07	0.02	1.26E-05	0.000221	0.14	0.501162	-0.03	0.02	-0.04	0.02
SM C18:1	1,983	0.01	0.01	0.178527	0.249532	0.05	754	0.04	0.02	0.015016	0.055967	0.09	0.501162	-0.04	0.02	-0.05	0.02
SM C20:2	1,983	-0.06	0.01	1.43E-06	**6.75E-06**	0.26	754	-0.04	0.02	0.036529	0.095597	0.32	0.801755	-0.02	0.03	-0.03	0.03
SM C24:0	1,983	0.04	0.01	1.27E-06	**6.26E-06**	0.05	754	0.05	0.01	0.000408	0.003587	0.06	0.983917	0.00	0.02	-0.01	0.02
SM C24:1	1,983	0.03	0.01	2.8E-05	**0.000101**	0.08	754	0.05	0.01	0.000283	0.002904	0.10	0.608217	-0.03	0.02	-0.02	0.02
SM(OH) C14:1	1,983	-0.06	0.01	3.89E-07	**2.13E-06**	0.14	754	-0.01	0.02	0.546985	0.684792	0.20	0.003291	-0.05	0.02	-0.12	0.03
SM(OH) C16:1	1,983	-0.05	0.01	2.55E-06	**1.12E-05**	0.07	754	0.01	0.02	0.639576	0.749217	0.11	4.36E-05	-0.06	0.02	-0.13	0.02
SM(OH) C22:1	1,983	-0.03	0.01	0.004525	**0.009275**	0.05	754	0.01	0.01	0.412677	0.57681	0.06	0.008559	-0.04	0.02	-0.09	0.02
SM(OH) C22:2	1,983	-0.05	0.01	1.34E-07	**8.25E-07**	0.09	754	-0.01	0.01	0.528789	0.675607	0.09	0.008219	-0.04	0.02	-0.09	0.02
SM(OH) C24:1	1,983	0.00	0.01	0.694071	0.769106	0.05	754	0.04	0.01	0.007398	0.0325	0.07	0.038822	-0.05	0.02	-0.07	0.02
**Hexoses**																	
Hexoses	1,983	-0.04	0.02	0.008591	**0.016256**	0.16	754	-0.04	0.03	0.161336	0.311701	0.22	0.98062	-0.02	0.03	-0.01	0.04

Abbreviations: β, unstandardized regression coefficient derived from multivariable linear models; SE, standard error.

For an explanation of abbreviated metabolite names, see Supplementary Table 1.

[a] Analyzed with multivariable linear regression analyses analyzing associations of alcohol consumption (ln-transformed alcohol intake + 1) as main independent variable and as dependent variables the residuals obtained from linear mixed models with Z-standardized ln-transformed metabolite concentrations as dependent variables, sex as independent variable, and random intercepts for analytical batches nested within studies. Adjusted for: sex; age (y; continuous), body mass index (kg/m^2; continuous), self-reported physical activity levels (Cambridge physical activity index(1): inactive, moderately inactive, moderately active, active, unknown), fasting status (≥6 hours, 3-5.9 hours, <3 hours, unknown), meat intake (g/day; continuous), fish intake (g/day; continuous), energy intake (kcal/day; continuous), country, and smoking status (current, former, never, unknown; not in analysis in never smokers only).

[b] Testing for statistical interaction was performed by including product terms for alcohol intake and smoking status (former and current *vs.* never smokers) and evaluating overall significance of both product terms.

[c] The analysis in the discovery set was adjusted for multiple testing using the false discovery rate (FDR) method. Statistically significant associations (FDR q-value<0.05) are depicted in bold.

Supplementary Table 7. Results of discovery and replication analysis on associations of categories of alcohol intake (heavy *vs.* light) with concentrations of metabolites included in the current analysis (n=123)[a]

	Discovery analysis (n=1,983)[b]						Replication analysis (n=991)[b]					
Metabolite	n	β	SE	p-value	FDR q-value[c]	R^2	n	β	SE	p-value	Bonf.-adj. p-value[d]	R^2
**Acylcarnitines**												
Acylcarnitine C0	1,983	0.24	0.07	0.000934	**0.002253**	0.06	991	0.14	0.11	0.194565	1	0.07
Acylcarnitine C14:1	1,956	0.25	0.07	0.00036	**0.001029**	0.09	979	0.30	0.10	0.002161	0.151261	0.14
Acylcarnitine C16	1,956	0.46	0.07	<1E-12	**<1E-12**	0.15	979	0.32	0.09	0.000699	**0.048941**	0.19
Acylcarnitine C18	1,956	0.25	0.07	0.000417	**0.00109**	0.09	979	0.05	0.10	0.569899	1	0.11
Acylcarnitine C18:1	1,956	0.29	0.07	2.09E-05	**7.83E-05**	0.17	979	0.14	0.10	0.162496	1	0.24
Acylcarnitine C18:2	1,956	0.10	0.07	0.158328	0.230313	0.18	NA					
Acylcarnitine C2	1,983	0.36	0.07	1.75E-06	**8.26E-06**	0.10	991	0.27	0.11	0.012497	0.874795	0.13
Acylcarnitine C3	1,956	-0.05	0.08	0.548656	0.648891	0.07	NA					
Acylcarnitine C4	1,852	0.07	0.07	0.373074	0.490008	0.05	NA					
Acylcarnitine C5	1,828	0.11	0.07	0.135205	0.205311	0.04	NA					
**Amino acids**												
Alanine	1,983	-0.09	0.07	0.217054	0.303383	0.11	NA					
Arginine	1,983	-0.22	0.06	0.000171	**0.000526**	0.20	989	-0.16	0.09	0.08214	1	0.23
Asparagine	1,983	0.00	0.07	0.982346	0.990398	0.13	NA					
Citrulline	1,983	-0.30	0.07	1.98E-05	**7.83E-05**	0.10	989	-0.28	0.10	0.004686	0.327996	0.15
Glutamine	1,983	-0.12	0.08	0.100806	0.158964	0.05	NA					
Glutamate	1,983	0.20	0.06	0.001334	**0.003095**	0.26	989	0.08	0.09	0.377721	1	0.26
Glycine	1,983	-0.12	0.06	0.048374	0.079333	0.11	NA					
Histidine	1,983	0.05	0.07	0.480328	0.590804	0.08	NA					
Isoleucine	1,983	0.03	0.07	0.724893	0.795585	0.08	NA					
Leucine	1,982	0.03	0.07	0.652553	0.750132	0.08	NA					
Lysine	1,983	-0.02	0.07	0.798641	0.846835	0.06	NA					
Methionine	1,983	0.06	0.07	0.374478	0.490008	0.08	NA					
Ornithine	1,983	-0.03	0.07	0.686945	0.771537	0.17	NA					
Phenylalanine	1,983	0.02	0.06	0.730903	0.795585	0.10	NA					
Proline	1,983	-0.06	0.08	0.437499	0.554767	0.09	NA					
Serine	1,983	-0.15	0.06	0.01538	**0.029558**	0.11	989	-0.31	0.08	0.000192	**0.013473***	0.13
Threonine	1,983	0.00	0.07	0.994377	0.994377	0.09	NA					
Tryptophan	1,983	0.02	0.07	0.743788	0.802508	0.06	NA					
Tyrosine	1,983	0.22	0.07	0.001979	**0.004346**	0.11	989	0.22	0.10	0.028657	1	0.12
Valine	1,983	-0.01	0.07	0.912314	0.936033	0.08	NA					
t4-hydroxyproline	1,774	0.18	0.08	0.027244	**0.047872**	0.12	871	0.24	0.11	0.031068	1	0.13
**Biogenic amines**												
ADMA	1,930	-0.07	0.07	0.317885	0.429669	0.04	NA					
Creatine	1,983	0.05	0.07	0.444951	0.555297	0.04	NA					
Kynurenine	1,983	-0.03	0.07	0.689992	0.771537	0.10	NA					
Taurine	1,983	0.08	0.05	0.161631	0.23117	0.32	NA					
**Glycerophospholipids**												
LysoPC a C16:0	1,983	0.36	0.06	4.3E-09	**3.31E-08**	0.16	991	0.20	0.08	0.017818	1	0.13
LysoPC a C16:1	1,983	0.52	0.07	<1E-12	**<1E-12**	0.15	991	0.49	0.09	9.12E-08	**6.38E-06**	0.14
LysoPC a C17:0	1,983	-0.42	0.06	<1E-12	**<1E-12**	0.20	991	-0.57	0.09	1E-10	**7E-09**	0.19
LysoPC a C18:0	1,983	-0.04	0.06	0.503161	0.61276	0.14	NA					

LysoPC a C18:1	1,983	0.25	0.06	0.00011	**0.000357**	0.15	991	0.19	0.09	0.035016	1	0.15
LysoPC a C18:2	1,983	-0.01	0.07	0.913203	0.936033	0.15	NA					
LysoPC a C20:3	1,983	0.18	0.07	0.008181	**0.016497**	0.09	991	0.29	0.09	0.002295	0.160657	0.1
LysoPC a C20:4	1,983	0.29	0.07	2.1E-05	**7.83E-05**	0.11	991	0.29	0.09	0.000776	0.054329	0.12
PC aa C28:1	1,983	0.07	0.06	0.242812	0.331843	0.17	NA					
PC aa C30:0	1,983	0.35	0.07	7.9E-08	**4.63E-07**	0.18	991	0.48	0.09	2.1E-07	**1.47E-05**	0.23
PC aa C32:0	1,983	0.47	0.07	<1E-12	**<1E-12**	0.11	991	0.55	0.09	1.5E-09	**1.05E-07**	0.16
PC aa C32:1	1,983	0.82	0.07	<1E-12	**<1E-12**	0.25	991	0.93	0.10	<1E-12	**<1E-12**	0.28
PC aa C32:2	1,983	0.26	0.07	0.000191	**0.000573**	0.13	991	0.38	0.10	0.000121	**0.008451**	0.18
PC aa C32:3	1,983	-0.20	0.06	0.000894	**0.0022**	0.20	991	-0.32	0.08	0.000177	**0.012421**	0.22
PC aa C34:1	1,983	0.69	0.07	<1E-12	**<1E-12**	0.12	991	0.61	0.09	<1E-12	**<1E-12**	0.14
PC aa C34:2	1,983	0.15	0.03	2.52E-06	**1.15E-05**	0.04	991	0.11	0.04	0.011269	0.788855	0.04
PC aa C34:3	1,983	0.32	0.06	9.7E-07	**4.77E-06**	0.24	991	0.40	0.09	4.8E-06	**0.000336**	0.29
PC aa C34:4	1,983	0.45	0.07	5E-10	**4.73E-09**	0.13	991	0.55	0.10	9.67E-08	**6.77E-06**	0.17
PC aa C36:0	1,983	0.13	0.06	0.031656	0.054079	0.16	NA					
PC aa C36:1	1,983	0.45	0.07	1E-09	**8.79E-09**	0.06	991	0.38	0.10	0.00019	**0.013315***	0.06
PC aa C36:2	1,983	0.07	0.06	0.21501	0.303383	0.03	NA					
PC aa C36:3	1,983	0.22	0.07	0.001966	**0.004346**	0.06	991	0.24	0.10	0.01411	0.987668	0.1
PC aa C36:4	1,983	0.63	0.07	<1E-12	**<1E-12**	0.13	991	0.66	0.09	<1E-12	**<1E-12**	0.18
PC aa C36:5	1,983	0.56	0.07	<1E-12	**<1E-12**	0.18	991	0.55	0.10	7.49E-08	**5.24E-06**	0.22
PC aa C36:6	1,983	0.33	0.07	6.74E-06	**2.86E-05**	0.14	991	0.31	0.10	0.001942	0.135946	0.18
PC aa C38:0	1,983	-0.12	0.06	0.062112	0.099217	0.26	NA					
PC aa C38:3	1,983	0.11	0.07	0.125489	0.192939	0.13	NA					
PC aa C38:4	1,983	0.30	0.07	4.93E-05	**0.000178**	0.11	991	0.31	0.10	0.003632	0.254206	0.15
PC aa C38:5	1,983	0.44	0.08	7E-09	**4.53E-08**	0.11	991	0.47	0.11	1.15E-05	**0.000805**	0.12
PC aa C38:6	1,983	0.38	0.07	1.55E-07	**8.67E-07**	0.18	991	0.25	0.10	0.014329	1	0.21
PC aa C40:2	1,922	-0.16	0.07	0.030152	0.052236	0.14	NA					
PC aa C40:3	1,922	-0.03	0.07	0.639251	0.748837	0.12	NA					
PC aa C40:4	1,983	0.33	0.07	4.39E-06	**1.93E-05**	0.12	991	0.44	0.11	4.31E-05	**0.003019***	0.12
PC aa C40:5	1,983	0.24	0.07	0.00041	**0.00109**	0.16	991	0.35	0.10	0.000369	**0.025824***	0.17
PC aa C40:6	1,983	0.10	0.07	0.153283	0.227155	0.15	NA					
PC aa C42:0	1,983	-0.23	0.05	1.1E-05	**4.52E-05**	0.17	991	-0.16	0.07	0.030369	1	0.16
PC aa C42:1	1,983	-0.14	0.05	0.006499	**0.013324**	0.17	991	-0.11	0.07	0.137027	1	0.17
PC aa C42:2	1,983	0.16	0.06	0.009574	**0.018994**	0.24	991	0.15	0.08	0.064457	1	0.26
PC aa C42:4	1,922	0.09	0.06	0.159159	0.230313	0.04	NA					
PC aa C42:5	1,922	-0.01	0.07	0.907776	0.936033	0.15	NA					
PC aa C42:6	1,922	-0.02	0.06	0.727294	0.795585	0.08	NA					
PC ae C30:0	1,922	-0.10	0.06	0.107673	0.167644	0.19	NA					
PC ae C30:2	1,922	-0.28	0.05	4.39E-08	**2.7E-07**	0.12	959	-0.34	0.07	1.94E-06	**0.000136**	0.13
PC ae C32:1	1,983	0.20	0.07	0.002998	**0.006469**	0.07	991	0.23	0.09	0.014133	0.989303	0.11
PC ae C32:2	1,983	0.20	0.07	0.004681	**0.009759**	0.07	991	0.06	0.09	0.503245	1	0.08
PC ae C34:0	1,983	0.06	0.07	0.422491	0.541317	0.09	NA					
PC ae C34:1	1,983	0.01	0.07	0.84714	0.890583	0.08	NA					
PC ae C34:2	1,983	-0.23	0.07	0.000858	**0.002153**	0.08	991	-0.25	0.10	0.009153	0.640682	0.1
PC ae C34:3	1,983	0.15	0.07	0.040857	0.068841	0.09	NA					
PC ae C36:0	1,922	0.59	0.07	<1E-12	**<1E-12**	0.12	959	0.53	0.10	8.55E-08	**5.98E-06**	0.12
PC ae C36:1	1,983	-0.04	0.07	0.538655	0.643249	0.07	NA					
PC ae C36:2	1,983	-0.52	0.07	<1E-12	**<1E-12**	0.13	991	-0.64	0.10	1E-10	**7E-09**	0.14
PC ae C36:3	1,983	-0.17	0.07	0.0194	**0.035616**	0.06	991	-0.17	0.10	0.089515	1	0.06

PC ae C36:4	1,983	0.17	0.07	0.011719	**0.022879**	0.16	991	0.27	0.09	0.004587	0.321122	0.2
PC ae C36:5	1,983	0.42	0.07	5E-10	**4.73E-09**	0.15	991	0.47	0.09	5.6E-07	**3.92E-05**	0.18
PC ae C38:0	1,616	0.27	0.08	0.000833	**0.002134**	0.17	811	0.16	0.10	0.118651	1	0.22
PC ae C38:2	1,983	-0.25	0.07	0.000162	**0.00051**	0.06	991	-0.17	0.09	0.07015	1	0.09
PC ae C38:3	1,983	-0.35	0.07	3.85E-07	**1.97E-06**	0.08	991	-0.36	0.10	0.000374	**0.026211**	0.1
PC ae C38:4	1,983	-0.17	0.07	0.017794	**0.033162**	0.11	991	-0.11	0.10	0.278653	1	0.14
PC ae C38:5	1,983	0.10	0.07	0.142878	0.214317	0.12	NA					
PC ae C38:6	1,983	0.14	0.07	0.04153	0.069029	0.17	NA					
PC ae C40:1	1,983	0.29	0.07	7.94E-05	**0.000277**	0.09	991	0.20	0.10	0.045864	1	0.08
PC ae C40:2	1,983	-0.08	0.07	0.236843	0.327322	0.12	NA					
PC ae C40:3	1,922	-0.25	0.06	8.6E-05	**0.000286**	0.10	959	-0.25	0.10	0.009465	0.662555	0.12
PC ae C40:4	1,983	-0.25	0.07	0.000371	**0.001036**	0.08	991	-0.11	0.11	0.31584	1	0.09
PC ae C40:5	1,983	-0.16	0.07	0.025056	**0.044889**	0.08	991	-0.07	0.10	0.474978	1	0.06
PC ae C40:6	1,983	-0.35	0.07	3.22E-07	**1.72E-06**	0.18	991	-0.40	0.10	7.4E-05	**0.005178**	0.2
PC ae C42:1	1,938	0.17	0.08	0.025182	**0.044889**	0.05	975	0.25	0.10	0.01427	0.99891	0.05
PC ae C42:2	1,983	0.06	0.07	0.396672	0.513586	0.10	NA					
PC ae C42:3	1,983	0.04	0.06	0.514523	0.620454	0.14	NA					
PC ae C42:4	1,983	-0.24	0.07	0.000407	**0.00109**	0.11	991	-0.16	0.10	0.096208	1	0.12
PC ae C42:5	1,983	-0.23	0.07	0.001243	**0.00294**	0.08	991	-0.09	0.10	0.367984	1	0.07
PC ae C44:4	1,983	-0.02	0.06	0.662712	0.754755	0.06	NA					
PC ae C44:5	1,983	-0.03	0.06	0.648538	0.750132	0.10	NA					
PC ae C44:6	1,983	-0.16	0.06	0.015867	**0.030026**	0.09	991	0.01	0.09	0.92325	1	0.08
**Sphingolipids**												
SM C16:0	1,983	0.00	0.04	0.945382	0.961008	0.05	NA					
SM C16:1	1,983	0.04	0.04	0.3219	0.430366	0.07	NA					
SM C18:0	1,983	0.13	0.04	0.003071	**0.006512**	0.07	991	-0.05	0.06	0.379058	1	0.09
SM C18:1	1,983	0.01	0.04	0.772134	0.825848	0.05	NA					
SM C20:2	1,983	-0.32	0.05	5.1E-09	**3.69E-08**	0.27	991	-0.26	0.07	9.64E-05	**0.006746**	0.3
SM C24:0	1,983	0.15	0.04	8.1E-05	**0.000277**	0.05	991	0.19	0.05	0.00031	**0.021677**	0.08
SM C24:1	1,983	0.12	0.04	0.001739	**0.00396**	0.08	991	0.17	0.05	0.000902	0.063164	0.14
SM(OH) C14:1	1,983	-0.30	0.05	5.5E-09	**3.76E-08**	0.15	991	-0.35	0.07	9.23E-07	**6.46E-05**	0.16
SM(OH) C16:1	1,983	-0.27	0.05	4.2E-09	**3.31E-08**	0.08	991	-0.38	0.06	3.3E-09	**2.31E-07**	0.12
SM(OH) C22:1	1,983	-0.15	0.04	0.000236	**0.000693**	0.05	991	-0.14	0.05	0.008197	0.573816	0.07
SM(OH) C22:2	1,983	-0.25	0.04	2E-10	**2.24E-09**	0.10	991	-0.25	0.05	4.58E-06	**0.000321**	0.11
SM(OH) C24:1	1,983	-0.03	0.04	0.446946	0.555297	0.05	NA					
**Hexoses**												
Hexoses	1,983	-0.13	0.07	0.055507	0.089833	0.16	NA					

Abbreviations: β, unstandardized regression coefficient derived from multivariable linear models; Bonf.-adj. p-value, Bonferroni-adjusted p-value; NA, not applicable (metabolites that were not significant in the discovery analysis were not taken forward to the replication analysis); SE, standard error.

For an explanation of abbreviated metabolite names, see Supplementary Table 1.

[a] Analyzed with multivariable linear regression analyses analyzing associations of heavy (≥40 g/day) *vs.* light drinking (0.1-4.9 g/day) as independent variable and as dependent variables the residuals obtained from linear mixed models with Z-standardized ln-transformed metabolite concentrations as dependent variables, sex as independent variable, and random intercepts for analytical batches nested within studies. Adjusted for: sex; age (y; continuous), body mass index (kg/m^2; continuous), self-reported physical activity levels (Cambridge physical activity index(1): inactive, moderately inactive, moderately active, active, unknown), fasting status (≥6 hours, 3-5.9 hours, <3 hours, unknown), meat intake (g/day; continuous), fish intake (g/day; continuous), energy intake (kcal/day; continuous), country, and smoking status (current, former, never, unknown).

[b] The discovery and replication set were taken as random samples without replacement of 66.7% and 33.3% of the total dataset, respectively.

[c] The analysis in the discovery set was adjusted for multiple testing using the false discovery rate (FDR) method. Statistically significant associations (FDR q-value<0.05) are depicted in bold.

[d] The analysis in the replication set was adjusted for multiple testing using Bonferroni correction. Statistically significant associations (Bonferroni-adjusted p-value<0.05) are depicted in bold.

Asterisk denotes that the metabolite was not identified in the main analysis with continuous ln-transformed alcohol intake.

References

1. Wareham, N.J., R.W. Jakes, K.L. Rennie, J. Schuit, J. Mitchell, S. Hennings, and N.E. Day, *Validity and repeatability of a simple index derived from the short physical activity questionnaire used in the European Prospective Investigation into Cancer and Nutrition (EPIC) study.* Public Health Nutr, 2003. **6**(4): p. 407-13.
